# Boronic acid inhibitors of penicillin-binding protein 1b: serine and lysine labelling agents

**DOI:** 10.1080/14756366.2024.2305833

**Published:** 2024-02-27

**Authors:** Levente Kollár, Katarina Grabrijan, Martina Hrast Rambaher, Krištof Bozovičar, Tímea Imre, György G. Ferenczy, Stanislav Gobec, György M. Keserű

**Affiliations:** a Medicinal Chemistry Research Group, Research Centre for Natural Sciences, Budapest, Hungary; b Department of Organic Chemistry and Technology, Faculty of Chemical Technology and Biotechnology, Budapest University of Technology and Economics, Budapest, Hungary; c Faculty of Pharmacy, University of Ljubljana, Ljubljana, Slovenia; d MS Metabolomics Research Group, Research Centre for Natural Sciences, Budapest, Hungary

**Keywords:** Penicillin-binding proteins, covalent inhibitors, boronic acids, serine labelling, lysine labelling

## Abstract

Penicillin-binding proteins (PBPs) contribute to bacterial cell wall biosynthesis and are targets of antibacterial agents. Here, we investigated PBP1b inhibition by boronic acid derivatives. Chemical starting points were identified by structure-based virtual screening and aliphatic boronic acids were selected for further investigations. Structure–activity relationship studies focusing on the branching of the boron-connecting carbon and quantum mechanical/molecular mechanical simulations showed that reaction barrier free energies are compatible with fast reversible covalent binding and small or missing reaction free energies limit the inhibitory activity of the investigated boronic acid derivatives. Therefore, covalent labelling of the lysine residue of the catalytic dyad was also investigated. Compounds with a carbonyl warhead and an appropriately positioned boronic acid moiety were shown to inhibit and covalently label PBP1b. Reversible covalent labelling of the catalytic lysine by imine formation and the stabilisation of the imine by dative N–B bond is a new strategy for PBP1b inhibition.

## Graphical abstract

Penicillin-binding protein (PBP) 1b is an antibacterial target with a serine-lysine catalytic dyad. Covalent boronic acid inhibitors targeting the serine residue and compounds containing both a boronic acid moiety and a carbonyl group targeting the lysine residue with a concomitant dative N–B bond formation were studied. Structure-activity studies were combined with QM/M free energy calculations to explore the mechanism of covalent inhibition.

**Figure:**
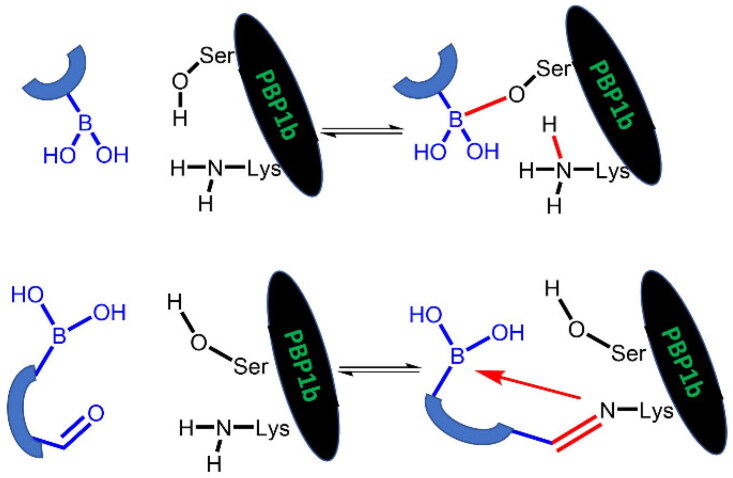

## Introduction

Antibiotic resistance has become one of the major challenges in healthcare today. It was predicted in 2016 that without finding an appropriate solution, drug-resistant bacterial infections are going to cause the death of 10 million people per year by 2050^1^. There are several causes behind the antibiotic resistance crisis, the most important being overuse, inappropriate prescribing, and extensive use in agriculture. Additionally, the research and development of new antibiotic agents by the pharmaceutical industry have stalled due to economic circumstances and regulatory obstacles^2^. The problem could be significantly reduced through various regulatory measures, but part of the real solution is the research of new antibacterial agents^3^.

Antibiotics can be divided into six categories based on the mechanism of action. They can affect DNA replication, RNA synthesis, protein synthesis (50S or 30S subunit inhibitors), cell wall biosynthesis, cell membrane biosynthesis, and fatty acid synthesis^4^^,^^5^. Inhibitors of cell wall biosynthesis have proven to be one of the most effective and most widely used classes of antibiotics; notable examples are β-lactams and glycopeptides^6^. The discovery of the first β-lactam antibiotic (Penicillin G) can be considered an iconic landmark of modern chemotherapy, and penicillins have since been by far the most extensively studied compounds of the β-lactam type. Nevertheless, cephalosporins, monobactams, carbapenems, and penems are also highly valuable members of β-lactams^7^.

Penicillin-binding proteins (PBPs) have an essential role in peptidoglycan biosynthesis, maturation and recycling^8^^,^^9^. Peptidoglycan is a polymer, that forms a mesh-like layer, thus building the cell wall, which is indispensable for the bacteria to resist intracellular pressure and to protect the microorganism. It contains glycan chains consisting of *β*-(1,4)-linked *N*-acetylglucosamine and *N*-acetylmuramic acid cross-linked by short peptides (3–5 amino acids) attached to the *N*-acetylmuramic acid^10^^,^^11^. PBPs have diverse catalytic activity. In general, they are responsible for the polymerisation of the glycan strand (transglycosylation) and the cross-linking (transpeptidation). Furthermore, some of them are able to catalyse the hydrolysis of the terminal D-alanine of the short stem peptides (DD-carboxypeptidation), and there are PBPs which exert the reverse activity of transpeptidation, namely the hydrolysis of the peptide bond in the cross-linking region (endopeptidation)^8^.

Bacteria contain several PBPs; they can be basically divided into two categories: high molecular mass (HMM) PBPs (generally > 60 kDa) and low molecular mass (LMM) PBPs. The former is responsible for transglycosylase and transpeptidase activities^12^^,^^13^, the latter commonly possess DD-carboxypeptidase activity^14^. Successful sequencing of a couple of bacterial genomes has determined the number of PBPs possessed by several bacteria. For example, *Escherichia coli* contains 12 PBPs, out of these PBP1a and PBP1b are the main transpeptidases and transglycosylases.

There are six known PBPs in *Streptococcus pneumoniae*, three of which are HMM PBPs: Class A, which possesses both the TP and GT domains (PBP1a, PBP1b, and PBP2a), and Class B, which possesses only the TP domain (PBP2b and PBP2x), as well as a LMM PBP, the D,D-carboxypeptidase PBP3^13^^,^^15^. While the absence of PBP2x and PBP2b is lethal to bacteria, the individual absence of PBP1a, PBP1b, and PBP2a is not critical for their survival^16^^,^^17^. PBPs are membrane-associated molecules and as such are prone to aggregation and instability in a buffer environment. PBP1b exists in a highly soluble, stable, and functional form that is an exception among PBPs and is important for the use of high-throughput biochemical inhibition assays. In addition, the TP domain is largely conserved among all enzymes that bind β-lactam antibiotics. For example, ceftobiprole can bind with high affinity to all six PBPs of *Streptococcus pneumoniae.*

PBPs can be effectively inhibited with penicillin derivatives and other β-lactams showing structural similarity with PBPs’ natural substrate (D-Ala-D-Ala end of pentapeptide precursor). β-Lactams react with serine residues in the active site of PBPs thus forming a long-lived acyl-enzyme complex that leads to the decrease of peptidoglycan cross-linking capability^8^^,^^18^^,^^19^. Although β-lactams can be considered the most important antibiotics, there are bacterial strains that develop resistance by producing mutant PBPs with low β-lactam affinity^8^ and β-lactam degrading enzymes (β-lactamases)^20^. Therefore, there is a growing need for inhibitors based on a non-β-lactam structure. An alternative to β-lactams are boronic acids, which are able to form reversible covalent tetrahedral adducts with certain nucleophilic residues (serine, threonine, and aspartic acid)^21^^,^^22^.

Boronic acids/esters are not only attracting attention because of their synthetic chemical importance, but they also play an important role in medicinal chemistry. They are being used in various fields, e.g. as anticancer, antibacterial, or antiviral agents^23–25^. The importance of these compounds is illustrated by the fact that five boronic acid/ester drugs are FDA approved. Bortezomib^26^ and ixazomib^27^ are protease inhibitors, used for the treatment of multiple myeloma. Vaborbactam is a β-lactamase inhibitor, used in combination with meropenem to act as an antibacterial agent^28^. Tavaborole is an antifungal used against onychomycosis^29^, and crisaborole is a phosphodiesterase-4 inhibitor which is used in the treatment of atopic dermatitis^30^.

Boronic acids have been investigated as PBP inhibitors by several research groups over the past decades^18^^,^^31–37^, and there have been a few recent publications on the subject^38^^,^^39^. In general, boronic acids can covalently attack serine residues. In addition, they are able to bind to PBPs in a dicovalent and tricovalent manner forming bonds with multiple serine and lysine residues^37^^,^^38^. The varying coordination of boron atom provides versatility to boronic acid inhibitors and is expected to affect both binding kinetics and affinity. In this article, we further explore the potential that lies in the inhibition of PBP1b by boronic acids and elucidate the details of the serine labelling mechanism by computational methods. After identifying the limits of inhibiting PBP1b by boronic acid labelling of the catalytic serine we also investigated the covalent labelling of the lysine residue of the Ser-Lys catalytic dyad. Compounds with appropriately positioned carbonyl and boronic acid moieties were tested. These compounds potentially form imines with lysine and the imine is stabilised by dative N-B bonds. This inhibition mechanism is expected to circumvent β-lactamase based antibiotic resistance.

## Results and discussion

### Compound screening

A virtual screening campaign of commercial boronic acids was performed. Boronic acids from the MCULE database^40^ were collected and filtered according to the following criteria: No elements other than C, H, N, O, P, S, B, F, Cl, Br, and I, heavy atom count between 10 and 30, number of rings between 1 and 5, not more than 10 rotatable bonds and not more than 3 stereo centres. This resulted in 7691 lead-like compounds. Ligand preparation (see “Experimental section”) generated various tautomeric and ionisation states and led to 10 199 compounds. Several X-ray structures of *S. pneumoniae* PBP1b complexed with various ligands available in the PDB, and 2Y2H^31^, 2XD1^41^, and 2JE5^42^ were selected for optimising the VS protocol as these complexes contain diverse ligands with well-reproduced binding poses upon self-docking. Nineteen ligands from X-ray complexes were selected including five distinct ligands (acyl-ampicillin from 5HL9^43^, cefotaxime from 2XD1^41^, a lactivicin analogue from 2JE5^42^ and two alkyl boronates from 2Y2H^31^, and 2Y2M^31^) and 14 ligands structurally related to the former five. Note that all PBP1b ligands in ChEMBL^44^ are similar to this set of 19 ligands. Non-covalent virtual screening simulations were performed using the active ligands complemented with decoys and the selected protein structures in which the active serine was mutated to glycine. The protein structure of 2XD1 was selected for the actual covalent virtual screening as this structure gave fair enrichment factors (9 at the top 3% and 6 at the top 10% ranked) in the VS simulations.

The set of 7691 compounds was subjected to a two-step hierarchical docking protocol; CovDock VS^45^^,^^46^ was applied first, and the 500 highest-scoring compounds were docked using CovDock PP^45^^,^^46^. Twenty-seven compounds were docked with a cdock affinity score of less than −7.0, of which 18 were ordered based on availability and price. Additional 8 boronic acids were available in our in-house collection.

The inhibitory activity of 26 compounds was tested in the assay^31^ employing a thioester substrate analogue at a concentration of 500 and 50 µM (Table S1). Two virtual hits (**1** and **2**) showed lower than 50% residual activity (RA) at a concentration of 500 µM in the assay and their IC_50_ values were determined (Table 1).

**Table 1.** PBP1b inhibitory activity and antimicrobial effect (MIC) of boronic acid derivatives.

Compound	Structure	RA [%] @500 µM or IC_50_ [µM]^a^	MIC *E. coli* [µM]	MIC *S. aureus* [µM]
**1**	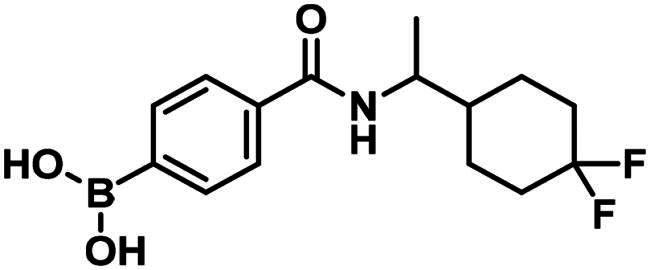	127.1 ± 14.4 µM	>250	>250
**2**	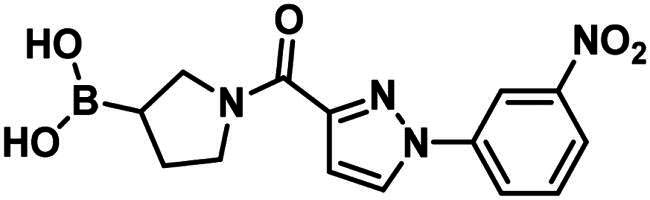	206.5 ± 21.7 µM	>250	125
**3**	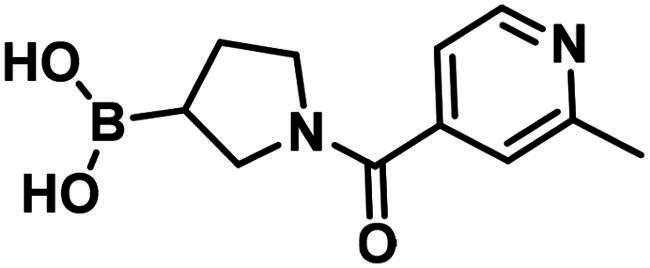	86.3 ± 8.4%	–	–
**4**	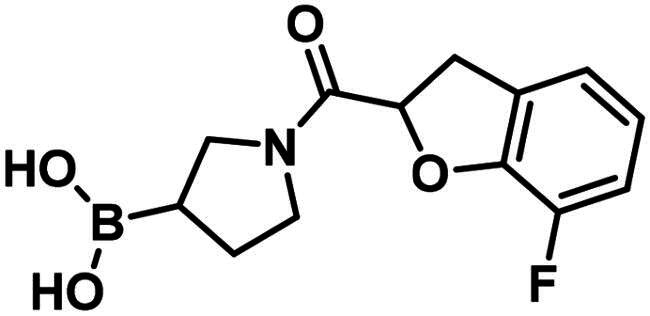	60.9 ± 7.6%	–	–
**5**	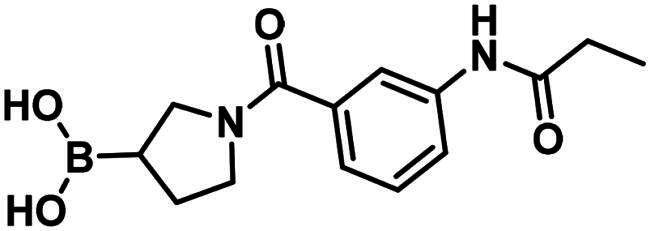	85.7 ± 9.5%	–	–
**6**	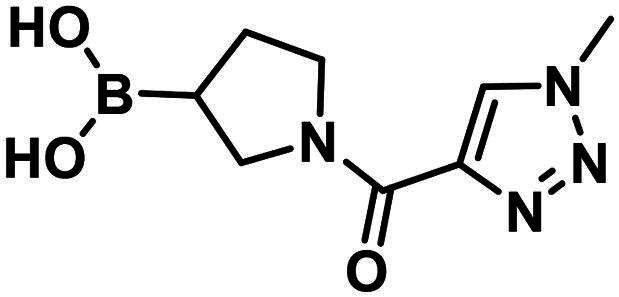	81.1 ± 1.6%	–	–
**7**	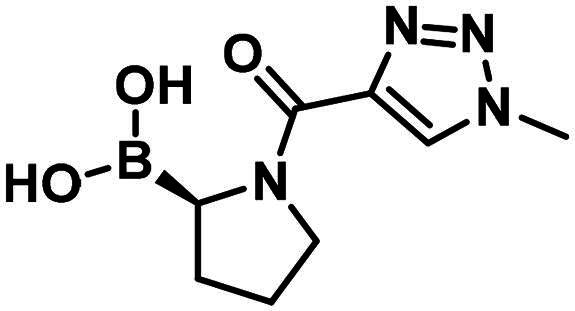	90.3 ± 3.4%^b^	–	–
**8**	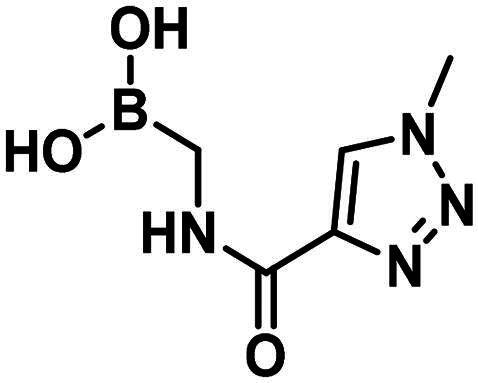	212.8 ± 15.2 µM	>250	>250
**9**	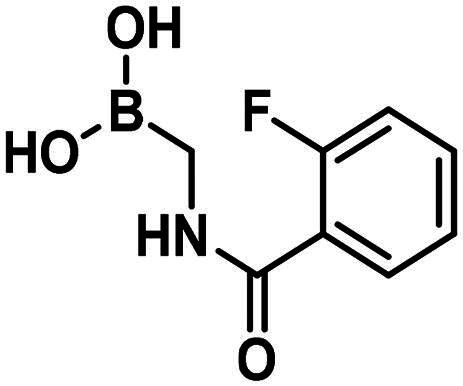	28.2 ± 7.4 µM	>250	>250

^a^Aztreonam IC_50_ found to be below the concentration of the enzyme in the assay (0.8 µM).

^b^Measured at 1 mM concentration.

#### Synthesis and structure–activity relationship of aliphatic boronic acids

Compound **1** is a boronic acid where the boron atom connects to an aromatic group. The PBP1b inhibitory potency of these types of compounds was investigated in Contreras-Martel et al.^31^ Compound **2** is an aliphatic boronic acid and we decided to investigate how the structural variation of aliphatic boronic acids affects PBP1b inhibitory activity. Compounds **3**–**6** contain variations in the amide bonding moiety, and they did not show improved activity. Similar observation was made with a changed position of the boronic acid substituent in **7**. We also investigated the effect of opening the pyrrolidine ring. A single methylene group connects the boronic acid and the amide moieties in compound **8** which showed ∼212.8 µM inhibitory activity. We note that the highly similar compound **9** exhibits an order of magnitude lower IC_50_. This latter compound is part of a linear boronic acid series studied in Contreras-Martel et al.^31^

**Scheme 1.**
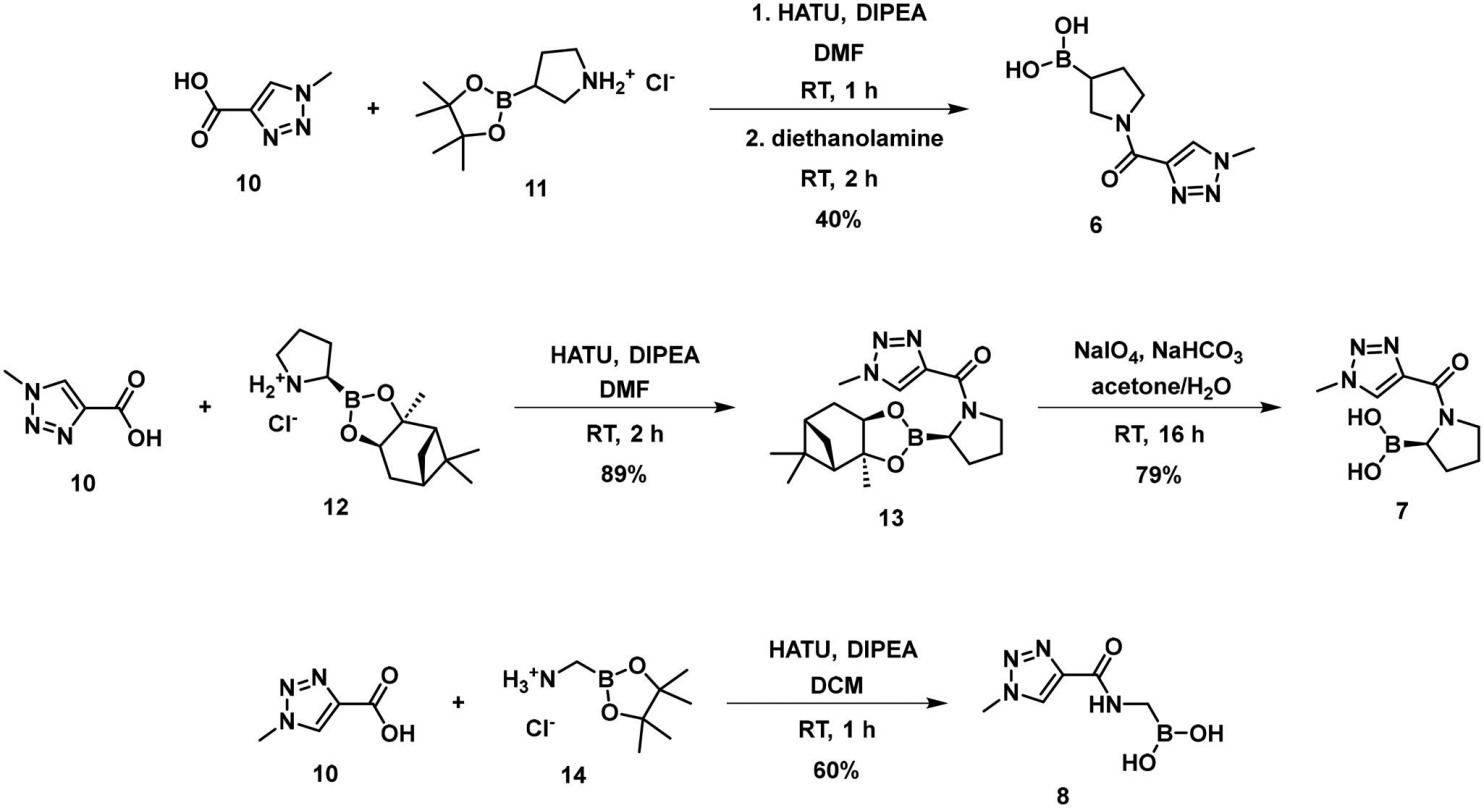
The synthesis of boroproline derivatives.

The synthesis of the boroproline derivatives is shown in Scheme 1. To obtain **6**, 1‐methyl‐1*H*‐1,2,3‐triazole‐4‐carboxylic acid (**10**) and pyrrolidine-3-boronic acid pinacol ester hydrochloride (**11**) were coupled in the presence of HATU and DIPEA. The product was not isolated, rather the hydrolysis of pinacol ester was done *one-pot* using diethanolamine, inspired by Sun et al.^47^, to get boronic acid **6**. Acylation of (*R*)-BoroPro-(+)-pinanediol hydrochloride (**12**) with **10** was done under the same conditions mentioned before, compound **13** was isolated. The pinanediol ester was cleaved in an oxidative method using NaIO_4_ and NH_4_OAc, resulting in boronic acid **7**. To examine the effect of pyrrolidine ring opening, **8** was synthesised. Carboxylic acid **10** was used to acylate aminomethylboronic acid pinacol ester hydrochloride (**14**), in the presence of HATU and DIPEA in DCM solvent. Surprisingly, the hydrolysis of pinacol ester happened *in situ* under these conditions and resulted in **8**.

### Investigation of branched boronic acids

**Scheme 2.**
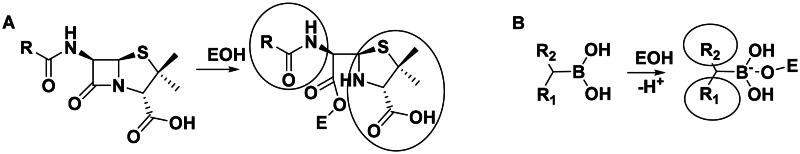
(A) Complex formation of penicillins with penicillin-binding protein. Bulky groups near the covalent enzyme-ligand bond are encircled. (B) Complex formation of branched boronic acids with penicillin-binding proteins.

The above results with the boroproline derivatives suggest that the replacement of the bulky pyrrolidine ring with the linear linker is beneficial for activity (*cf.* compound **7** with pyrrolidine ring to **8** and **9** with linear linker). However, this finding does not appear to be consistent with the inhibitory activity of β-lactam antibiotics that form complexes with PBPs that contain two, occasionally bulky groups near the covalent enzyme-ligand bond (Scheme 2A).

**Scheme 3.**
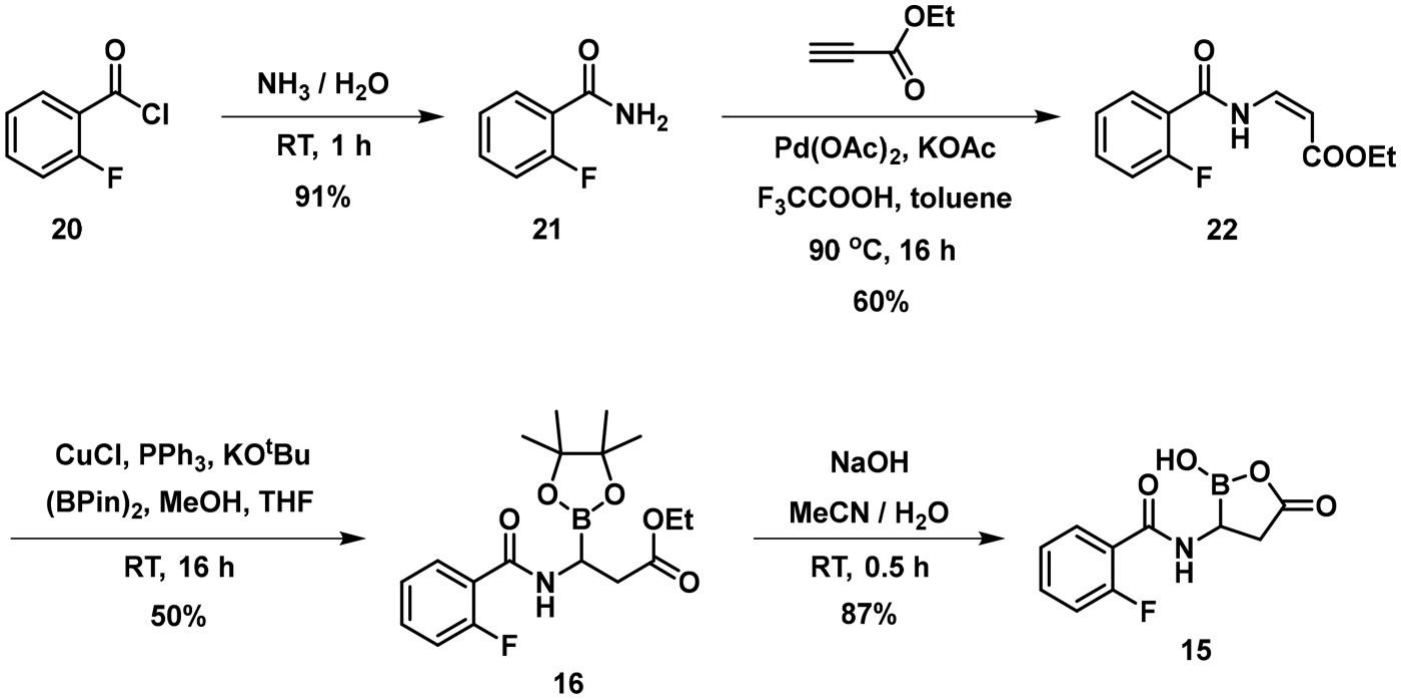
The synthesis of branched boronic acid derivatives.

**Scheme 4.**
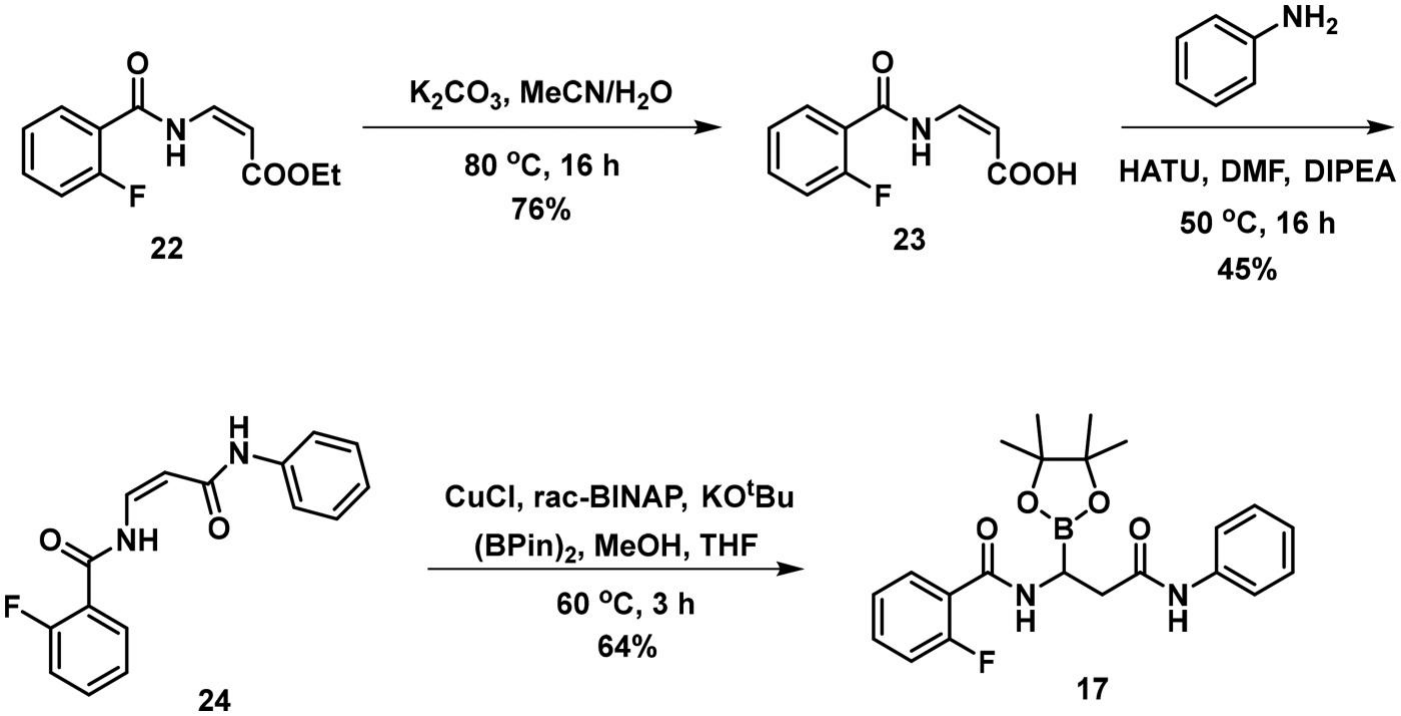
The synthesis of branched boronic acid derivatives.

Boronic acids, which branch at the carbon adjacent to the boronic acid group are expected to bind to PBP according to Scheme 2B. The proton originally bound to the active serine is transferred to a lysine residue (see below). The R_1_ and R_2_ groups in the resulting complex may occupy positions similar to those of the bulky groups in penicillin complexes.

The inhibitory activity of branched boronic acids has been investigated previously^31^^,^^48^. Interestingly, it was found that they show limited or no activity against various PBPs including *S. pneumoniae* PBP1b. It was proposed that this is the consequence of unfavourable steric interactions in the tetrahedral boronate complex^48^ and the steric hindrance in forming the analogous intermediate in the deacylation of β-lactam complexes is responsible for the effective inhibition of PBPs by β-lactams^49^. We decided to further examine this question by synthesising and assaying additional branched compounds with decreased bulkiness near the branching. This is relevant since reported branched compounds (“**E1**, **E3**-**E5**” in the Supporting Info in Contreras-Martel et al.^31^ and “**11**–**12**” in Dzhekieva et al.^48^) contain a ring next to the branching carbon. We also performed calculations on the mechanism of boronic acid-PBP1b complex formation to understand the details of the covalent bond formation and its effect on the inhibitory activity.

Table 2 shows the activities of compounds with branching on the carbon bound to the boronic acid group. The branching is part of a ring in compound **15**, while it is not involved in a ring and contains no ring close to the branching in both directions in compounds **16** and **17**. The boron atom is in a ring in **16** and **17** and we were unable to remove the protecting group and produce the corresponding free boronic acids. These compounds exhibit no or very weak PBP1b inhibition showing that the replacement of a ring by linear chains near the branching does not significantly facilitate complex formation. The presence of the protecting group in **16** and **17** may also affect the inhibitory potency; however, inhibition of PBPs by boronic acid pinacol esters has been reported^32^^,^^37^.

**Table 2.** PBP1b inhibitory activity and antimicrobial effect (MIC) of branched boronic acids.

Compound	Structure	RA [%]	MIC *E. coli* [µM]	MIC *S. aureus* [µM]
**15**	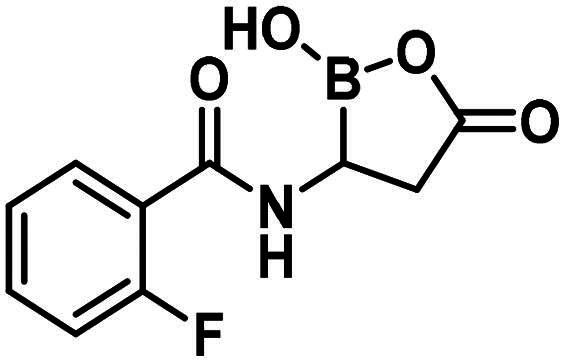	105.1 ± 0.1 @1mM	–	–
**16**	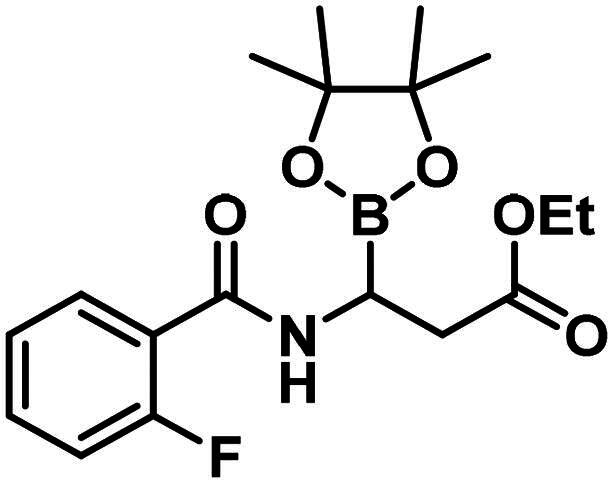	68.6 ± 3.3 @1mM	–	–
**17**	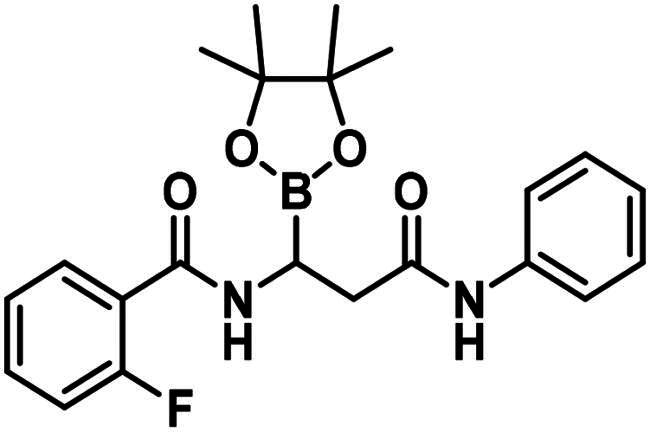	48.1 ± 2.0 @500 µM	–	–
**18**	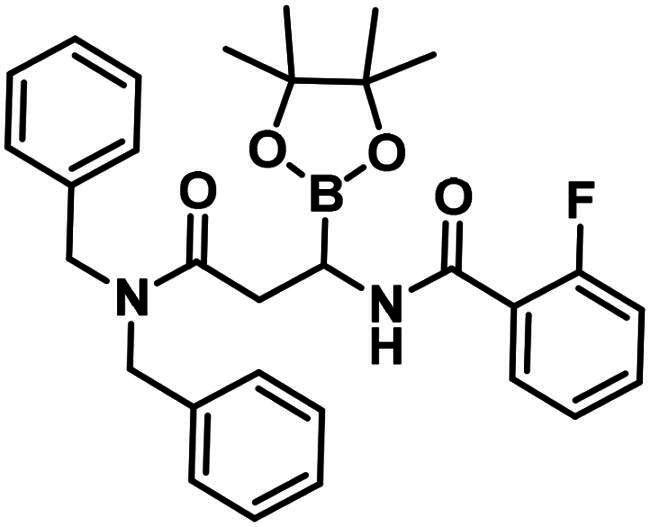	66.3 ± 7.0 @500 µM	–	–
**19**	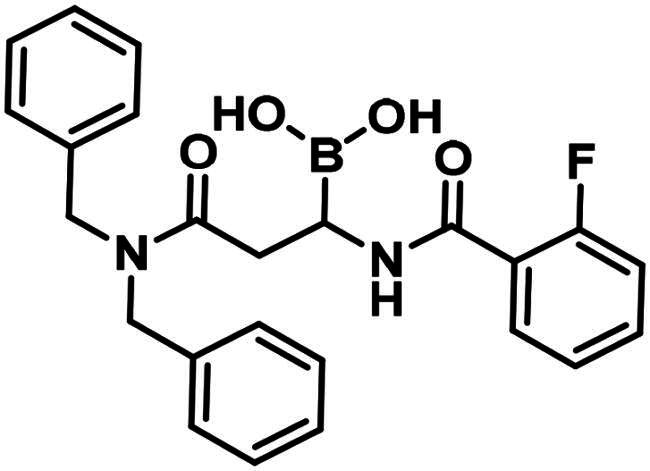	68.9 ± 8.2 @500 µM	>250	62.5

**Scheme 5.**
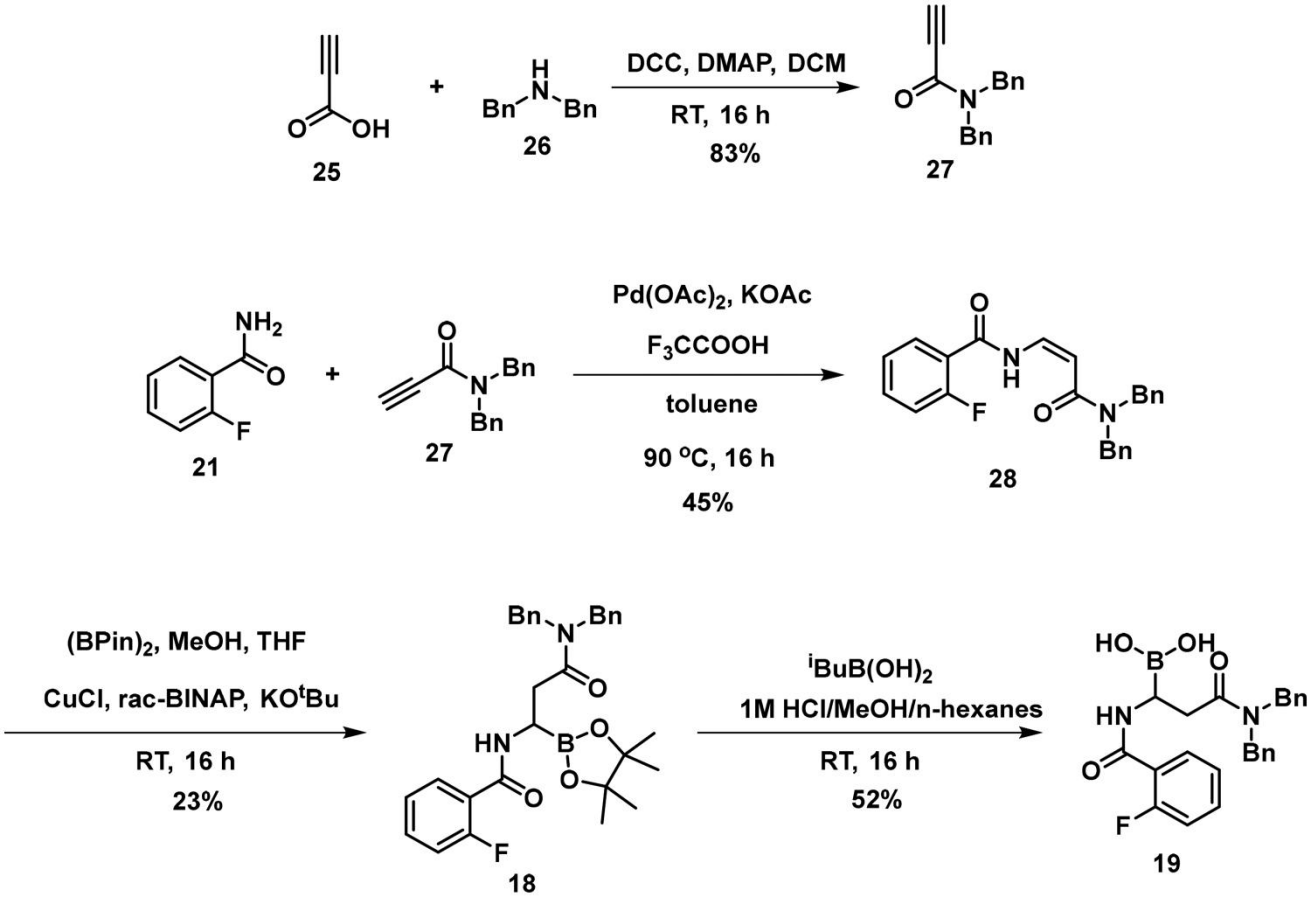
The synthesis of branched boronic acid derivatives.

The synthesis of the branched boronic acid derivatives (**15**–**19**) is shown in Schemes 3–5. 2‐Fluorobenzoyl chloride (**20**) was converted to 2-fluorobenzamide (**21**) upon the reaction with an aqueous ammonia solution. (*Z)*-Enamide (**22**) was synthesised according to the method described by Panda et al.^50^ HBpin addition was conducted using a copper-catalysed process published by Lopez et al.^51^ to produce **16**. Instead of a chiral ligand, PPh_3_ was used, because we did not intend to carry out the reaction in an enantioselective manner. After treatment of pinacol ester (**16**) with NaOH, a rapid cyclisation reaction was observed instead of hydrolysis of the carboxylic acid ester, yielding **15**.

To avoid the cyclisation reaction mentioned above, the order of the reactions was changed. (*Z)*-Enamide (**22)** was hydrolysed with the use of K_2_CO_3_, and the reaction was conducted at 80 °C for 16 h. The obtained carboxylic acid (**23**) was employed to acylate aniline in the presence of HATU and DIPEA. The received diamide (**24**) was utilised in an HBPin addition reaction, similar conditions were described earlier, although the ligand needed to be changed to *rac*-BINAP, and the reaction was done at an elevated temperature (60 °C) for 3 h, to get a significant amount of product (**17**). It is important to note that attempts were made to deprotect the boronic acid ester (**17**) by various methods, namely oxidative cleavage with NaIO_4_ and NH_4_OAc and transesterification reactions with isobutylboronic acid or boric acid in a 1 M HCl/MeOH/*n*-hexane mixture, but in each case, undesired cyclisation reactions were observed and the preparation of the free boronic acid was not successful.

To obtain a free boronic acid, we aimed to synthesise compound **19**, because it was apparent from published results, that the presence of the *N*,*N*-dibenzylamide moiety on the molecule prevents undesired cyclisation side reactions^51^. At first, propiolic acid (**25**) was utilised to acylate dibenzyl amine (**26**), DCC and DMAP facilitated the coupling to get the appropriate amide (**27**). The conditions for the preparation of the enamide (**28)** were the same as those used for the synthesis of the compound **22**. Again, to obtain **18**, *rac*-BINAP was used as a catalyst in the HBPin addition reaction. Cleavage of the pinanediol ester was successfully done with the transesterification method mentioned above, isobutylboronic acid was used in a 1 M HCl/MeOH/*n*-hexane solvent system, and the desired boronic acid (**19**) was obtained.

#### Computational investigation of Ser460 labelling by boronic acids

**Scheme 6.**
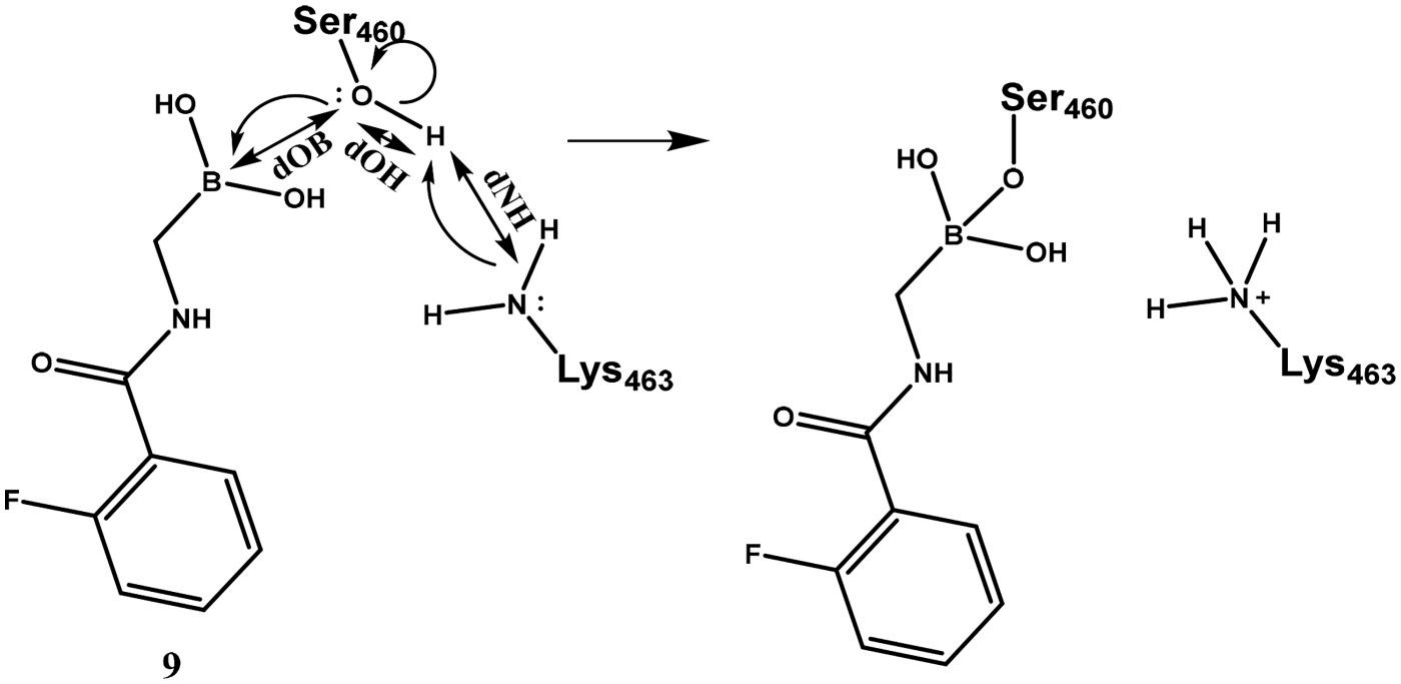
Serine activation and attack on the boronic acid. Electron movements are shown by curly arrows. Interatomic distances used for defining reaction coordinates are shown with straight arrows.

To interpret the results obtained and to explore the mechanism of boronic acid labelling of the catalytic serine the free energy profile of the reaction was investigated. The covalent inhibition of enzymes typically follows a two-step mechanism (see Figure S1 for a schematic free energy profile of the process). The first step is the non-covalent complex formation between the ligand and protein near the reactive residue. This step is governed by molecular recognition and produces a non-covalent complex that places the warhead of the ligand near the nucleophilic residue, which is a serine in PBPs. In the second step, a covalent bond is formed between the ligand and the protein, and this results in the covalent complex. In the case of boronic acids, this is a reversible reaction. The inhibitory activity of a ligand generally depends on both steps; however, a covalent bond formation with a significant reaction energy (ΔG_mc_ in Figure S1) makes the contribution of the non-covalent step negligible^52^. Concerning the noncovalent complex formation, several pieces of evidence show that the stability of the complex is weak and this might be an intrinsic property of PBPs with a highly flexible active site^53^; the K_m_ of substrates are in the high micromolar-millimolar range^54^^,^^55^ and the K_i_ (corresponding to ΔG_dm_ in Figure S1) of β-lactam inhibitors are in the millimolar range^56^. We note that we are not aware of any competitive non-covalent inhibitor, which also suggests that non-covalent recognition is unable to ensure sufficiently strong binding of small molecule ligands to the active site of PBPs. Therefore, covalent bond formation with several kcal/mol reaction energy gain is essential to achieve detectable inhibition. We performed mixed quantum mechanical/molecular mechanical molecular dynamics simulations to explore the detailed mechanism of ligand binding to the catalytic serine residue and to compare the reaction free energy profile of selected boronic acid ligands. It has been shown^57^ that the catalytic mechanism of PBP1b includes the activation of Ser460 by Lys463. Here we assumed that Ser460 activation proceeds analogously when it attacks the boronic acid moiety and we investigated how the deprotonation of Ser460-OH is connected to the nucleophilic attack on the boron atom. We performed string calculations^58^ for the mechanism in Scheme 6. Results obtained for compound **9** show that the B-O bond formation is advanced when the proton is transferred from Ser460 to Lys463 (Figure S2). This path was used to calculate the reaction free energy profile for compounds **7** and **19** (Table 3). The non-covalent complex served as the reactant state and the free energy profile yielded the reaction barrier (ΔG_tm_) and the reaction free energy (ΔG_mc_) (*cf*. Figure S1).

**Table 3.** Experimental activities, and computed barrier and reaction free energies for selected boronic acids towards PBP1b.

Compound	Structure	RA [%] or IC_50_ [µM]	Barrier^a^ [kcal/mol]	Reaction energy^b^ [kcal/mol]
**9**	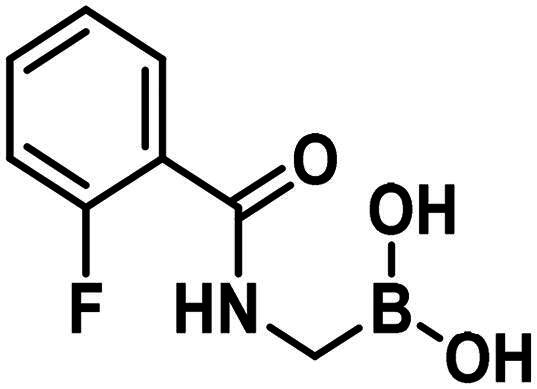	28.2 ± 7.4 µM	+6	−7
**7**	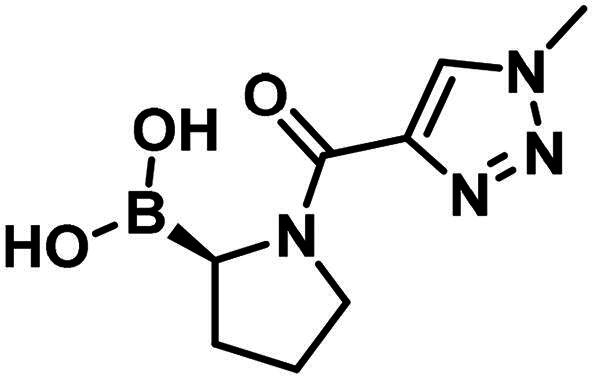	90.3 ± 3.4% @1 mM	+12	−4
**19**	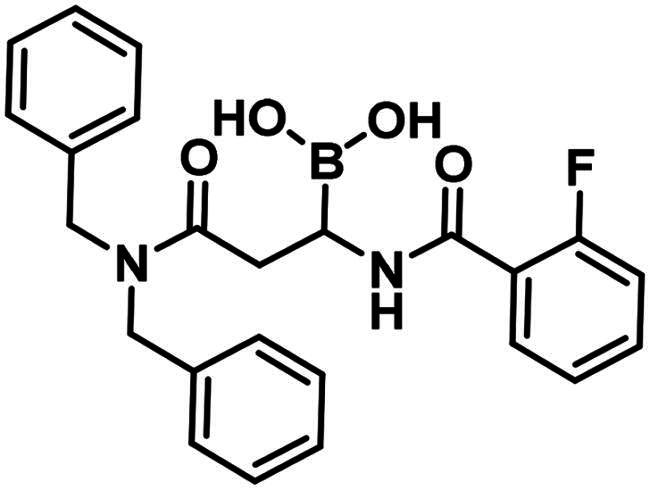	68.9 ± 8.2% @500 µM	+12	−2

^a^ΔG_tm_ in Figure S1.

^b^ΔG_mc_ in Figure S1.

The reaction free energy profile for the covalent complex formation was calculated for three compounds shown by increasing (less favourable) reaction energies in Table 3 (The free energy profiles are shown in Figure S3). The reaction barrier of 6 kcal/mol and reaction energy of −7 kcal/mol obtained for **9** are in line with reversible inhibition. The measured inhibitory activity for **9** is IC_50_ = 28.2 µM. Accepting this value as an approximation to K_i_, the corresponding free energy gain in complex formation (ΔG_dc_= ΔG_dm_+ ΔG_mc_, Figure S1) is −6.2 kcal/mol (see Equations (5) and (6) in Chatterjee et al.^52^ for the relationship between K and the free energy changes, and for the simplification for large ΔG_mc_, respectively). Then the calculated −7 kcal/mol free energy gain for the covalent step (ΔG_mc_) is a good approximation with the assumption that the non-covalent association does not significantly contribute to the free energy gain (see above). Other compounds have higher computed reaction barrier and lower computed reaction free energy gain. We note that obtained barriers are compatible with a fast reversible reaction for all compounds and the reduced activity is likely to stem from the small or missing exergonicity of the covalent complex formation. The low or marginal activity of these compounds also implies no significant affinity towards the non-covalent complex formation. The experimental and computational findings suggest that attacking the catalytic serine by boronic acids is not a viable strategy to effectively inhibit PBP1b as neither the non-covalent nor the covalent binding is able to provide significant free energy gain. Therefore, we turned our attention to the covalent modification of Lys463, the other member of the catalytic dyad.

#### Lysine targeting inhibitors

**Scheme 7.**
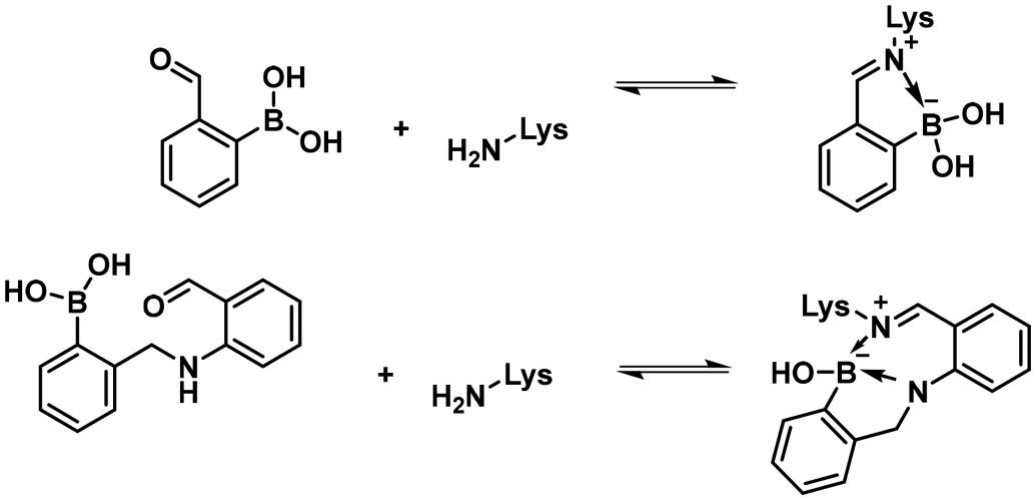
Reaction scheme of investigated carbonyl derivatives of boronic acid with lysine.

Carbonyl compounds can form imines with lysine residues and the imine bond can be stabilised by dative N-B bond formation. Compounds containing both a carbonyl group and a boronic acid moiety in suitable positions can modify lysine residues and they are useful tools in bioorthogonal chemistry^59^ and potentially in covalent inhibition^60^^,^^61^. Since PBPs contain a catalytic Ser/Lys dyad where the lysine residue activates the serine attacking the substrate carbonyl group^57^^,^^62^ it appears a viable strategy to label the lysine residue for inhibiting PBP1b. Therefore, we tested the PBP1b inhibitory activity of potential lysine labelling boronic acid derivatives (Table 4). The two types of compounds investigated are 2-formylbenzeneboronic acid derivatives that are able to form iminoboronates with lysines (**29**–**31** in Table 4) and benzenaldehydes with a more remote boronic acid moiety that can form diazaborines with lysines (**32** and **33** in Table 4) (Scheme 7).

**Table 4.** PBP1b inhibitory activity and antimicrobial effect (MIC) of potential lysine labelling boronic acid derivatives.

Compound	Structure	RA [%] or IC_50_ [µM]	MIC *E. coli* [µM]	MIC *S. aureus* [µM]
**29**	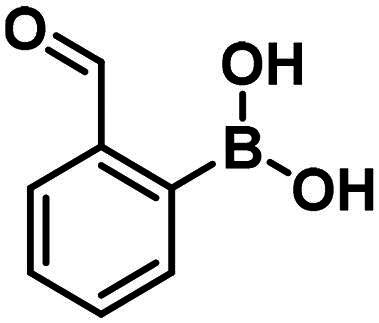	119.4 ± 6.0 µM	>250	>250
**30**	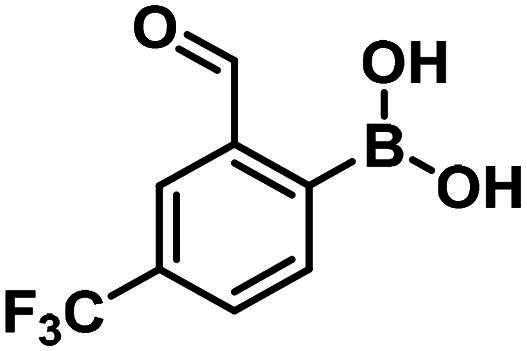	199.7 ± 15.3 µM	>250	>250
**31**	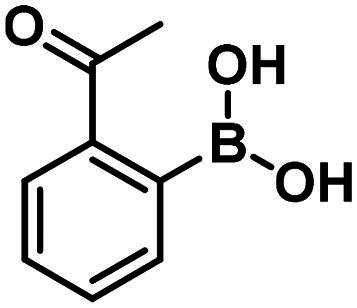	96.7 ± 0.1% @500 µM 97.4 ± 1.0% @1 mM	–	–
**32**	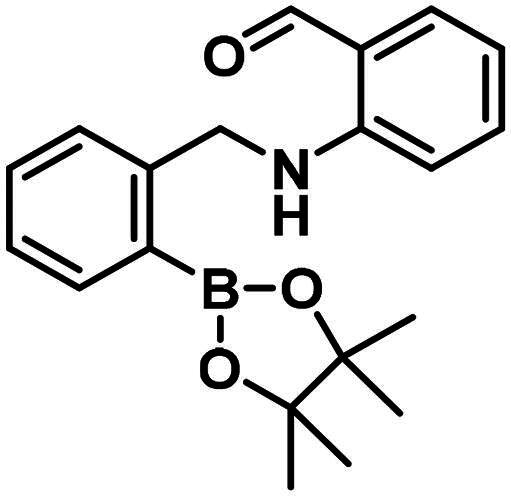	96.6 ± 0.6% @500 µM 105.2 ± 6.8% @1 mM	–	–
**33**	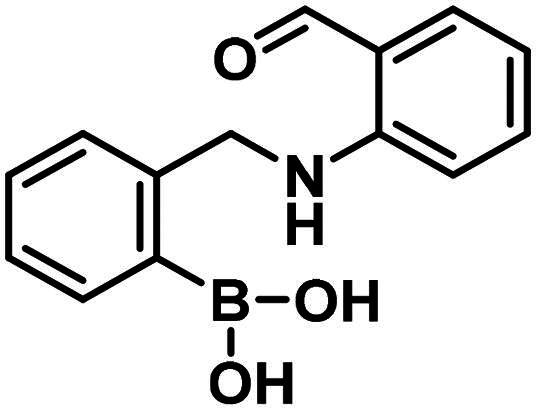	56.9 ± 0.8% @500 µM 47.1 ± 0.6% @1 mM	–	–

**Scheme 8.**
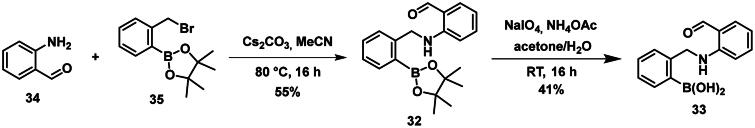
The synthesis of the diazaborine-forming warhead-containing compounds.

Compounds **29**–**31** were purchased from commercial sources, and compounds **32–33** were synthesised according to Reja et al.^61^, and are shown in Scheme 8. 2-Aminobenzaldehyde (**34**) was alkylated with 2-bromomethylphenylboronic acid pinacol ester (**35**) to give **32**. The received pinacol ester was deprotected in an oxidative method using sodium metaperiodate and ammonium acetate, thus boronic acid **33** was obtained.

The 2-formylbenzeneboronic acid derivatives **29** and **30** are inhibitors of PBP1b, while the less reactive acetyl derivative (**31**) is inactive. The aminophenyl boronic ester derivative **32** does not inhibit PBP1b at 1 mM concentration, while the corresponding boronic acid **33** exhibits slight activity in the millimolar concentration range.

The higher activity of **29** and **30** compared to **33** calls for further examination as the latter is reported to readily conjugate to lysine and lysine surrogates and to form reversible complexes with long residence time^61^. The non-covalent complex formation of **29** and **33** was examined by computational docking. The study of the non-covalent complex formation is relevant for covalent inhibitors since the non-covalent complex formation is the first step and a prerequisite for the chemical reaction leading to covalent bond formation. It was also shown that non-covalent docking of cysteine labelling ligands into Cys/Ala mutated proteins well reproduces experimental binding modes^63^. Therefore, the *N*-ethyl derivative of **29** and **33** (see Figure S4) was non-covalently docked into K463A mutated PBP1b with a restraint of positioning the terminal methyl group of the *N*-ethyl moiety in the vicinity of Ala463. The docking, however, was unsuccessful; no docking pose was obtained owing to the limited space available in the active site near to Ala463. Induced fit docking (IFD) that exploits the flexibility of the protein by allowing the movement of active site residues suggested a sensible binding pose for **29** (Figure S4). By contrast, no binding pose was obtained for **33** as the binding site was unable to accommodate the large and rigid structure of four anellated rings formed by **33** upon binding. This finding is supported by the higher inhibitory potency of **29** compared to **33** (Table 4).

**Scheme 9.**
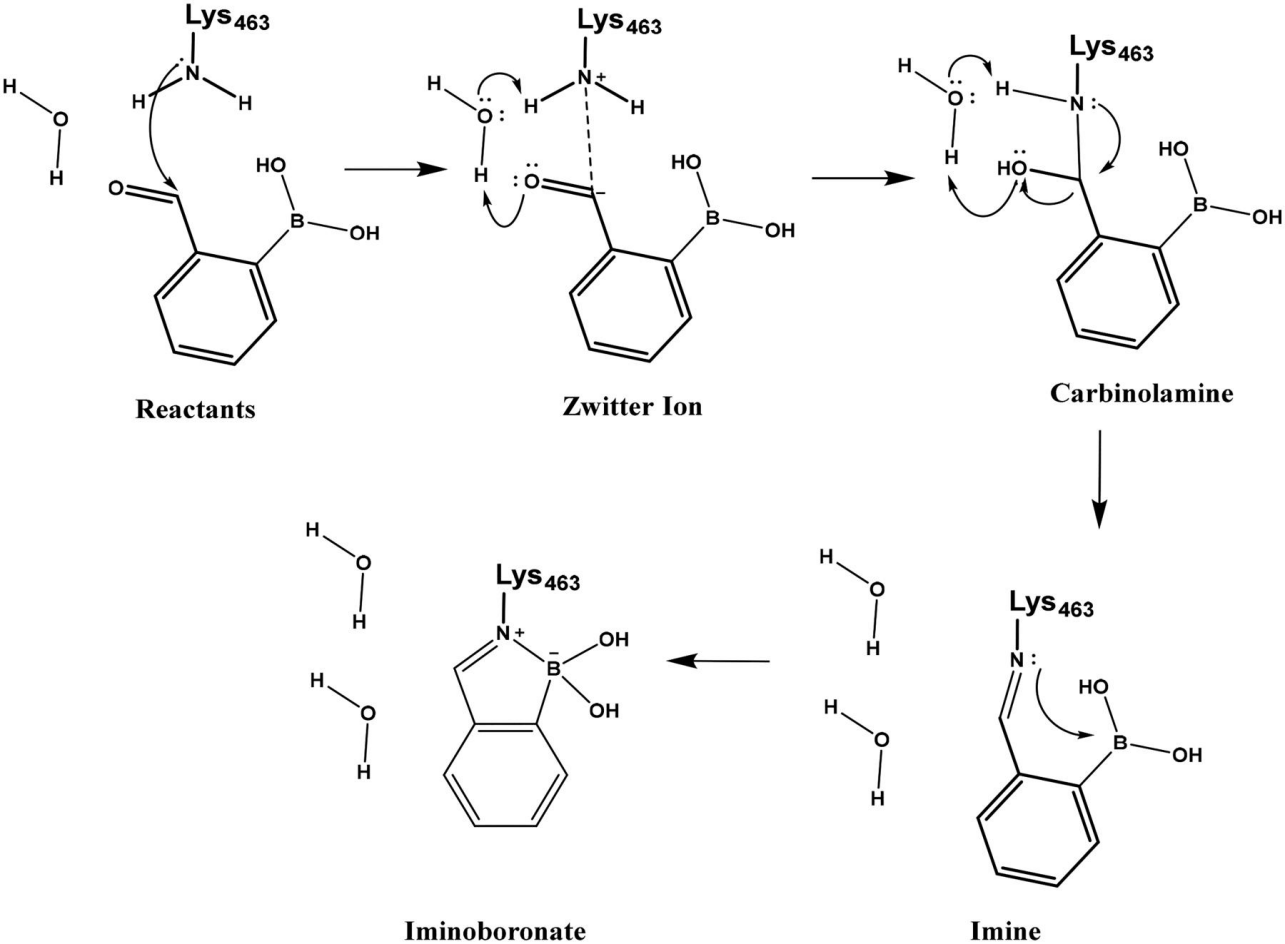
Reaction mechanism investigated by QM/MM simulations for Lys463 labelling by compound **29.**

The mechanism of binding **29** to Lys463 of PBP1b was investigated by QM/MM simulations (Scheme 9). The aim of the calculations was to see if a reasonable mechanism for the chemical reaction could be proposed that is in line with the observed activity and could serve as a basis for the computational support for inhibitor optimisation. Although we do not have direct evidence that **29** labels Lys463, the computational simulation of a sensible reaction mechanism represents further support for the site of labelling.

The above mechanism for imine formation is based on high level QM calculations for the reaction between methylamine and formaldehyde^64^^,^^65^. The four steps are assumed to proceed sequentially, and the possibility of a concerted imine bond and dative N-B bond formation was not investigated. The ability of the PM6 semiempirical QM method to reproduce the high level QM results in Hall and Smith and Chan et al.^65,66^ was checked and it was found that the zwitterion and carbinolamine formations are well described while the imine formation reaction energy is overestimated (too positive) by PM6. Altogether, the −7.5 kcal/mol reaction free energy (Figure S5) as obtained by the QM/MM simulations is sensible and the actual free energy gain may be larger as suggested by the overestimation of the imine formation energy by PM6.

Further experimental study of 2-formylbenzeneboronic acid **29** and **30** was performed to characterise their binding. No time-dependent inhibition was found for **29** and **30**, however, single labelling of PBP1b was observed in MS/MS experiments, when great excess (450-fold) of these compounds was incubated with the protein at 45 °C for 2 h (Figures S6 and S7). Since the mentioned inhibitors are known to have a reversible covalent mechanism of action, we did not attempt to determine the exact site of the labelling by digestion, because the protein-enzyme complex would in all probability have dissociated under these conditions^60^^,^^66^. The inhibitory activity and the labelling together with the known lysine labelling potential of these compounds suggest that they inhibit *via* covalently reacting with Lys463 of PBP1b.

## Conclusion

Formerly, it was shown, that boroglycine derivatives exhibit PBP1b inhibitory activity by covalently reacting with the catalytic serine, and branching on the α-carbon atom (boroalanine derivatives) diminishes activity^31^. Here we investigated linear and cyclic aliphatic boronic acid derivatives. Pyrrolidine boronic acids exhibited low or no inhibitory activity towards PBP1b and the opening of the pyrrolidine ring improved activity. We found that in addition to substituted benzamides investigated formerly, the heteroaromatic triazole ring is also compatible with the inhibition of PBP1b by boronic acid derivatives.

Computational analysis of the covalent bond formation between boronic acids and the active serine (Ser460) of PBP1b showed the detailed mechanism that includes a nucleophilic attack of Ser460 on the boron atom and the B–N bond formation is advanced when the serine proton is transferred to the neighbouring lysine. Computed reaction barriers for all investigated compounds are compatible with fast, reversible covalent complex formations, and the small or missing reaction free energy gain limits the activity of the boronic acid inhibitors. This finding appears to validate the assumption that non-covalent complex formation by the investigated boronic acid derivatives does not significantly contribute to inhibition. This is also in accordance with the formerly observed low-affinity non-covalent binding between PBPs and both their substrates^54^^,^^55^ and inhibitors^56^. This finding suggests that identifying boronic acid inhibitors against PBP1b, and presumably against other PBPs is feasible, however has limitations in terms of activity. This is demonstrated by the lack of submicromolar boronic acid PBP inhibitors in the literature, while in contrast, boronic acids exhibit outstanding activity against a number of targets^23^^,^^67^. Therefore, finding other types of antibiotic agents effective against resistant bacteria is highly needed.

Compounds with lysine targeting warheads that contain boronic acid derivatives and are able to form dative N–B bonds were shown to inhibit PBP1b. 2-formylbenzeneboronic acid derivatives were shown to covalently bind to PBP1b and they were suggested to label Lys463 of the Ser460-Lys463 catalytic dyad. This represents a new approach to inhibit PBPs and has the potential to develop reversible covalent antibacterial agents overcoming β-lactam based antibiotic resistance. Albeit lysine targeting 2-formylphenylboronic acids (**29**, **30**) show comparable activity to serine targeting boronic acids (**1**, **2**, **8**) the former are more favourable in terms of their potential for further development. These fragment-sized lysine targeting inhibitors can potentially be improved by optimising both the non-covalent recognition and the reactivity. We expect that tuning reactivity has a higher impact on the affinity owing to the observation that non-covalent binding to PBPs is generally poor (see above). Selectivity, however, is expected to be introduced by appropriate recognition elements that are compatible with binding to PBP1b close to Lys463 but deteriorate the affinity towards other potential labelling sites. These compounds combining lysine labelling with multiple boron coordination represent a new approach in inhibiting PBPs and their optimisation may lead to improved activity compounds with antibacterial effect.

## Materials and methods

### General chemistry methods

Syntheses were performed as described in Petri et al.^68^ Reagents and solvents were obtained from commercial sources (Sigma Aldrich, TCI Europe, Merck, Alfa Aesar, Combi-Blocks, and Fluorochem) and were used as received. VS compounds were purchased from Enamine and MolPort. For reactions with air- or moisture-sensitive reagents, solvents were distilled before use and these reactions were performed under a nitrogen or argon atmosphere. Flash column chromatography was performed using a CombiFlash Rf 200 instrument (Teledyne ISCO, Lincoln, NE). In the case of reversed-phase chromatography, RediSep Rf reversed-phase C18 columns (4.3 g, 26 g, 43 g, and 86 g) were used. Normal-phase flash column chromatography was performed on Merck Silica Gel 60 (particle size 0.040 − 0.063 mm; Merck, Darmstadt, Germany). Melting points were determined using a Reichelt hot stage apparatus and are uncorrected. ^1^H and ^13^C NMR spectra were recorded at 295 K on a Varian System 500 NMR spectrometer (Varian, Palo Alto, CA) or Varian System 300 NMR spectrometer operating at frequencies for ^1^H NMR at 500 MHz or 300 MHz, and for ^13^C NMR at 126 MHz or 75 MHz, respectively. The chemical shifts (δ) are reported in parts per million (ppm) and are referenced to the deuterated solvent used. The coupling constants (*J*) are given in Hz, and the splitting patterns are designated as follows: s, singlet; br s, broad singlet; d, doublet; app d, apparent doublet; dd, doublet of doublets; ddd, doublet of doublets of doublets; t, triplet; dt, doublet of triplets; td, triplet of doublets; m, multiplet. All ^13^C NMR spectra were proton decoupled. HRMS measurements (ESI^+^, ESI^–^) were performed for all new compounds. HPLC-MS measurements were performed using a Shimadzu LC-MS-2020 instrument (Shimadzu Corporation, Kyoto, Japan) equipped with a Reprospher 100 C18 (5 mm, 100 × 3 mm) column and a positive-negative double ion source (DUIS±) with a quadrupole mass spectrometer in a range of 50–1000* m/z*. Samples were eluted by gradient elution using eluent A (0.1% HCOOH in H_2_O) and eluent B (0.1% HCOOH in MeCN). The flow rate was set to 1.5 mL/min. The initial condition was 0% B eluent, followed by a linear gradient to 100% B eluent by 2 min, from 2 to 3.75 min 100% B eluent was maintained, and from 3.75 to 4.5 min back to the initial condition and maintained to 5 min. The column temperature was kept at 30 °C and the injection volume was 1 µL.

### Residual activity measurements and determination of IC_50_ values

Residual activities were determined by the ability of a potential inhibitor to hinder the hydrolysis of substrate analogue thioester (2-((benzoyl-D-alanyl)thio)acetic acid) as already described^31^. PBP1b, prepared according to literature^31^ (0.8 µM), was incubated with potential inhibitor (final concentration 500 µM) in 10 mM sodium phosphate buffer (pH = 7.0) in the presence of 100 mM D-alanine, 0.01 mg/mL BSA, and 0.01% Triton-X-100 for 60 min at 25 °C. After preincubation, the 5,5′-dithiobis-(2-nitrobenzoic acid) (DTNB) and thioester were added to each well to initiate the reaction and to obtain a final concentration of 1 mM and 5 mM, respectively. The final volume of the reaction was 150 µL. The initial rate of thioester hydrolysis was determined by measuring the absorbance at 412 nm for 30 min using a microtiter 96-well plate and Biotek Synergy H4 Hybrid microplate reader. The same assay was performed in the absence of an inhibitor. As a positive control aztreonam was used and it completely inhibits the PBP1b at 500 µM (RA [%] = 1.4 ± 0.1; IC_50_ (60 min preincubation) = 1.2 ± 0.1 µM). All experiments were run in triplicate. The ratio of the rate of reaction with inhibitor to the rate of reaction without compound expressed as a percentage gives the (RA [%] = ((vi – b)/(vo – b)) * 100, where b is the blank value for the initial rate of spontaneous hydrolysis of the thioester in the presence of the potential inhibitor and in the absence of PBP1b). The IC_50_ values were determined by measuring the reaction rates at seven different inhibitor concentrations.

### Antimicrobial testing

Antimicrobial testing was carried out by the broth microdilution method in 96-well plate format following the CLSI guidelines and European Committee for Antimicrobial Susceptibility Testing recommendations. Bacterial suspension of specific bacterial strain equivalent to 0.5 McFarland turbidity standard was diluted with cation-adjusted Mueller Hinton broth to obtain a final inoculum of 105 CFU/mL. Compounds dissolved in DMSO and inoculum were mixed together and incubated for 20 h at 35 °C. After incubation the minimal inhibitory concentration (MIC) values were determined by visual inspection as the lowest dilution of compounds showing no turbidity. The MICs were determined against *S. aureus* (ATCC 29213) and *E. coli* (ATCC 25922) bacterial strains. Tetracycline was used as a positive control on every assay plate.

### LC-MS/MS measurements

The measurements were preformed analogously to those reported in Kollár et al. and Petri et al.^68^^,^^69^ The protein stock solution (20.0 µL and 37.0 µM) was diluted with buffer solution (20.0 µL; pH 7.0; 20 mM Hepes, 100 mM NaCl, 1 mM EDTA, and 10% glycerol), then the stock solution of the inhibitor (0.66 µL, 500 mM in DMSO) was added. The sample was incubated at 45 °C for 120 min and then subjected to LC-MS/MS measurements. The molecular weights of the conjugates of PBP1b were identified using a Triple TOF 5600+ hybrid Quadrupole-TOF LC/MS/MS system (Sciex, Singapore, Woodlands) equipped with a DuoSpray IonSource coupled with a Shimadzu Prominence LC20 UFLC (Shimadzu, Kyoto, Japan) system consisting of binary pump, an autosampler and a thermostated column compartment.

Data acquisition and processing were performed using Analyst TF software version 1.7.1 (AB Sciex Instruments, Redwood City, CA). Chromatographic separation was achieved on a Merck BIOshell^TM^ 400 Å Protein C18 (75 mm × 2.1mm, 3.4 µm, 400 Å) HPLC column. Sample was eluted in gradient elution mode using solvent A (0.1% formic acid in water) and solvent B (0.1% formic acid in ACN). The initial condition was 10% B for 2 min, followed by a linear gradient to 90% B by 8 min, from 10 to 12 min 90% B was retained; and from 12 to 12.5 min back to initial condition with 10% eluent B and retained from 12.5 to 15 min. Flow rate was set to 0.5 mL/min. The column temperature was 50 °C and the injection volume was 7 µL. Nitrogen was used as the nebuliser gas (GS1), heater gas (GS2), and curtain gas with the optimum values set at 40, 45, and 40 (arbitrary units), respectively. Data were acquired in positive electrospray mode in the mass range of *m/z* = 250 to 3000, with 1 s accumulation time. The source temperature was 400 °C and the spray voltage was set to 5000 V. Declustering potential value was set to 80 V. Peak View Software^TM^ V.2.2 version 2.2 Sciex (Redwood City, CA) was used for deconvoluting the raw electrospray data to obtain the neutral molecular masses.

### Computational methods

#### Virtual screening

Preparation of the compounds for docking included the generation of tautomeric and ionisation states at pH 6–8 and the creation of 3D structures using LigPrep (Schrödinger Release 2021–3: LigPrep, Schrödinger, LLC, New York, NY, 2021). Proteins were prepared by adding H-atoms, protonating side chains at pH = 7, and refining H-bond network with the default settings of Protein Preparation Wizard (Schrödinger Release 2021–3: Protein Preparation Wizard, Schrödinger, LLC, New York, NY, 2021). Ser460 was mutated to Gly for noncovalent docking performed by Glide (Schrödinger Release 2021–3: Glide, Schrödinger, LLC, New York, NY, 2021). Covalent docking was performed by CovDock^46^.

#### Docking of lysine labelling compounds

Non-covalent docking was performed to K463A mutant of PBP1b (PDB: 2Y2K) with Glide (Schrödinger Release 2021–3: Glide, Schrödinger, LLC, New York, NY, 2021). The *N*-ethyl derivatives of compounds **29** and **33** were docked with a positional constrain requiring the terminal methyl of the *N*-ethyl group to be within 4 Angstroms of the Cβ atom of Ala463. IFD was performed with the Induced Fit Docking (IFD) protocol (Glide, Schrödinger, LLC, New York, NY, 2021–3; Prime, Schrödinger, LLC, New York, NY, 2021) using the same protein and ligands.

#### Reaction mechanism calculations

The swarm of trajectories string method^58^ implemented in AMBER (AMBER 2018, University of California, San Francisco, CA 2018) was used to explore the reaction mechanism. QM/MM calculations were performed with 16 images and 16 repeats and with a smoothing factor of 0.1. For each image 51.2 ps simulation was run with the same QM region and parameters used for the free energy calculations (see below).

#### Free energy calculation of covalent inhibition

The non-covalent complex of **9** was built from its X-ray complex structure (PDB: 2Y2K). Non-covalent complexes of other compounds in Table 3 are generated by modifying the structure of **9** using Maestro (Schrödinger Release 2021–3: Maestro, Schrödinger, LLC, New York, NY, 2021) and optimising the ligand and the sidechains in the close environment. The ligand-protein complex was immersed into an octahedral water box of TIP3P waters. The constructed systems were subject to the following relaxation protocol: 1000 steps of steepest descent minimisation and 4000 steps of conjugate gradient minimisation with constrained heavy atoms, followed by 1000 steps of conjugate gradient minimisation without constraint. 50 ps heating to 300 K, 250 ps NPT equilibration and 250 ps NVT equilibration were performed. All these steps just as the production runs used PM6/FF14SB MM potential and the AMBER package (AMBER 2018, University of California, San Francisco, CA 2018). The QM region composed of the ligand during the relaxation except for the last step where the C_β_Η_2_-OH segment of Ser460 and C_ε_H_2_-NH_2_ segment of Lys463 were also added to the QM region. This extended QM region was used also in the production runs. The link atom approach was used to separate the QM and MM subsystems. The relaxed structure was subjected to 11 ps QM/MM steered molecular dynamic (SMD) simulations with a spring constant of 50 kcal/(mol∙Å^2^) using the PLUMED^70^ patch to control the biasing potentials applied to the reaction coordinates during the simulations. A total of 48 structures evenly distributed in the path were extracted from the SMD simulation to use as starting structures for umbrella sampling windows. A total of 30 ps long QM/MM MD US simulations were performed in NVT ensemble with 50 kcal/(mol∙Å^2^) force constant in each window. The last 20 ps was used to produce the potential of mean force with WHAM (Grossfield, Alan, “WHAM: the weighted histogram analysis method” version 2.0.10, Rochester, New York, USA).

The non-covalent complex of lysine labelling **29** was obtained by docking it into the PDB structure 2Y2K after removal of the covalently bound ligand. Other steps were analogous to those described above for the serine labelling compounds. The QM region composed of the ligand, a water molecule, and the C_ε_H_2_-NH_2_ segment of Lys463.

## Supplementary Material

Supplemental Material
