# Supplementary material for: Boronic acid inhibitors of penicillin-binding protein 1b: serine and lysine labelling agents
Source: J Enzyme Inhib Med Chem. 2024 Feb 27;39(1):2305833. doi: 10.1080/14756366.2024.2305833 (PMC10901194; doi:10.1080/14756366.2024.2305833)
Supplement: Supplemental Material [file IENZ_A_2305833_SM5046.pdf]

SUPPORTING INFORMATION

Boronic Acid Inhibitors of Penicillin-Binding Protein 1b: Serine and Lysine Labelling Agents

Levente Kollár,[a,b] Katarina Grabrijan,[c] Martina Hrast,[c,] Krištof Bozovičar,[c] Tímea Imre,[d] György G. Ferenczy,[b] Stanislav Gobec,[c] György M. Keserű [a,b,]*

[a]L. Kollár, G. M. Keserű
Department of Organic Chemistry and Technology
Faculty of Chemical Technology and Biotechnology
Budapest University of Technology and Economics
Műegyetem rkp. 3., H-1111 Budapest, Hungary
E-mail: keseru.gyorgy@ttk.hu

[b]L. Kollár, G. G. Ferenczy, G. M. Keserű
Medicinal Chemistry Research Group
Research Centre for Natural Sciences
Magyar tudósok krt. 2, H-1117 Budapest, Hungary

[c]M. Hrast, K. Grabrijan, K. Bozovičar, S. Gobec
University of Ljubljana
Faculty of Pharmacy
Aškerčeva cesta 7, SI-1000, Ljubljana, Slovenia

[d]T. Imre
MS Metabolomics Research Group
Research Centre for Natural Sciences
Magyar tudósok krt 2, H-1117 Budapest, Hungary

Contents of Supporting Information

1. Detailed synthetic procedures and analytical properties of compounds 2

1.1. Synthesis of boroproline and boroglycine derivatives 2

1.2. Synthesis of branched boronic acid derivatives 4

1.3. The synthesis of the diazaborine-forming warhead-containing compounds. 11

2. Supplementary Tables and Figures 13

3. LC-MS chromatograms and spectra, ^{1}H and ^{13}C NMR spectra of compounds 21

References 50

1. Detailed synthetic procedures and analytical properties of compounds

1.1. Synthesis of boroproline and boroglycine derivatives

[1-(1-Methyl-1*H*-1,2,3-triazole-4-carbonyl)pyrrolidin-3-yl]boronic acid (6)

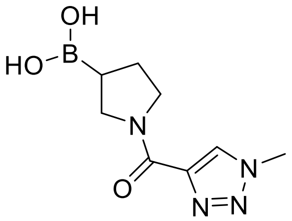

To a stirred solution of 1-methyl-1*H*-1,2,3-triazole-4-carboxylic acid (**10**, 25 mg, 0.20 mmol, 1.0 equiv.), HATU (84 mg, 0.22 mmol, 1.1 equiv.) and DIPEA (77 µL, 0.44 mmol, 2.2 equiv.) in DMF (1.0 mL), pyrrolidine-3-boronic acid pinacol ester hydrochloride (**11**, 47 mg, 0.20 mmol, 1.0 equiv.) was added and the mixture was allowed to stir at room temperature for 1 h. Diethanolamine (48 µL, 0.50 mmol, 2.5 equiv.) was added, and the mixture was allowed to stir at room temperature for 2 h. It was diluted with DCM and extracted with H_2O (3 × 10 mL). The aqueous layer was concentrated, and the crude residue was purified by reversed-phase flash column chromatography using eluents A (0.1% HCOOH in MeCN) and B (0.1% HCOOH in H_2O) (gradient from 1:9 to 10:0). The product [1-(1-methyl-1*H*-1,2,3-triazole-4-carbonyl)pyrrolidin-3-yl]boronic acid **(6)** was obtained as a white powder (18 mg, 40% yield). Mp.: 114–115 °C; ^{1}H NMR (500 MHz, CD_3OD) δ 8.31 (s, 1H), 4.14 (s, 3H), 3.87 – 3.71 (m, 3H), 3.46 (t, J = 11.2 Hz, 1H), 2.20 – 1.77 (m, 3H); ^{13}C NMR (75 MHz, CD_3OD) δ 161.54, 161.50 (duplication of signals), 145.06, 144.94 (duplication of signals), 129.61, 129.51 (duplication of signals), 52.61, 50.90, 50.68 (duplication of signals), 37.02, 29.97, 27.49; HRMS (ESI$^+$) *m/z* $[M+H]^+$, calcd. for $C_8H_{14}BN_4O_3$: 225.1153, found: 225.1155, Purity by HPLC: 98%.

1-Methyl-4-[(2*R*)-2-[(1*S*,2*S*,6*R*,8*S*)-2,9,9-trimethyl-3,5-dioxa-4-boratricyclo[6.1.1.0^{2,6}]decan-4-yl]pyrrolidine-1-carbonyl]-1*H*-1,2,3-triazole (13)

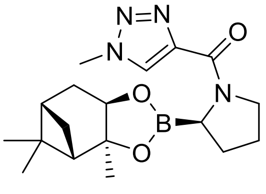

To a stirred solution of 1-methyl-1*H*-1,2,3-triazole-4-carboxylic acid (**10**, 51 mg, 0.40 mmol, 1.0 equiv.), HATU (167 mg, 0.44 mmol, 1.1 equiv.) and DIPEA (153 µL, 0.88 mmol, 2.2

equiv.) in DMF (1.0 mL), (*R*)-BoroPro-(+)-pinanediol hydrochloride (**12**, 114 mg, 0.40 mmol, 1.0 equiv.) was added and the mixture was allowed to stir at room temperature for 2 h. The mixture was purified by reversed-phase flash column chromatography using eluents A (0.1% HCOOH in MeCN) and B (0.1% HCOOH in H_2O) (gradient from 1:9 to 10:0). The product 1-methyl-4-[(2*R*)-2-[(1*S*,2*S*,6*R*,8*S*)-2,9,9-trimethyl-3,5-dioxa-4-boratricyclo[6.1.1.0^{2,6}]decan-4-yl]pyrrolidine-1-carbonyl]-1*H*-1,2,3-triazole **(13)** was obtained as a white powder (128 mg, 89% yield). Mp.: 82–84 °C; ^{1}H NMR (300 MHz, DMSO-d_6) δ 8.60 (d, *J* = 34.1 Hz, 1H), 4.12 (d, *J* = 19.3 Hz, 3H), 3.98 (d, *J* = 7.4 Hz, 1H), 3.39 – 3.26 (m, 1H), 2.33 – 1.57 (m, 10H), 1.45 (s, 2H), 1.37 – 1.17 (m, 6H), 0.80 (d, *J* = 2.8 Hz, 3H); HRMS (ESI$^+$) *m/z* [M+H]$^+$, calcd. for $C_{18}H_{28}BN_4O_3$: 359.2248, found: 359.2256; Purity by HPLC: 99%.

[(2*R*)-1-(1-Methyl-1*H*-1,2,3-triazole-4-carbonyl)pyrrolidin-2-yl]boronic acid (7)

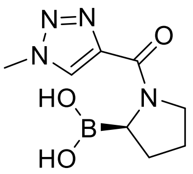

To a stirred solution of 1-methyl-4-[(2*R*)-2-[(1*S*,2*S*,6*R*,8*S*)-2,9,9-trimethyl-3,5-dioxa-4-boratricyclo[6.1.1.0^{2,6}]decan-4-yl]pyrrolidine-1-carbonyl]-1*H*-1,2,3-triazole (**13**, 47 mg, 0.13 mmol, 1.0 equiv.) in acetone (4 mL) and H_2O (6 mL), $NaIO_4$ (83 mg, 0.39 mmol, 3.0 equiv.) and NH_4OAc (24 mg, 0.31 mmol, 2.4 equiv.) were added and the mixture was allowed to stir at room temperature for 16 h. The mixture was concentrated and the crude residue was purified by reversed-phase flash column chromatography using eluents A (0.1% HCOOH in MeCN) and B (0.1% HCOOH in H_2O) (gradient from 1:9 to 10:0). The product [(2*R*)-1-(1-methyl-1*H*-1,2,3-triazole-4-carbonyl)pyrrolidin-2-yl]boronic acid **(7)** was obtained as a white powder (23 mg, 79% yield). Mp.: 159–160 °C; ^{1}H NMR (300 MHz, CD_3OD) δ 8.69 – 8.56 (m, 1H), 4.29 (d, *J* = 18.1 Hz, 3H), 3.78 – 3.62 (m, 1H), 3.51 – 3.36 (m, 1H), 3.19 – 3.06 (m, 1H), 2.21 – 2.00 (m, 2H), 1.98 – 1.73 (m, 2H); ^{13}C NMR (75 MHz, CD_3OD) δ 155.39, 140.33, 130.36, 47.13, 39.40, 29.09, 25.94; HRMS (ESI$^+$) *m/z* [M+H]$^+$, calcd. for $C_8H_{14}BN_4O_3$: 225.1153, found: 225.1152; Purity by HPLC: 99%.

[[(1-Methyl-1*H*-1,2,3-triazol-4-yl)formamido]methyl]boronic acid (8)

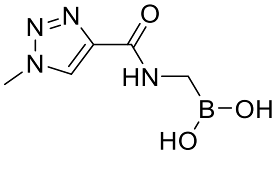

To a stirred solution of 1-methyl-1*H*-1,2,3-triazole-4-carboxylic acid (**10**, 25 mg, 0.20 mmol, 1.0 equiv.), HATU (84 mg, 0.22 mmol, 1.1 equiv.) and DIPEA (77 µL, 0.44 mmol, 2.2 equiv.) in DCM (3.0 mL), aminomethylboronic acid pinacol ester hydrochloride (**14**, 39 mg, 0.20 mmol, 1.0 equiv.) was added and the mixture was allowed to stir at room temperature for 1 h. The mixture was purified by normal-phase flash column chromatography using eluents DCM:MeOH (gradient from 10:0 to 1:1). The obtained viscous oil was triturated with Et_2O (8 mL) and concentrated. The product [[(1-methyl-1*H*-1,2,3-triazol-4-yl)formamido]methyl]boronic acid **(8)** was obtained as a white powder (22 mg, 60% yield). Mp.: 222 °C (decomposes); ^{1}H NMR (300 MHz, CD_3OD) δ 8.46 (s, 1H), 4.13 (s, 3H), 2.59 (s, 2H); ^{13}C NMR (126 MHz, CD_3OD) δ 129.32, 128.39, 111.39, 63.98, 37.25; HRMS (ESI$^+$) *m/z* $[M+H]^+$, calcd. for $C_5H_{10}BN_4O_3$: 185.0840, found: 185.0844, Purity by ^{1}H NMR: 96%.

[((2-Fluorophenyl)formamido)methyl]boronic acid (9)

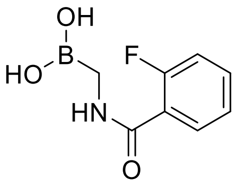

To a stirred solution of aminomethylboronic acid pinacol ester hydrochloride (**14**, 155 mg, 0.80 mmol, 1.0 equiv.) and DIPEA (307 µL, 1.76 mmol, 2.2 equiv.) in DCM (10.0 mL), 2-fluorobenzoyl chloride (**20**, 307 µL, 0.84 mmol) was added dropwise at room temperature. The resulting solution was allowed to stir for 1 h. The mixture was purified by normal-phase flash column chromatography using eluents DCM:MeOH (gradient from 10:0 to 1:1). The [((2-fluorophenyl)formamido)methyl]boronic acid (**9**) was obtained as a white powder (92 mg, 58% yield). Physical and spectroscopic data were identical to those reported previously.[1]

1.2. Synthesis of branched boronic acid derivatives

2-Fluorobenzamide (21)

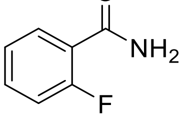

To a stirred aqueous solution of ammonia (25%, 40 mL) 2-fluorobenzoyl chloride (**20**, 3.58 mL, 30.0 mmol) was added dropwise at room temperature. Precipitation could be observed immediately, the resulting mixture was allowed to stir for 1 h. The precipitate was filtered and

washed with H_2O (3 × 40 mL). The product 2-fluorobenzamide **(21)** was obtained as a white powder (3,801 g, 91% yield). Mp.: 112–114 °C; ^{1}H NMR (500 MHz, DMSO-*d*$_6$) δ 7.67 (td, *J* = 7.5, 1.5 Hz, 2H), 7.60 (s, 1H), 7.56 – 7.48 (m, 1H), 7.26 (dd, *J* = 13.2, 4.9 Hz, 2H); ^{13}C NMR (126 MHz, DMSO-*d*$_6$) δ 165.20, 159.28 (d, *J* = 249.1 Hz), 132.42 (d, *J* = 8.6 Hz), 130.20 (d, *J* = 3.0 Hz), 124.35 (d, *J* = 3.4 Hz), 123.81 (d, *J* = 14.2 Hz), 116.04 (d, *J* = 22.7 Hz); HRMS (ESI^+) *m/z* $[M+H]^+$, calcd. for C_7H_7FNO: 140.0506, found: 140.0511, Purity by HPLC: 99%. Physical and spectroscopic data were identical to those reported previously.[2]

Ethyl (2*Z*)-3-[(2-fluorophenyl)formamido]prop-2-enoate (22)

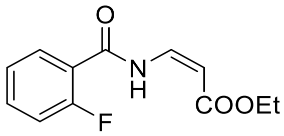

Preparation of **22** was done accordingly to Lopez et al.[3] A round-bottom flask was charged with 2-fluorobenzamide (**21**, 97 mg, 0.70 mmol, 1.0 equiv.), $Pd(OAc)_2$ (2 mg, 0.007 mmol, 0.01 equiv), trifluoroacetic acid (268 µL, 3.50 mmol, 5.0 equiv.), KOAc (137 mg, 1.40 mmol, 2.0 equiv.) and toluene (5.0 mL). The reaction mixture was stirred for 5 min under a nitrogen atmosphere at room temperature, then ethyl propiolate (107 µL, 1.05 mmol, 1.50 equiv.) was added dropwise. The reaction mixture was then stirred for 5 min, and then the mixture was heated to 70 °C and was allowed to stir for 4 h. The mixture was concentrated and the crude residue was purified by reversed-phase flash column chromatography using eluents A (0.1% HCOOH in MeCN) and B (0.1% HCOOH in H_2O) (gradient from 1:9 to 10:0). The product ethyl (2*Z*)-3-[(2-fluorophenyl)formamido]prop-2-enoate (**22**) was obtained as a light brown powder (100 mg, 60% yield). Mp.: 67–68 °C; ^{1}H NMR (500 MHz, DMSO-*d*$_6$) δ 11.42 (t, *J* = 10.6 Hz, 1H), 8.05 – 7.98 (m, 1H), 7.77 – 7.68 (m, 2H), 7.48 – 7.39 (m, 2H), 5.32 (d, *J* = 8.9 Hz, 1H), 4.17 (q, *J* = 7.1 Hz, 2H), 1.24 (t, *J* = 7.1 Hz, 3H); ^{13}C NMR (126 MHz, DMSO-*d*$_6$) δ 167.95, 160.58, 160.27 (d, *J* = 249.5 Hz), 137.78, 135.42 (d, *J* = 9.5 Hz), 131.69, 125.40 (d, *J* = 3.2 Hz), 119.11 (d, *J* = 11.0 Hz), 116.70 (d, *J* = 23.8 Hz), 97.71, 59.93, 14.03; HRMS (ESI^+) *m/z* $[M+H]^+$, calcd. for $C_{12}H_{13}FNO_3$: 238.0873, found: 238.0878, Purity by HPLC: 100%. Physical and spectroscopic data were identical to those reported previously.[3]

Ethyl 3-[(2-fluorophenyl)formamido]-3-(4,4,5,5-tetramethyl-1,3,2-dioxaborolan-2-yl)propanoate (16)

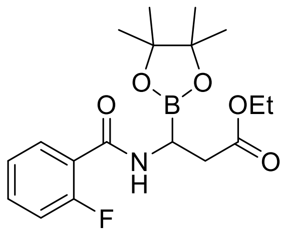

A flame-dried round-bottom flask was charged with CuCl (5 mg, 0.05 mmol, 0.1 equiv.) PPh_3 (14 mg, 0.055 mmol, 0.11 equiv.), KO^{t}Bu (56 mg, 0.5 mmol, 1.0 equiv), B_2pin_2 (140 mg, 0.55 mmol, 1.1 equiv), it was sealed with a septum, then THF (1.0 mL) was added and the solution was stirred for 0.5 h under a nitrogen atmosphere at room temperature. Ethyl (2*Z*)-3-[(2-fluorophenyl)formamido]prop-2-enoate (**22**, 119 mg, 0.5 mmol, 1.0 equiv.) in THF (1.0 mL) was added dropwise, followed by methanol (81 μL, 2.0 mmol, 4.0 equiv). Then, the reaction mixture was stirred at RT for 1 h. It was purified by normal-phase flash column chromatography using eluents hexane and EtOAc (gradient from 0:100 to 1:1). The product ethyl 3-[(2-fluorophenyl)formamido]-3-(4,4,5,5-tetramethyl-1,3,2-dioxaborolan-2-yl)propanoate (**16**) was obtained as a white solid (92 mg, 50% yield). Mp.: 74–75 °C; ^{1}H NMR (300 MHz, $CDCl_3$) δ 8.61 (d, *J* = 8.9 Hz, 1H), 8.07 (t, *J* = 7.4 Hz, 1H), 7.54 (dd, *J* = 13.0, 6.5 Hz, 1H), 7.25 (t, *J* = 7.5 Hz, 1H), 7.18 – 7.07 (m, 1H), 4.14 (dd, *J* = 12.4, 6.1 Hz, 2H), 3.10 (d, *J* = 8.0 Hz, 1H), 2.58 (d, *J* = 10.2 Hz, 2H), 1.28 – 1.17 (m, 15H); ^{13}C NMR (75 MHz, $CDCl_3$) δ 174.91, 167.72 (d, *J* = 3.0 Hz), 161.67 (d, *J* = 253.4 Hz), 135.69 (d, *J* = 9.8 Hz), 132.67, 125.07 (d, *J* = 3.3 Hz), 116.34 (d, *J* = 22.8 Hz), 114.77 (d, *J* = 9.1 Hz), 80.82, 60.71, 35.15, 25.28, 24.93, 24.51, 14.24; HRMS (ESI$^+$) *m/z* [M+H]$^+$, calcd. for $C_{18}H_{26}BNO_5F$: 366.1888, found: 366.1895; Purity by HPLC: 97%.

2-Fluoro-*N*-(2-hydroxy-5-oxo-1,2-oxaborolan-3-yl)benzamide (15)

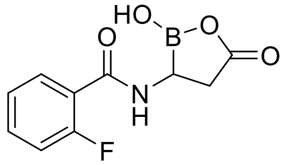

To a stirred aqueous solution of 3-[(2-fluorophenyl)formamido]-3-(4,4,5,5-tetramethyl-1,3,2-dioxaborolan-2-yl)propanoate (**16**, 38 mg, 0,105 mmol, 1.0 equiv.) in MeCN (2.0 mL), 10% NaOH (2.0 mL) was added and the solution was allowed to stir for 30 min. It was purified by normal-phase flash column chromatography using eluents hexane and EtOAc (gradient from 0:100 to 100:0). The product 2-fluoro-*N*-(2-hydroxy-5-oxo-1,2-oxaborolan-3-yl)benzamide (**15**) was obtained as a colorless oil (22 mg, 87% yield). ^{1}H NMR (300 MHz, DMSO-d_6 + D_2O) δ 10.96 (s, 1H), 7.94 (t, *J* = 6.9 Hz, 1H), 7.79 (dd, *J* = 13.1, 6.6 Hz, 1H), 7.45 (dd, *J* = 19.0, 9.9

Hz, 2H), 2.97 (d, *J* = 7.3 Hz, 1H), 2.72 (dd, *J* = 18.4, 8.1 Hz, 1H), 2.33 (d, *J* = 18.5 Hz, 1H); ^{13}C NMR (75 MHz, DMSO-d_6 + D_2O) δ 177.58, 168.96, 160.64 (d, *J* = 257.1 Hz), 136.98 (d, *J* = 9.0 Hz), 131.35, 125.49 (d, *J* = 2.8 Hz), 117.16 (d, *J* = 21.3 Hz), 38.09; HRMS (ESI$^+$) *m/z* [M+H]$^+$, calcd. for $C_{10}H_{10}BNO_4F$: 238.0686, found: 238.0693; Purity by HPLC: 99%.

(2*Z*)-3-[(2-fluorophenyl)formamido]prop-2-enoic acid (23)

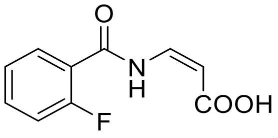

To a solution of ethyl (2*Z*)-3-[(2-fluorophenyl)formamido]prop-2-enoate (**22**, 237 mg, 1.00 mmol, 1.0 equiv.) in DMF (4 mL) and H_2O (2 mL) K_2CO_3 (276 mg, 2.00 mmol, 2.0 equiv.) was added and the mixture was heated to 50 °C, it was allowed to stir for 16 h. It was diluted with EtOAc (30 mL) and extracted with water (30 mL). The aqueous layer was acidified to pH 1 with 1 M HCl and extracted with EtOAc (3 × 30 mL). It was dried over Na_2SO_4, filtered, and concentrated. The product (2*Z*)-3-[(2-fluorophenyl)formamido]prop-2-enoic acid (**23**) was obtained as a brown solid (158 mg, 76% yield). Mp.: 160–162 °C; ^{1}H NMR (500 MHz, DMSO-d_6) δ 11.62 (t, *J* = 10.0 Hz, 1H), 8.01 (td, *J* = 7.8, 1.6 Hz, 1H), 7.74 – 7.65 (m, 2H), 7.47 – 7.39 (m, 2H), 5.26 (d, *J* = 8.9 Hz, 1H); ^{13}C NMR (126 MHz, DMSO-d_6) δ 169.57, 160.54, 160.21 (d, *J* = 249.8 Hz), 137.33, 135.28 (d, *J* = 9.5 Hz), 131.65, 125.37 (d, *J* = 3.3 Hz), 119.35 (d, *J* = 11.2 Hz), 116.71 (d, *J* = 23.6 Hz), 98.94; HRMS (ESI$^-$) *m/z* [M–H]$^-$, calcd. for $C_{10}H_7FNO_3$: 208.0404, found: 208.0401, Purity by HPLC: 99%.

(2*Z*)-3-[(2-fluorophenyl)formamido]-*N*-phenylprop-2-enamide (24)

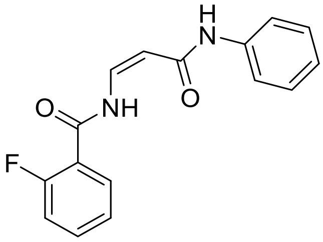

To a stirred solution of (2*Z*)-3-[(2-fluorophenyl)formamido]prop-2-enoic acid (**23**, 446 mg, 2.13 mmol, 1.0 equiv.)**,** HATU (891 mg, 2.34 mmol, 1.1 equiv.) and DIPEA (742 µL, 4.26 mmol, 2.0 equiv.) in DMF (4.0 mL), aniline (194 µL, 0.40 mmol, 1.0 equiv.) was added and the mixture was allowed to stir at 50 °C for 16 h. The mixture was purified by reversed-phase flash column chromatography using eluents A (0.1% HCOOH in MeCN) and B (0.1% HCOOH in H_2O) (gradient from 1:9 to 10:0). The product (2*Z*)-3-[(2-fluorophenyl)formamido]-*N*-

phenylprop-2-enamide (**24**) was obtained as a brown powder (273 mg, 45% yield). Mp.: 154–156 °C; ^{1}H NMR (500 MHz, DMSO-*d$_6$*) δ 12.25 (t, *J* = 9.9 Hz, 1H), 10.19 (s, 1H), 8.01 (dd, *J* = 11.4, 4.0 Hz, 1H), 7.72 (dd, *J* = 13.7, 5.5 Hz, 1H), 7.64 (d, *J* = 7.6 Hz, 3H), 7.49 – 7.39 (m, 2H), 7.33 (t, *J* = 7.9 Hz, 2H), 7.07 (t, *J* = 7.4 Hz, 1H), 5.56 (d, *J* = 8.9 Hz, 1H); ^{13}C NMR (126 MHz, DMSO-*d$_6$*) δ 166.99, 160.93, 139.21, 135.57 (d, *J* = 9.1 Hz), 135.41, 132.05, 129.26, 125.82 (d, *J* = 3.2 Hz), 123.97, 119.85, 117.23 (d, *J* = 23.2 Hz), 102.26; HRMS (ESI$^+$) *m/z* [M+H]$^+$, calcd. for $C_{16}H_{14}FN_2O_2$: 285.1033, found: 285.1037, Purity by HPLC: 100%.

3-[(2-Fluorophenyl)formamido]-*N*-phenyl-3-(4,4,5,5-tetramethyl-1,3,2-dioxaborolan-2-yl)propanamide (17)

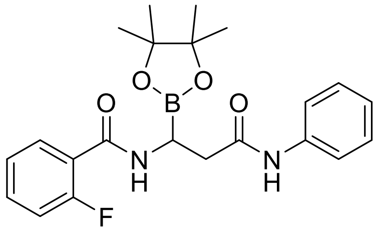

A flame-dried round-bottom flask was charged with CuCl (2 mg, 0.02 mmol, 0.1 equiv.) *rac*-BINAP (14 mg, 0.022 mmol, 0.11 equiv.), KO^{t}Bu (22 mg, 0.2 mmol, 1.0 equiv), B_2pin_2 (56 mg, 0.22 mmol, 1.1 equiv), it was sealed with a septum, then THF (1.0 mL) was added and the solution was stirred for 0.5 h under a nitrogen atmosphere at room temperature. (2*Z*)-3-[(2-fluorophenyl)formamido]-*N*-phenylprop-2-enamide (**19**, 57 mg, 0.2 mmol, 1.0 equiv.) in THF (1.0 mL) was added dropwise, followed by methanol (32 µL, 2.0 mmol, 4.0 equiv). Then, the reaction mixture was stirred at 60 °C for 3 h. It was purified by normal-phase flash column chromatography using eluents hexane and EtOAc (gradient from 0:100 to 1:1). The product 3-[(2-fluorophenyl)formamido]-*N*-phenyl-3-(4,4,5,5-tetramethyl-1,3,2-dioxaborolan-2-yl)propanamide (**14**) was obtained as colorless oil (53 mg, 64% yield). ^{1}H NMR (300 MHz, $CDCl_3$) δ 9.10 (s, 1H), 8.89 (s, 1H), 8.02 (td, *J* = 7.7, 1.6 Hz, 1H), 7.58 – 7.47 (m, 3H), 7.27 – 7.22 (m, 3H), 7.17 – 7.02 (m, 2H), 3.25 (t, *J* = 5.7 Hz, 1H), 2.86 – 2.69 (m, 2H), 1.23 (s, 12H); ^{13}C NMR (75 MHz, $CDCl_3$) δ 171.66 (d, *J* = 1.6 Hz), 168.06 (d, *J* = 2.4 Hz), 161.55 (d, *J* = 254.9 Hz), 138.11, 135.74 (d, *J* = 9.9 Hz), 132.29, 128.78, 124.90 (d, *J* = 3.3 Hz), 124.00, 119.98, 116.39 (d, *J* = 22.7 Hz), 114.28, 82.86, 80.87, 38.14, 24.50; HRMS (ESI$^+$) *m/z* [M+H]$^+$, calcd. for $C_{22}H_{27}BN_2O_4F$: 413.2047, found: 413.2052; Purity by HPLC: 100%.

***N,N*-dibenzylprop-2-ynamide (27)**

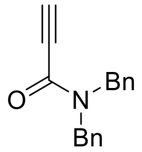

Preparation of **27** was done accordingly to Lopez et al.[3] A solution of propiolic acid (**25**, 308 µL, 5.0 mmol, 1.0 equiv.) in DCM (20 mL) was cooled at 0 °C. DCC (1032 mg, 5.0 mmol, 1.0 equiv.) was added in one portion, then *N*,*N*-dibenzylamine (**26**, 961 µL, 5.0 mmol, 1.0 equiv) was added dropwise and the mixture was allowed to warm up to room temperature and it was allowed to stir for 16 h. The mixture was filtered, concentrated and the residue was purified by reversed-phase flash column chromatography using eluents A (0.1% HCOOH in MeCN) and B (0.1% HCOOH in H_2O) (gradient from 1:9 to 10:0). *N*,*N*-dibenzylprop-2-ynamide (**27**) was obtained as a yellow oil (1030 mg, 83% yield). ^{1}H NMR (500 MHz, DMSO-d_6) δ 7.39 (t, J = 7.3 Hz, 2H), 7.36 – 7.30 (m, 3H), 7.28 (t, J = 7.3 Hz, 3H), 7.19 (d, J = 7.1 Hz, 2H), 4.72 (s, 2H), 4.60 (s, 1H), 4.44 (s, 2H); ^{13}C NMR (126 MHz, DMSO-d_6) δ 153.17, 136.23, 136.18, 128.71, 128.50, 127.72, 127.66, 127.34, 127.24, 82.58, 75.89, 51.24, 46.59; HRMS (ESI$^+$) *m/z* [M+H]$^+$, calcd. for $C_{17}H_{16}NO$: 250.1226, found: 250.1223; Purity by HPLC: 99%. Physical and spectroscopic data were identical to those reported previously.[3]

(2*Z*)-*N*,*N*-dibenzyl-3-[(2-fluorophenyl)formamido]prop-2-enamide (28)

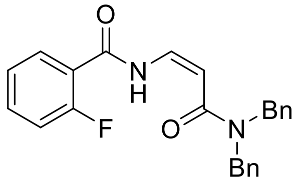

A round-bottom flask was charged with 2-fluorobenzamide (**21**, 139 mg, 1.00 mmol, 1.0 equiv.), $Pd(OAc)_2$ (2 mg, 0.01 mmol, 0.01 equiv), trifluoroacetic acid (383 µL, 5.00 mmol, 5.0 equiv.), KOAc (196 mg, 2.00 mmol, 2.0 equiv.) and toluene (10.0 mL). The reaction mixture was stirred for 5 min under a nitrogen atmosphere at room temperature, *N*,*N*-dibenzylprop-2-ynamide (**27**, 374 mg, 1.50 mmol, 1.5 equiv.) was added. The reaction mixture was then stirred for 5 min, and then the mixture was heated to 90 °C and it was allowed to stir for 16 h. The mixture was concentrated, and the crude residue was purified by reversed-phase flash column chromatography using eluents A (0.1% HCOOH in MeCN) and B (0.1% HCOOH in H_2O) (gradient from 1:9 to 10:0). The product (2*Z*)-*N*,*N*-dibenzyl-3-[(2-fluorophenyl)formamido]prop-2-enamide (**28**) was obtained as a yellow solid (174 mg, 45% yield). Mp.: 289–292 °C (decomposes); ^{1}H NMR (500 MHz, DMSO-d_6) δ 12.51 (t, J = 9.4 Hz, 1H), 8.03 – 7.97 (m, 1H), 7.70 (ddd, J = 9.4, 7.6, 1.8 Hz, 1H), 7.60 (t, J = 9.7 Hz, 1H), 7.43 –

7.20 (m, 13H), 5.80 (d, *J* = 9.1 Hz, 1H), 4.64 (s, 2H), 4.61 (s, 2H); ^{13}C NMR (126 MHz, DMSO-d_6) δ 168.38, 160.14 (d, *J* = 249.7 Hz), 137.46, 137.23, 135.86, 135.04 (d, *J* = 9.6 Hz), 131.50, 128.64, 128.44, 127.65, 127.26, 127.10, 126.51, 125.28 (d, *J* = 3.2 Hz), 119.74 (d, *J* = 11.4 Hz), 109.47, 97.16, 50.17, 48.25; HRMS (ESI$^+$) *m/z* [M+H]$^+$, calcd. for $C_{24}H_{22}FN_2O_2$: 389.1659, found: 389.1653; Purity by HPLC: 98%.

***N,N*-dibenzyl-3-[(2-fluorophenyl)formamido]-3-(4,4,5,5-tetramethyl-1,3,2-dioxaborolan-2-yl)propanamide (18)**

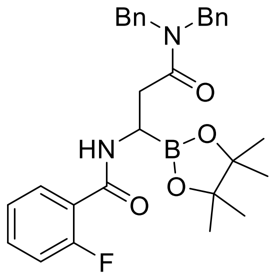

A flame-dried round-bottom flask was charged with CuCl (1 mg, 0.013 mmol, 0.1 equiv.) *rac*-BINAP (9 mg, 0.014 mmol, 0.11 equiv.), KO^{t}Bu (15 mg, 0.13 mmol, 1.0 equiv), B_2pin_2 (36 mg, 0.14 mmol, 1.1 equiv), it was sealed with a septum, then THF (1.0 mL) was added and the solution was stirred for 0.5 h under a nitrogen atmosphere at room temperature. (2*Z*)-*N*,*N*-dibenzyl-3-[(2-fluorophenyl)formamido]prop-2-enamide (**28**, 50 mg, 0.13 mmol, 1.0 equiv.) in THF (1.0 mL) was added dropwise, followed by methanol (21 µL, 0,52 mmol, 4.0 equiv). Then, the reaction mixture was stirred at room temperature for 16 h. It was purified by reversed-phase flash column chromatography using eluents A (0.1% HCOOH in MeCN) and B (0.1% HCOOH in H_2O) (gradient from 1:9 to 10:0). The product *N,N*-dibenzyl-3-[(2-fluorophenyl)formamido]-3-(4,4,5,5-tetramethyl-1,3,2-dioxaborolan-2-yl)propanamide (**18**) was obtained as a colorless oil (16 mg, 23% yield). ^{1}H NMR (300 MHz, $CDCl_3$) δ 8.59 (d, *J* = 9.4 Hz, 1H), 8.07 (t, *J* = 7.7 Hz, 1H), 7.53 (dd, *J* = 13.2, 7.3 Hz, 1H), 7.39 – 7.07 (m, 12H), 4.62 (s, 2H), 4.44 (s, 2H), 3.41 – 3.31 (m, 1H), 3.02 – 2.80 (m, 2H), 1.23 (s, 12H); ^{13}C NMR (75 MHz, $CDCl_3$) δ 176.06, 166.31 (d, *J* = 3.0 Hz), 161.30 (d, *J* = 252.2 Hz), 136.21, 135.24, 134.49, 132.18 (d, *J* = 1.4 Hz), 129.01, 128.74, 128.32, 127.84 (d, *J* = 16.8 Hz), 126.77, 124.69 (d, *J* = 3.3 Hz), 116.35, 116.05, 80.80, 50.18, 48.80, 35.62, 25.06 (d, *J* = 16.4 Hz); HRMS (ESI$^-$) *m/z* [M–H]$^-$, calcd. for $C_{30}H_{35}BN_2O_4F$: 517.2668, found: 517.2651; Purity by HPLC: 99%.

[2-(Bibenzylcarbamoyl)-1-[(2-fluorophenyl)formamido]ethyl]boronic acid (19)

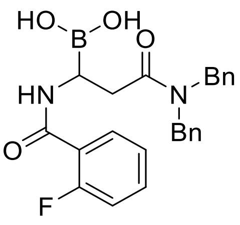

To a stirred solution of *N,N*-dibenzyl-3-[(2-fluorophenyl)formamido]-3-(4,4,5,5-tetramethyl-1,3,2-dioxaborolan-2-yl)propanamide (**18**, 14 mg, 0.027 mmol, 1.0 equiv.) and isobutylboronic acid (20 mg, 0.20 mmol, 7.4 equiv.) in MeOH (1.4 mL) and 1 M HCl (0.8 mL) *n*-hexane (1.4 mL) was added and the biphasic mixture was stirred vigorously for 16 h. The *n*-hexane layer was discarded, the MeOH layer was washed again with *n*-hexane (3 × 1 mL), and concentrated. The residue was re-dissolved in diisopropyl ether (1.8 mL), and sonicated for 15 min, followed by decanting and concentrating the ether. The residue was redissolved in MeCN (1.5 mL), sonicated for 15 min, decanted, and the MeCN solution was concentrated. The product [2-(dibenzylcarbamoyl)-1-[(2-fluorophenyl)formamido]ethyl]boronic acid (**19**) was obtained as a colorless oil (6 mg, 52% yield). ^{1}H NMR (300 MHz, CD_3OD) δ 8.05 (dd, *J* = 10.7, 4.5 Hz, 1H), 7.83 – 7.74 (m, 1H), 7.45 – 7.15 (m, 13H), 4.91 – 4.82 (m, 1H), 4.74 – 4.68 (m, 2H), 4.60 (d, *J* = 14.7 Hz, 1H), 3.52 (d, *J* = 2.9 Hz, 1H), 3.22 (dd, *J* = 3.1, 1.5 Hz, 2H); ^{13}C NMR (75 MHz, CD_3OD) δ 183.41, 172.51, 163.60 (d, *J* = 257.6 Hz), 139.51 (d, *J* = 9.9 Hz), 134.88, 134.14, 133.42, 130.64, 130.37, 130.26, 130.07, 129.44, 127.01 (d, *J* = 3.5 Hz), 118.53, 118.24, 114.04 (d, *J* = 9.9 Hz), 55.02, 54.15, 39.21; HRMS (ESI$^-$) *m/z* [M–H]$^-$, calcd. for $C_{24}H_{23}BN_2O_4F$: 433.1734, found: 433.1731; Purity by HPLC: 99%.

1.3. The synthesis of the diazaborine-forming warhead-containing compounds.

2-[[[2-(4,4,5,5-tetramethyl-1,3,2-dioxaborolan-2-yl)phenyl]methyl]amino]benzaldehyde (32)

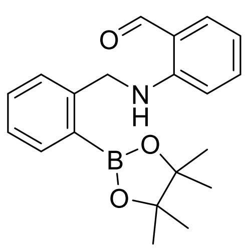

2-Aminobenzaldehyde (**34**, 127 mg, 1.05 mmol, 3.0 equiv.) was dissolved in dry acetonitrile (5 mL). Cs_2CO_3 (137 mg, 0.42 mmol, 1.2 equiv.) and 2-bromomethylphenylboronic acid pinacol ester (**35**, 104 mg, 0.35 mmol, 1.0 equiv.) were added, then the mixture was stirred at 80 °C for 16 h. It was purified by reversed-phase flash column chromatography using eluents

A (0.1% HCOOH in MeCN) and B (0.1% HCOOH in H_2O) (gradient from 1:9 to 10:0). The product 2-[[[2-(4,4,5,5-tetramethyl-1,3,2-dioxaborolan-2-yl)phenyl]methyl]amino]benzaldehyde (**32**) was obtained as yellow oil (65 mg, 55% yield). ^{1}H NMR (500 MHz, DMSO-d_6) δ 9.80 (s, 1H), 8.68 (t, *J* = 5.9 Hz, 1H), 7.72 (d, *J* = 7.2 Hz, 1H), 7.58 (dd, *J* = 7.7, 1.5 Hz, 1H), 7.45 – 7.41 (m, 2H), 7.41 – 7.36 (m, 1H), 7.28 (ddd, *J* = 7.5, 5.4, 3.3 Hz, 1H), 6.85 (d, *J* = 8.6 Hz, 1H), 6.67 (dd, *J* = 10.9, 3.9 Hz, 1H), 4.64 (d, *J* = 6.1 Hz, 2H), 1.29 (s, 12H); ^{13}C NMR (126 MHz, DMSO-d_6) δ 194.04, 149.78, 144.80, 136.71, 136.03, 135.69, 131.10, 128.21, 126.56, 118.13, 114.73, 111.17, 109.46, 83.62, 45.55, 24.49; HRMS (ESI$^+$) *m/z* [M+H]$^+$, calcd. for $C_{20}H_{25}BNO_3$: 338.1922, found: 338.1940; Purity by HPLC: 100%. Physical and spectroscopic data were identical to those reported previously.[4]

[2-[[(2-formylphenyl)amino]methyl]phenyl]boronic acid (33)

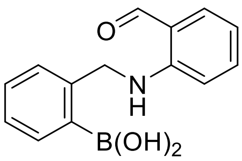

To a stirred solution of 2-[[[2-(4,4,5,5-tetramethyl-1,3,2-dioxaborolan-2 yl)phenyl]methyl]amino]benzaldehyde (**32**, 45 mg, 0.13 mmol, 1.0 equiv.) in acetone (3 mL) and H_2O (2 mL), $NaIO_4$ (85 mg, 0.39 mmol, 3.0 equiv.) and NH_4OAc (24 mg, 0.31 mmol, 2.4 equiv.) were added and the mixture was allowed to stir at room temperature for 16 h. The mixture was then diluted with H_2O (10 mL), pH was adjusted to 3 with 1 M HCl, and it was extracted with EtOAc (3 × 20 mL). The combined organic layers were removed under reduced pressure and the crude residue was purified by reversed-phase flash column chromatography using eluents A (0.1% HCOOH in MeCN) and B (0.1% HCOOH in H_2O) (gradient from 1:9 to 10:0)**.** The product [2-[[(2-formylphenyl)amino]methyl]phenyl]boronic acid (**33**) was obtained as a yellow oil (14 mg, 41% yield). Physical and spectroscopic data were identical to those reported previously.[4]

2. Supplementary Tables and Figures

Table S1. Structures and inhibitory activities of 26 boronic acids selected by virtual screening (**1-2**, **VS1-VS16**) or from an in-house collection (**MCRG1-MCRG8**).

**Compound**	**Structure**	**RA%@500µM PBP1b *S.pneumoniae,* 60 min preincubation**	**RA%@50µM PBP1b *S.pneumoniae,* 60 min preincubation**
**1**	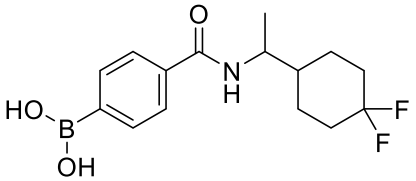	38.6 ± 0.2	78.8 ± 2.7
**2**	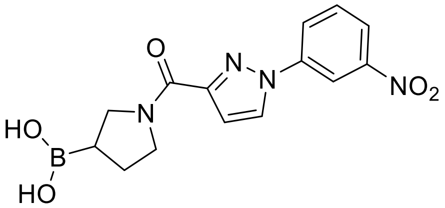	42.1 ± 0.6	83.5 ± 0.1
**VS1**	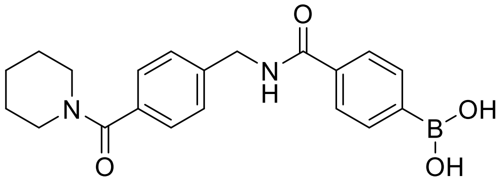	56.? ± ?	86.7 ± 0.6
**VS2**	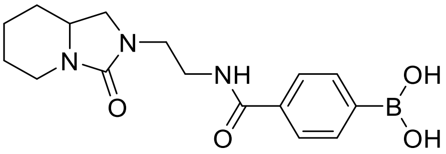	91.4± 1.1	96.1 ± 0.2
**VS3**	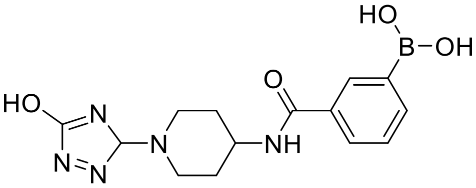	90.9 ± 2.2	97.6 ± 0.1

**VS4**	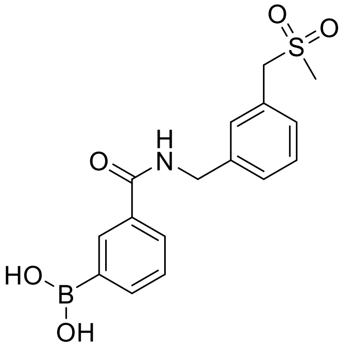	90.1 ± 2.4	94.6 ± 5.0
**VS5**	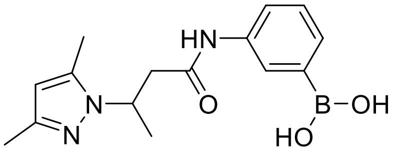	91.5 ± 1.7	82.5 ± 18.6
**VS6**	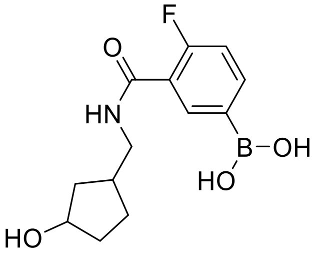	94.6 ± 2.4	86.6 ± 14.7
**VS7**	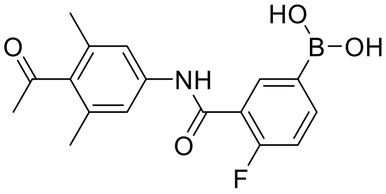	85.0 ± 2.5	89.6 ± 10.2
**VS8**	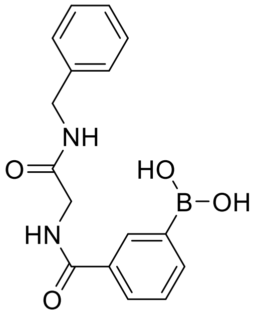	85.5 ± 2.3	99.8 ± 2.2
**VS9**	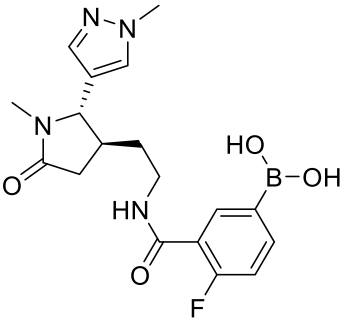	89.1 ± 3.9	95.8 ± 0.8

**VS10**	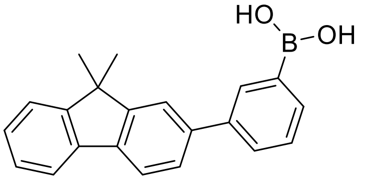	73.9 ± 5.1	97.0 ± 0.1
**VS11**	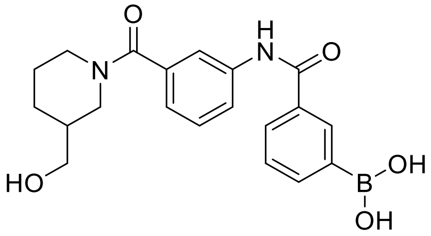	90.4 ± 0.4	96.6 ± 0.9
**VS12**	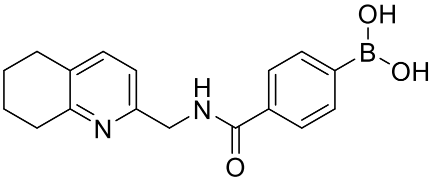	57.0 ± 5.3	90.4 ± 1.0
**VS13**	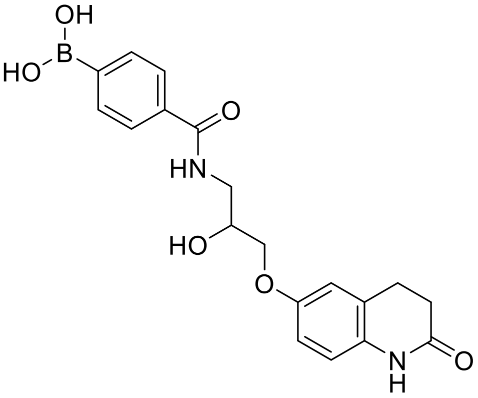	52.7 ± 1.3	86.2 ± 0.4
**VS14**	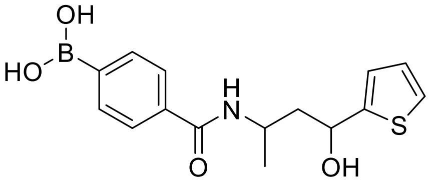	50.1 ± 0.6	91.8 ± 2.8
**VS15**	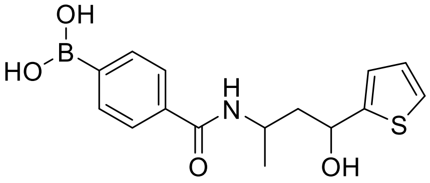	66.6 ± 0.4	99.0 ± 4.0
**VS16**	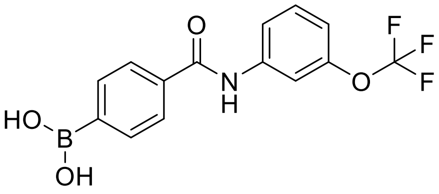	83.8 ± 0.3	102.2 ± 3.5

**MCGR1**	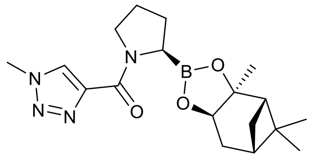	99.1 ± 2.0	104.1 ± 2.2
**MCRG2**	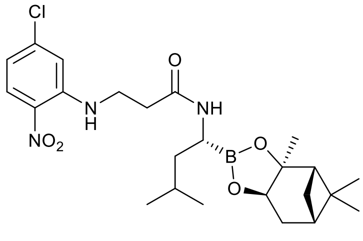	86.7± 3.8	96.0 ± 3.8
**MCRG3**	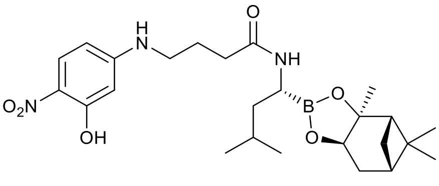	86.9 ± 6.6	98.9 ± 0.4
**MCRG4**	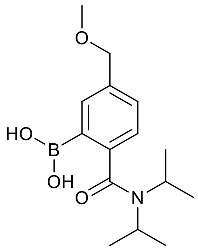	97.8 ± 2.4	98.4 ± 1.6
**MCRG5**	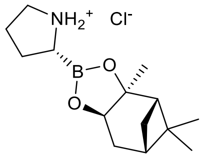	97.9 ± 4.1	99.8 ± 0.2
**MCRG6**	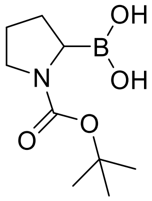	79.3 ± 3.1	96.8 ± 0.6
**MCRG7**	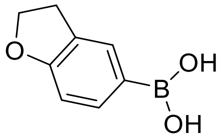	96.9 ± 2.0	97.8 ± 0.7
**MCRG8**	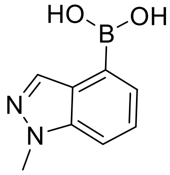	56.3 ± 1.5	89.1 ± 1.9

Figure S1. Schematic free energy profile of the two-step covalent inhibition process (adapted from ref [5]).

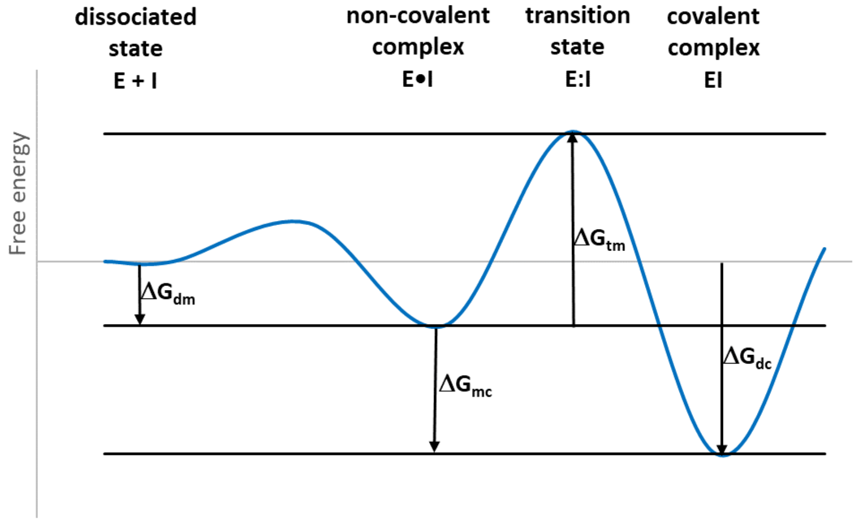

Figure S2. Results of swarms-of-trajectories string method calculations for the Ser460 activation and nucleophilic attack. 8 iterations were performed. At higher iteration numbers the B-O bond formation is fairly advanced when the proton transfer occurs. See Scheme 6 in the main text for the reaction.

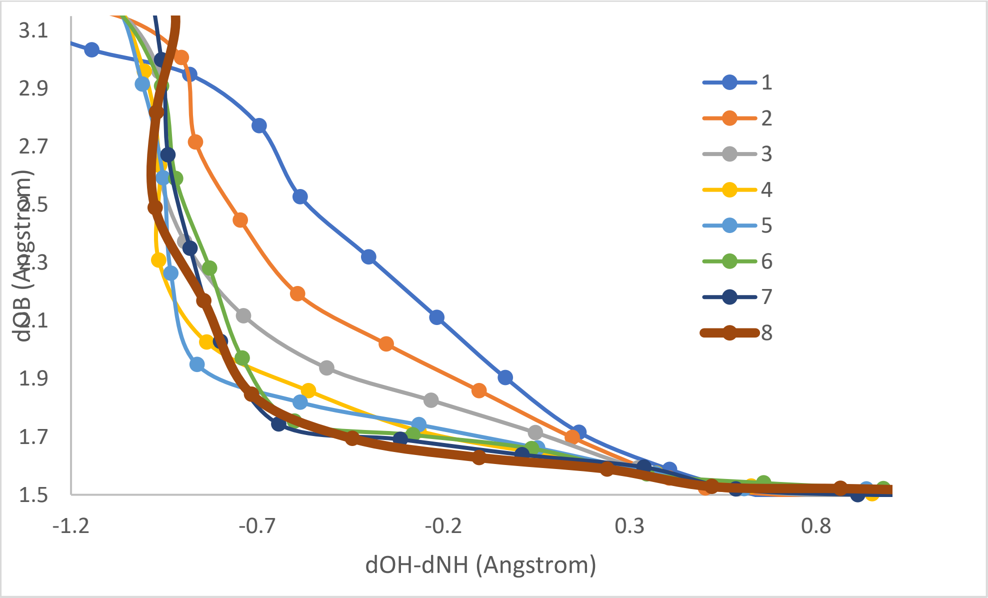

Figure S3. Computed free energy profile for the reaction between PBP1b and compounds of Table 4 in the main text. Statistical errors are represented with error bars.

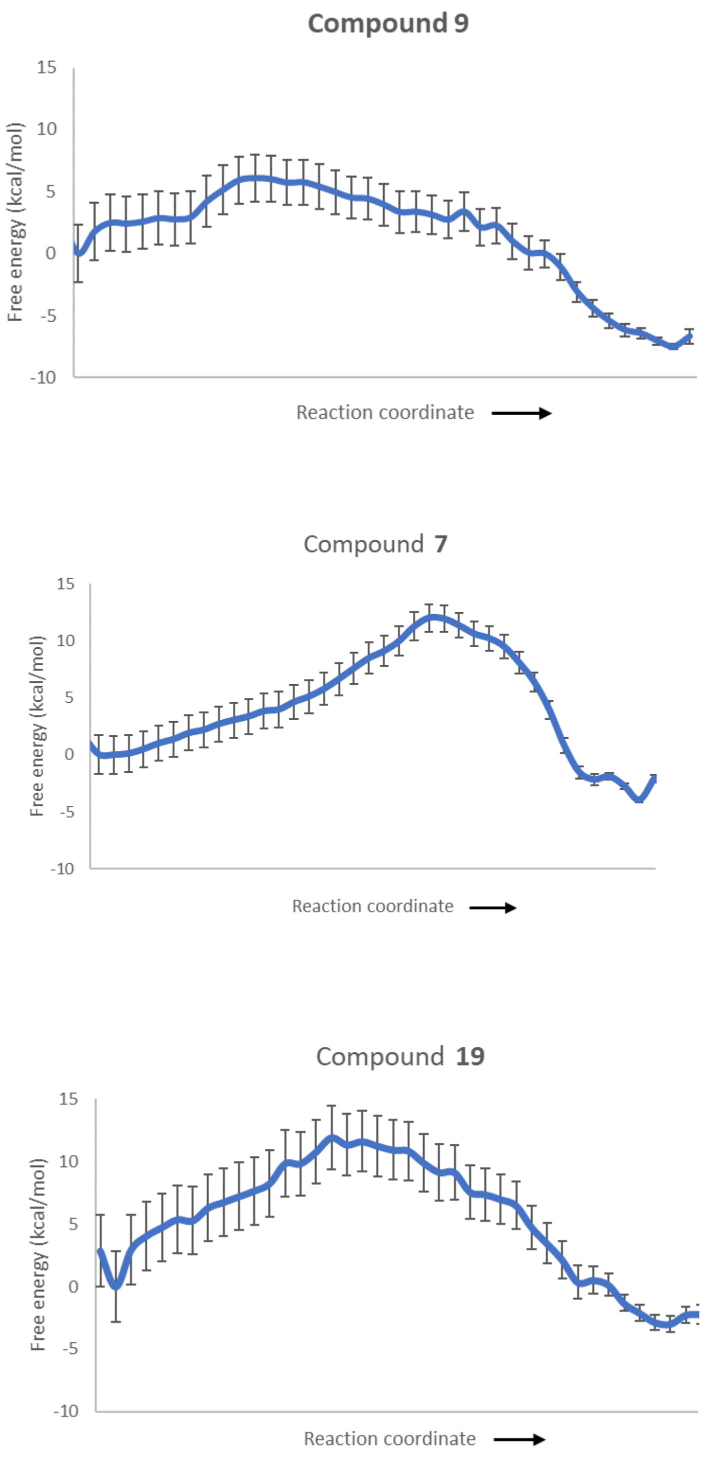

Figure S4. a) The *N*-ethyl derivatives of compounds **29** and **33**. The *N*-ethyl derivatives were non-covalently docked into the K463A mutant of PBP1b to study the potential binding modes of **29** and **33**. **b)** Docking pose of the N-ethyl derivative of **29** into PBP1b K463A. The catalityic dyad is shown with ball-and-thick, while other residues in thin.ttube representation. Luigand is shown with thick tubes. **c)** 2D interaction diagram of the docking pose of the N-ethyl derivative of **29** into PBP1b K463A.

a)

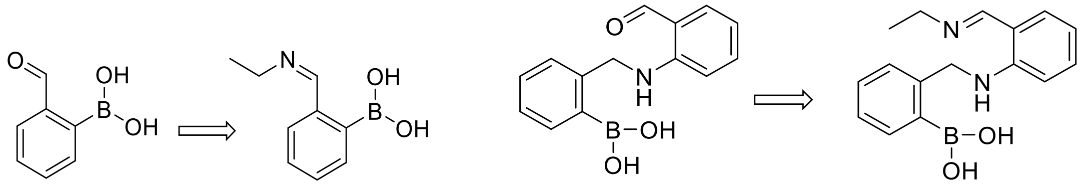

29 **33**

b)

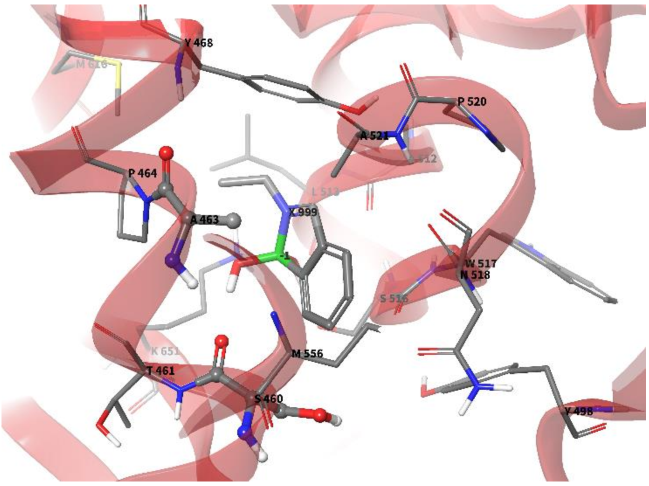

c)

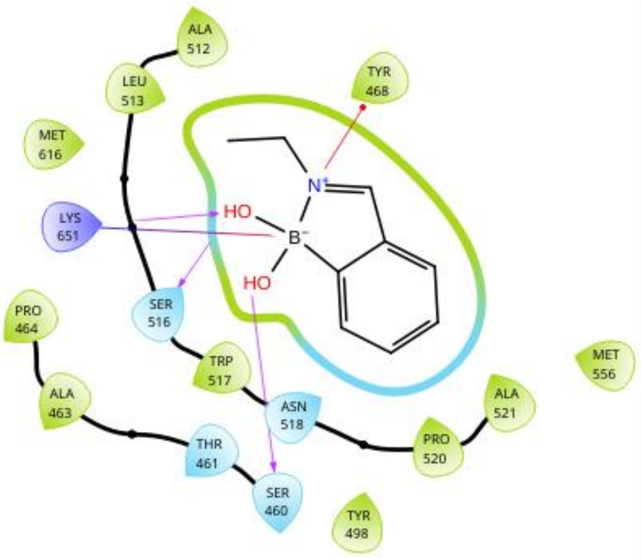

Figure S5. Computed free energy profile for the reaction between PBP1b and compound **29**. Statistical errors are represented with error bars.

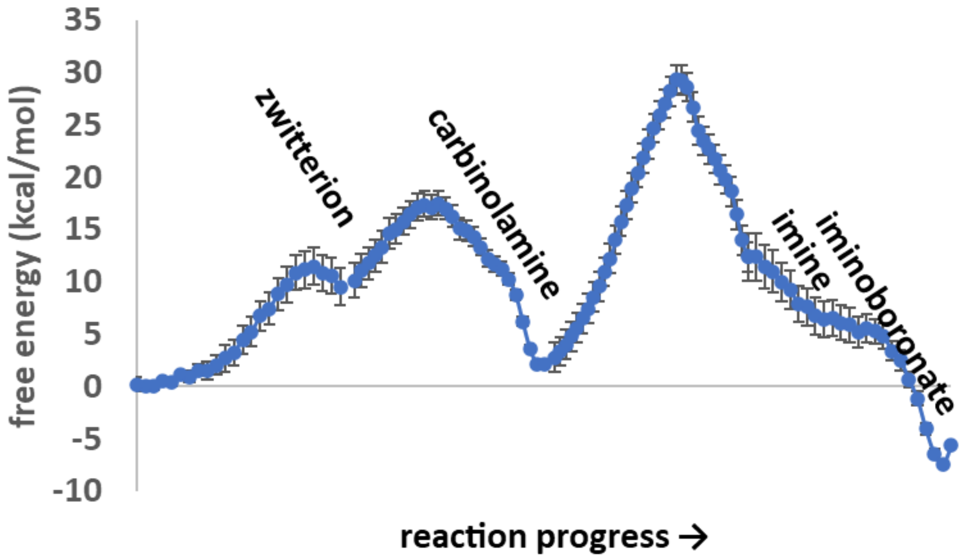

Figure S6. Deconvoluted mass spectrum of reference PBP1b (blue) and **29** (132/114 Da) modified PBP1b (magenta).

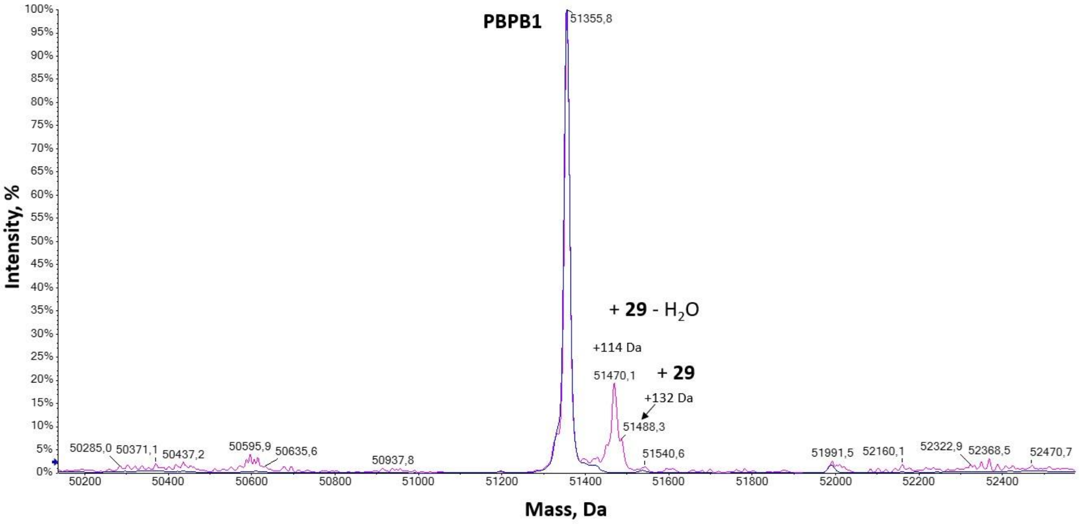

Figure S7. Deconvoluted mass spectrum of reference PBP1b (blue) and **30** (200/182 Da) modified PBP1b (magenta).

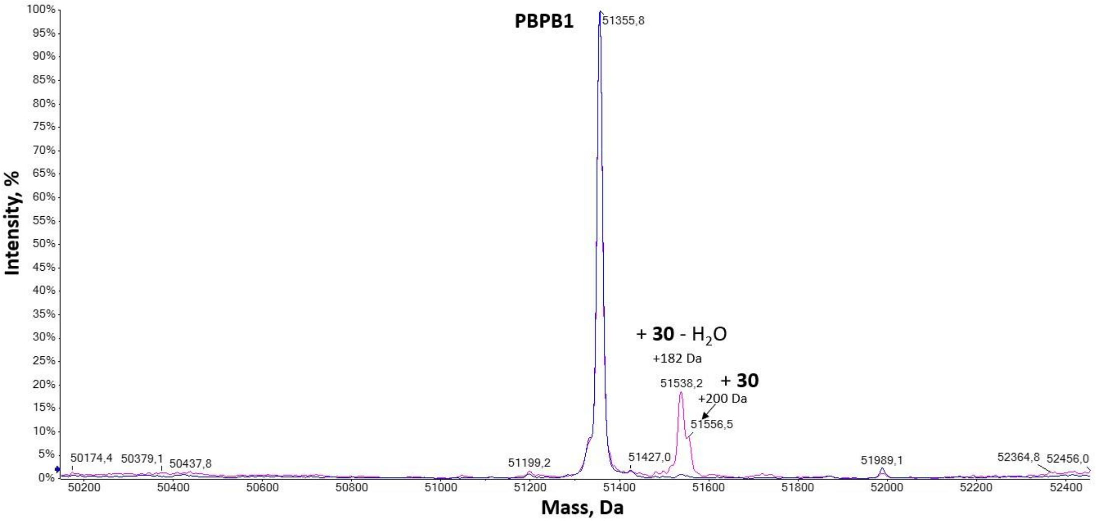

3. LC-MS chromatograms and spectra, ^{1}H and ^{13}C NMR spectra of compounds

[1-(1-Methyl-1*H*-1,2,3-triazole-4-carbonyl)pyrrolidin-3-yl]boronic acid (6)

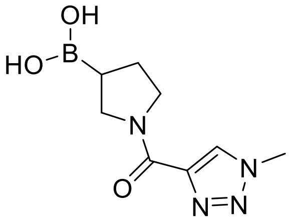

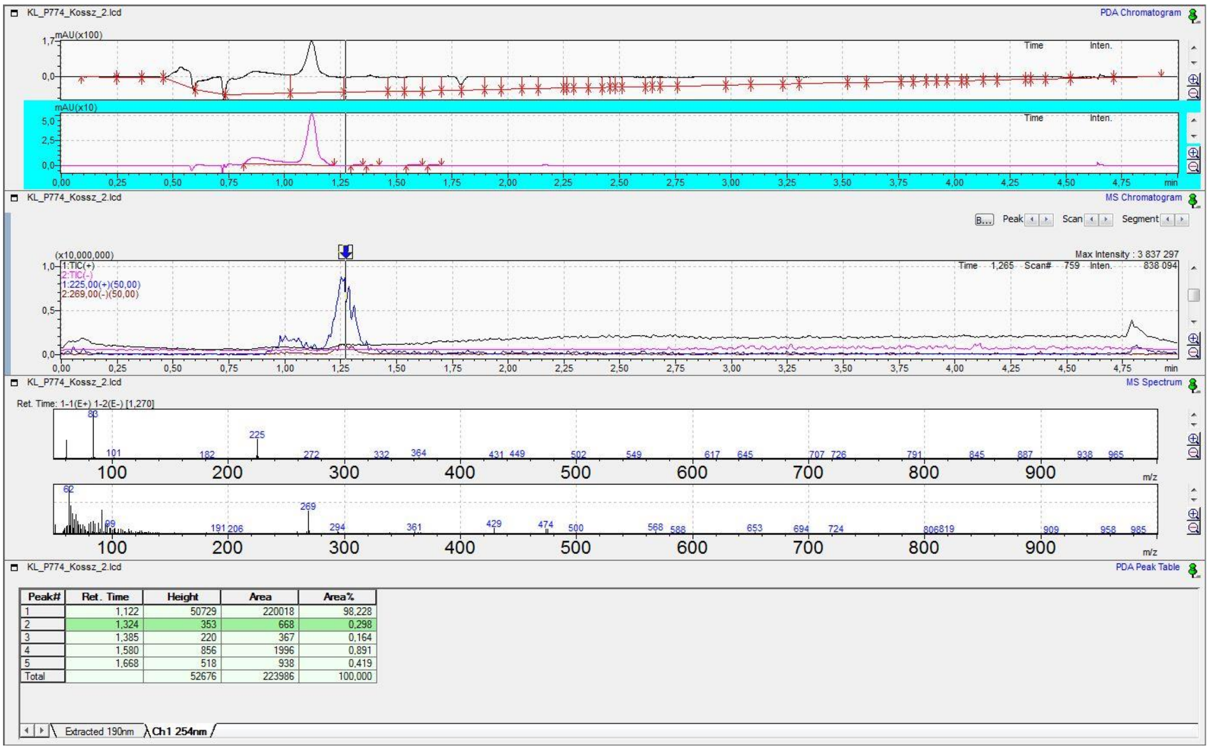

Peak#	Ret. Time	Height	Area	Area%
1	1,122	50729	220018	98,228
2	1,324	353	668	0,298
3	1,385	220	367	0,164
4	1,580	856	1996	0,891
5	1,668	518	938	0,419
Total		52676	223986	100,000

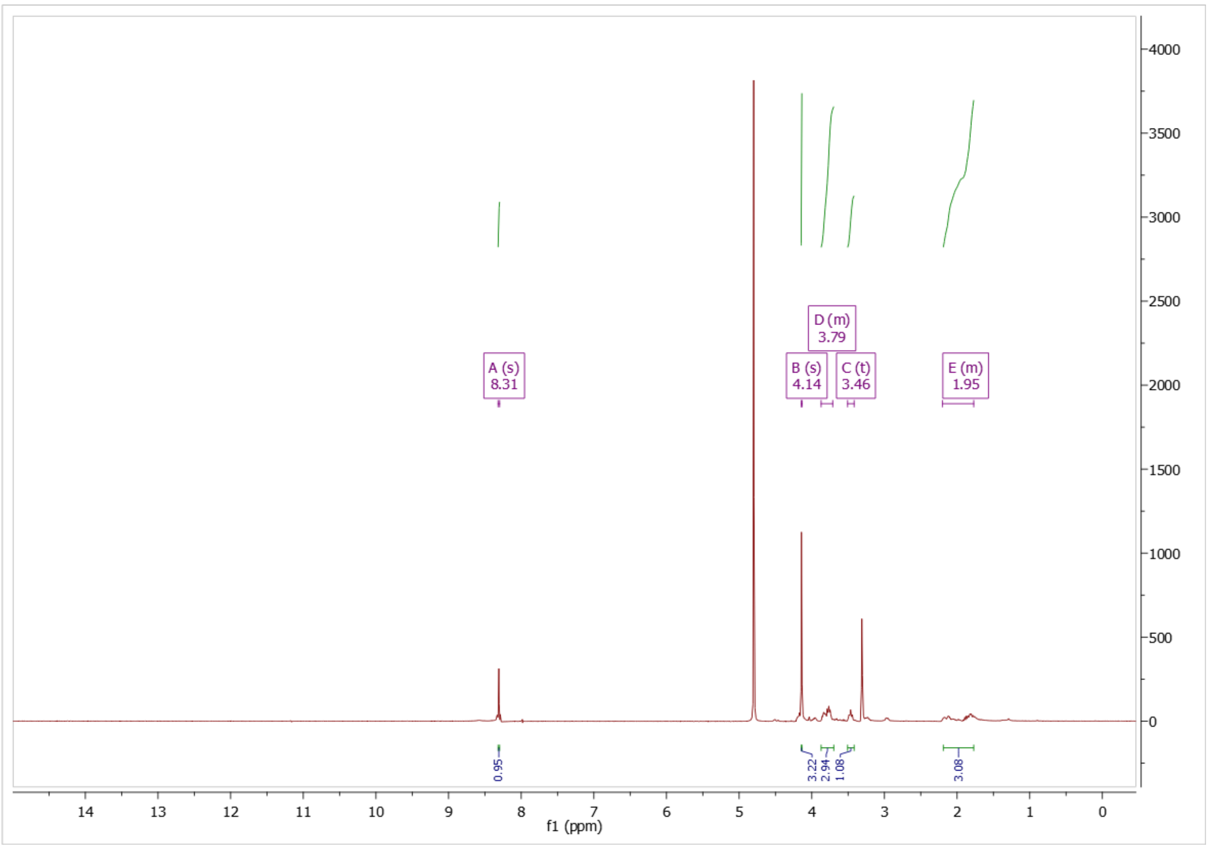

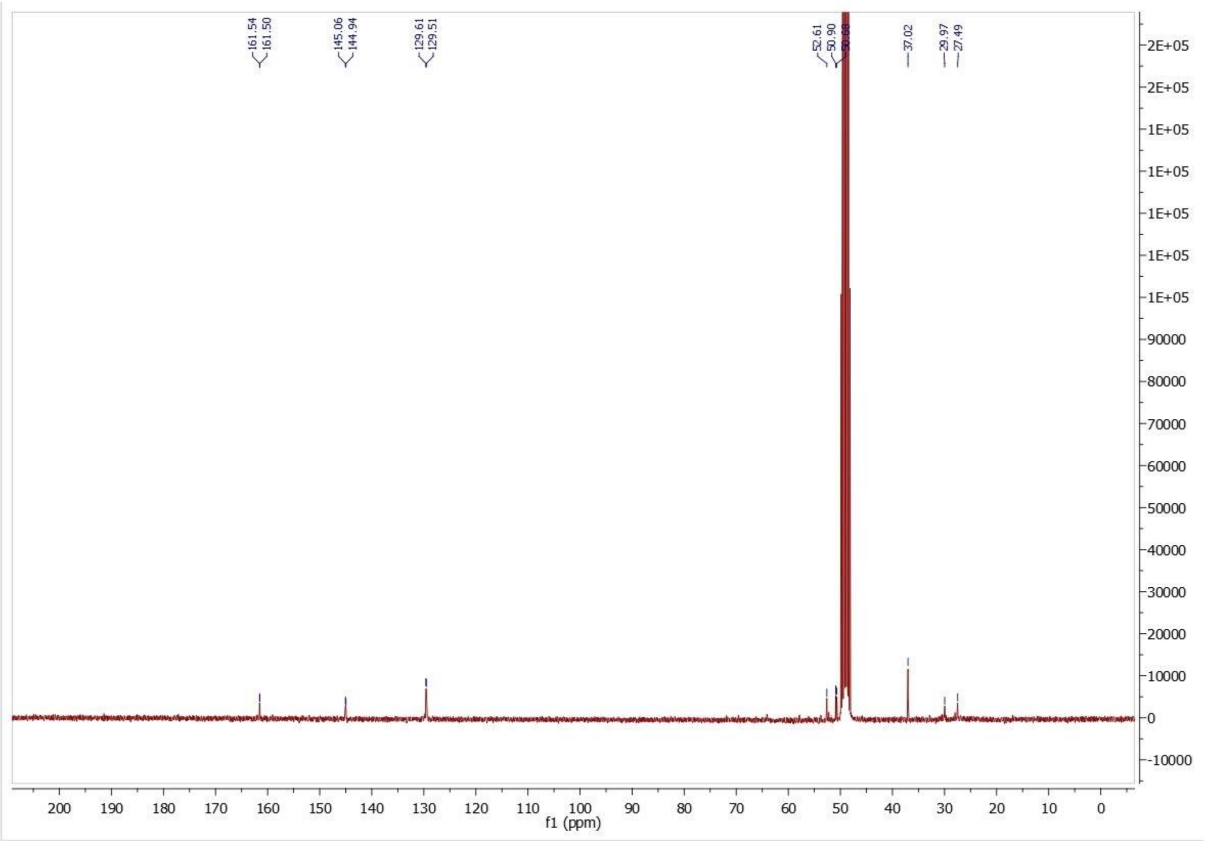

1-Methyl-4-[(2*R*)-2-[(1*S*,2*S*,6*R*,8*S*)-2,9,9-trimethyl-3,5-dioxa-4-boratricyclo[6.1.1.0^{2,6}]decan-4-yl]pyrrolidine-1-carbonyl]-1*H*-1,2,3-triazole (13)

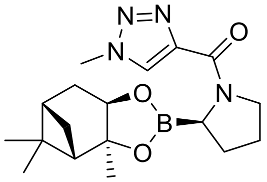

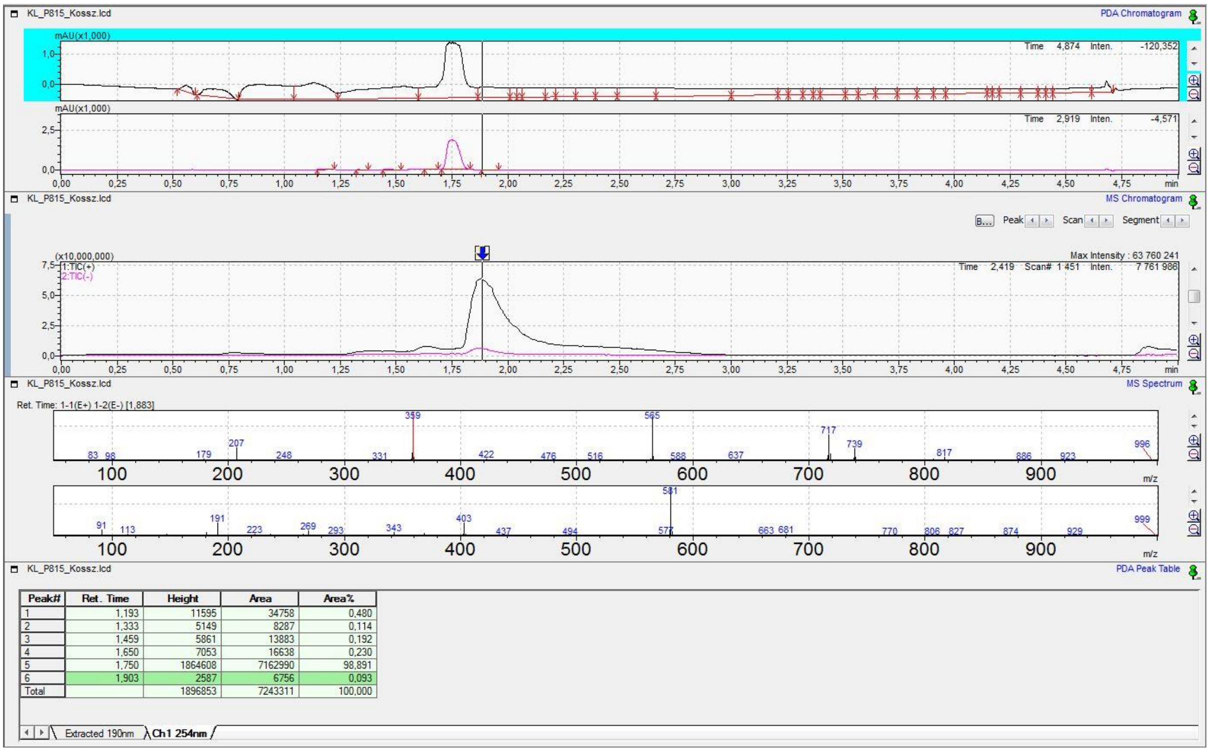

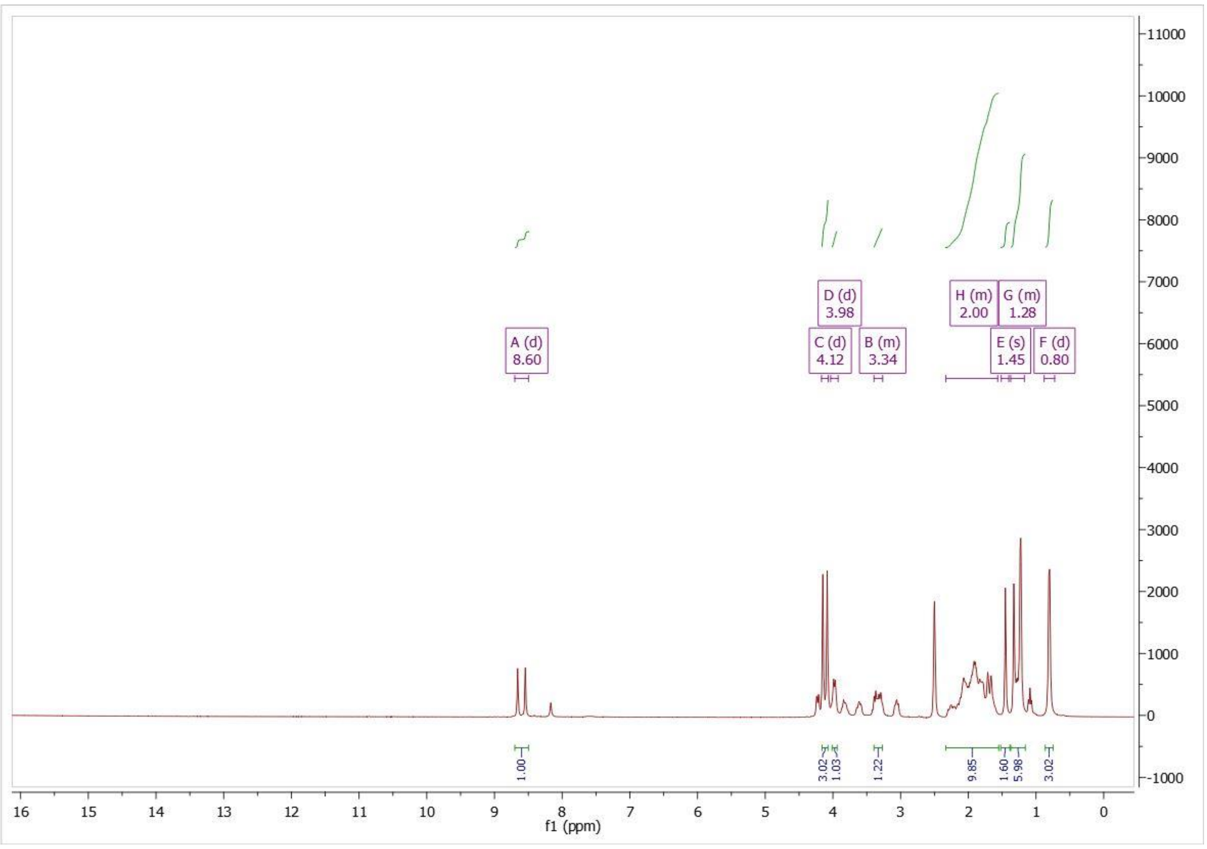

[(2*R*)-1-(1-Methyl-1*H*-1,2,3-triazole-4-carbonyl)pyrrolidin-2-yl]boronic acid (7)

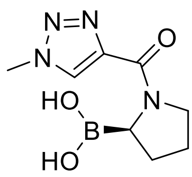

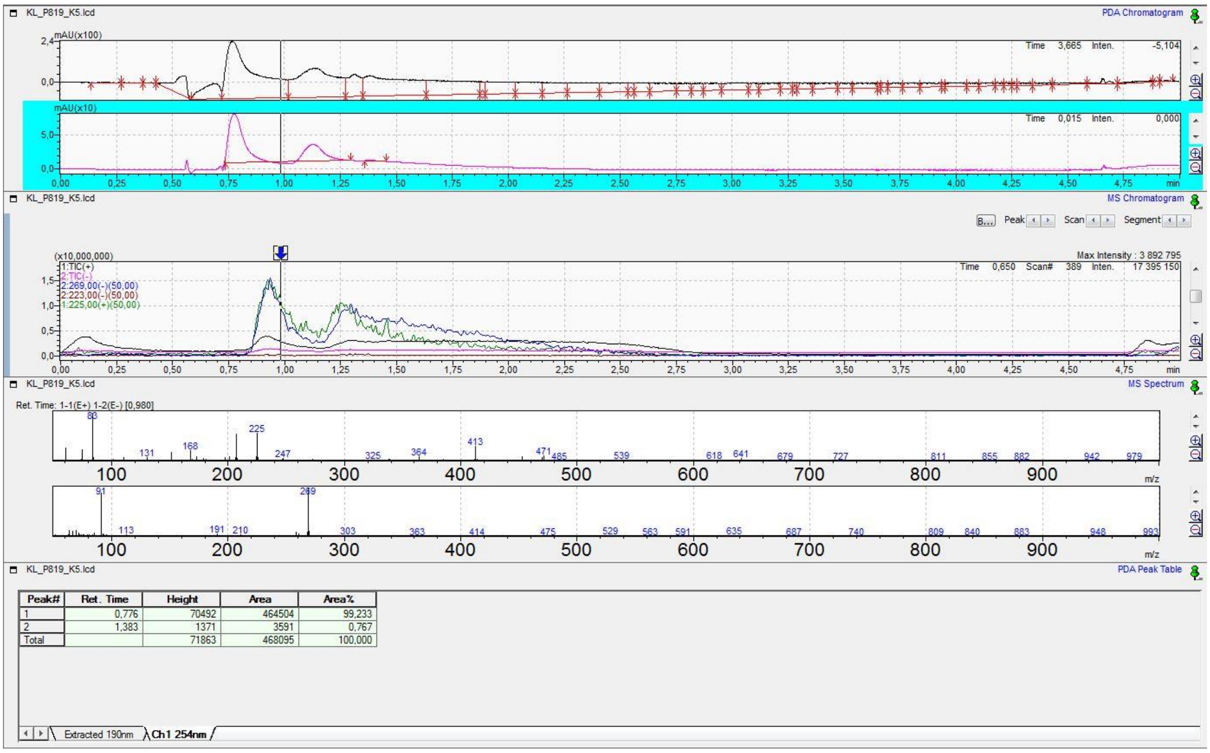

Peak#	Ret. Time	Height	Area	Area%
1	0,776	70492	464504	99,233
2	1,383	1371	3591	0,767
Total		71863	468095	100,000

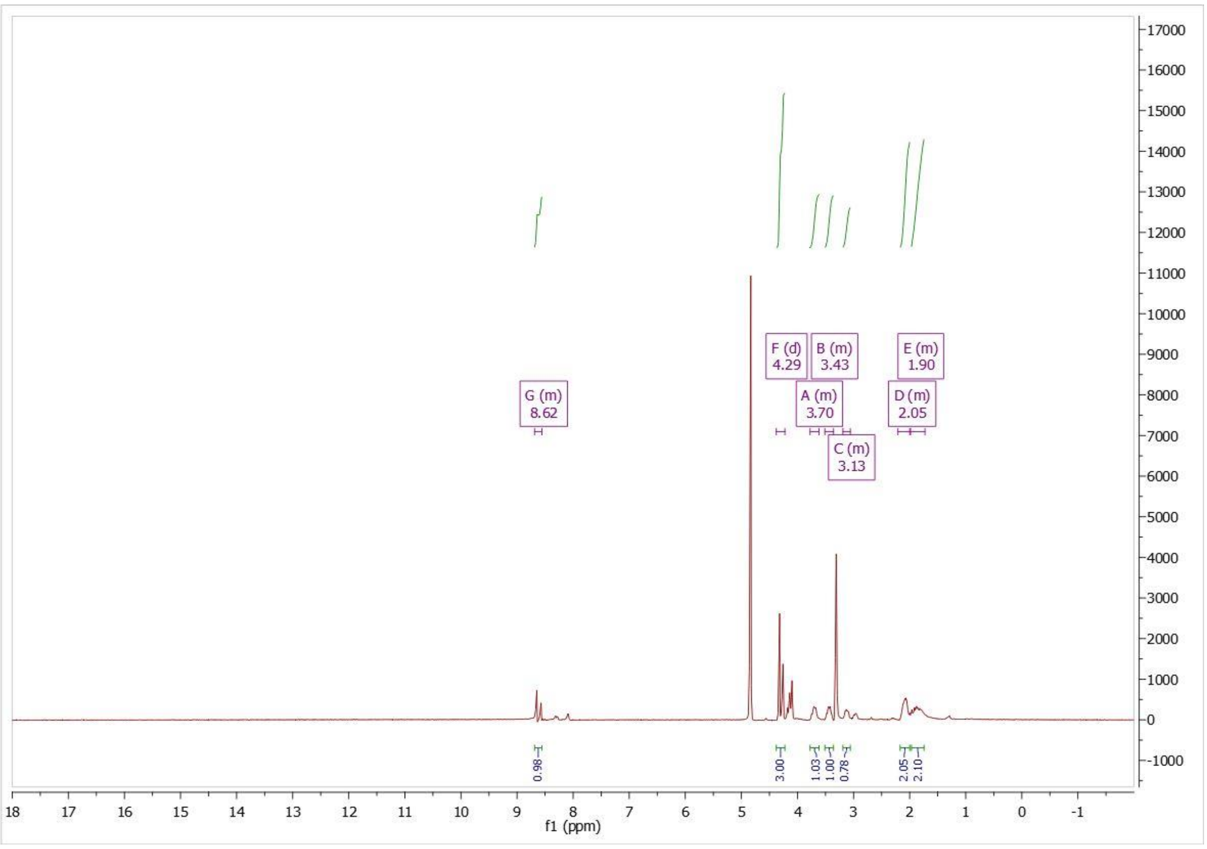

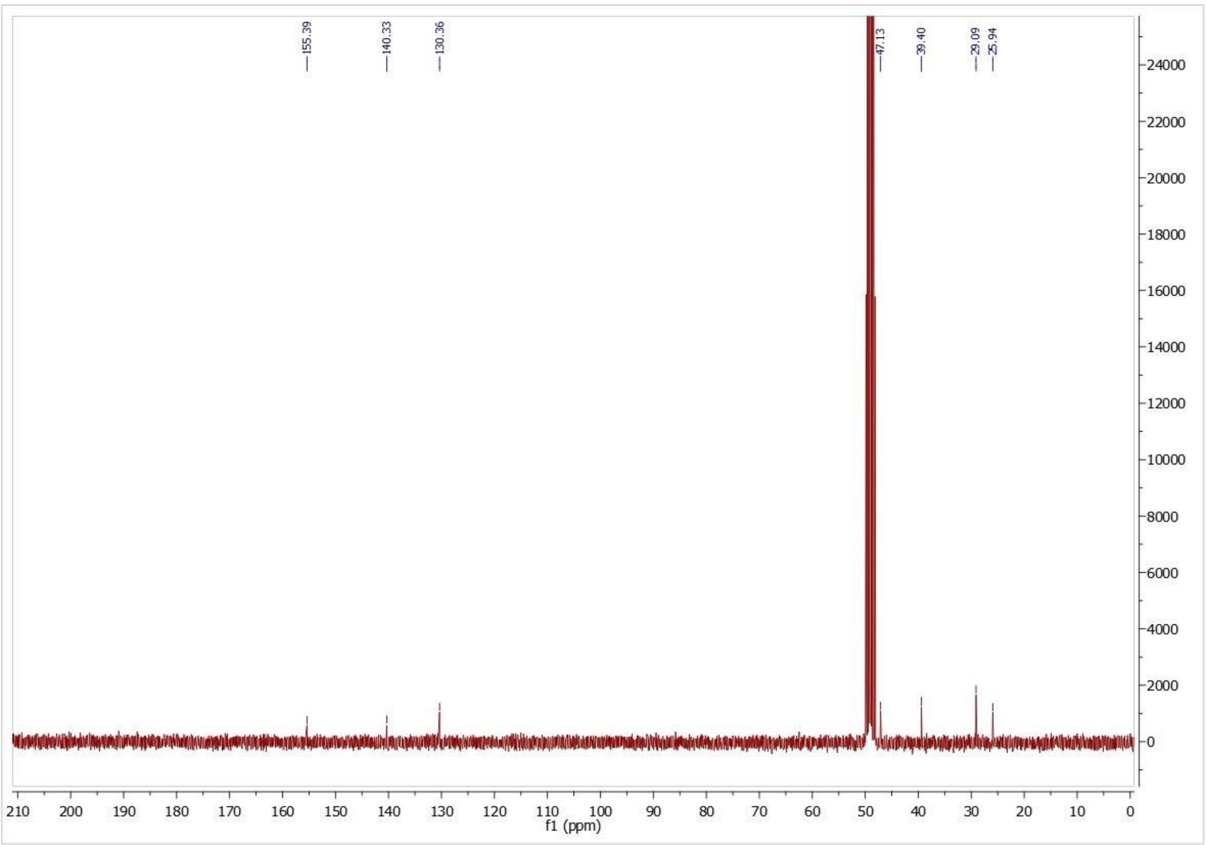

[[(1-Methyl-1*H*-1,2,3-triazol-4-yl)formamido]methyl]boronic acid (8)

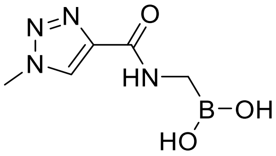

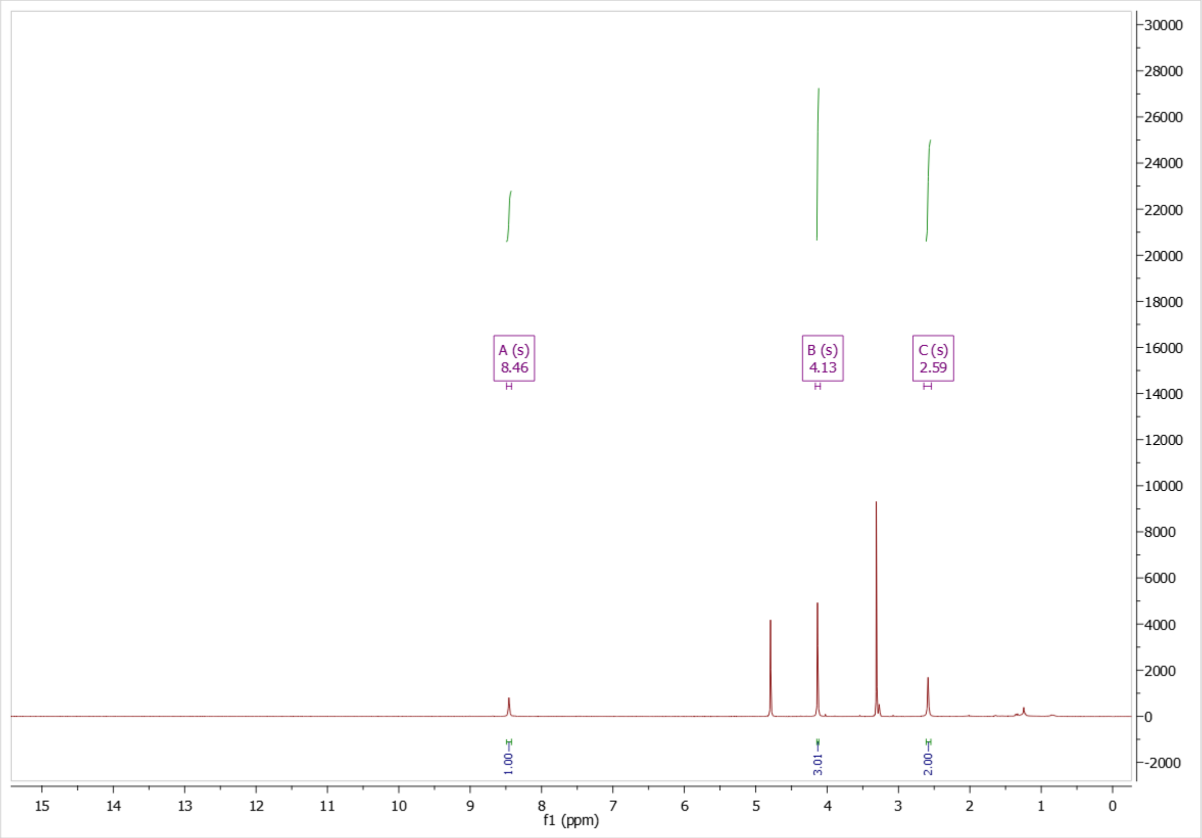
A (s)
8.46
B (s)
4.13
C (s)
2.59
1.00
3.01
2.00
f1 (ppm)

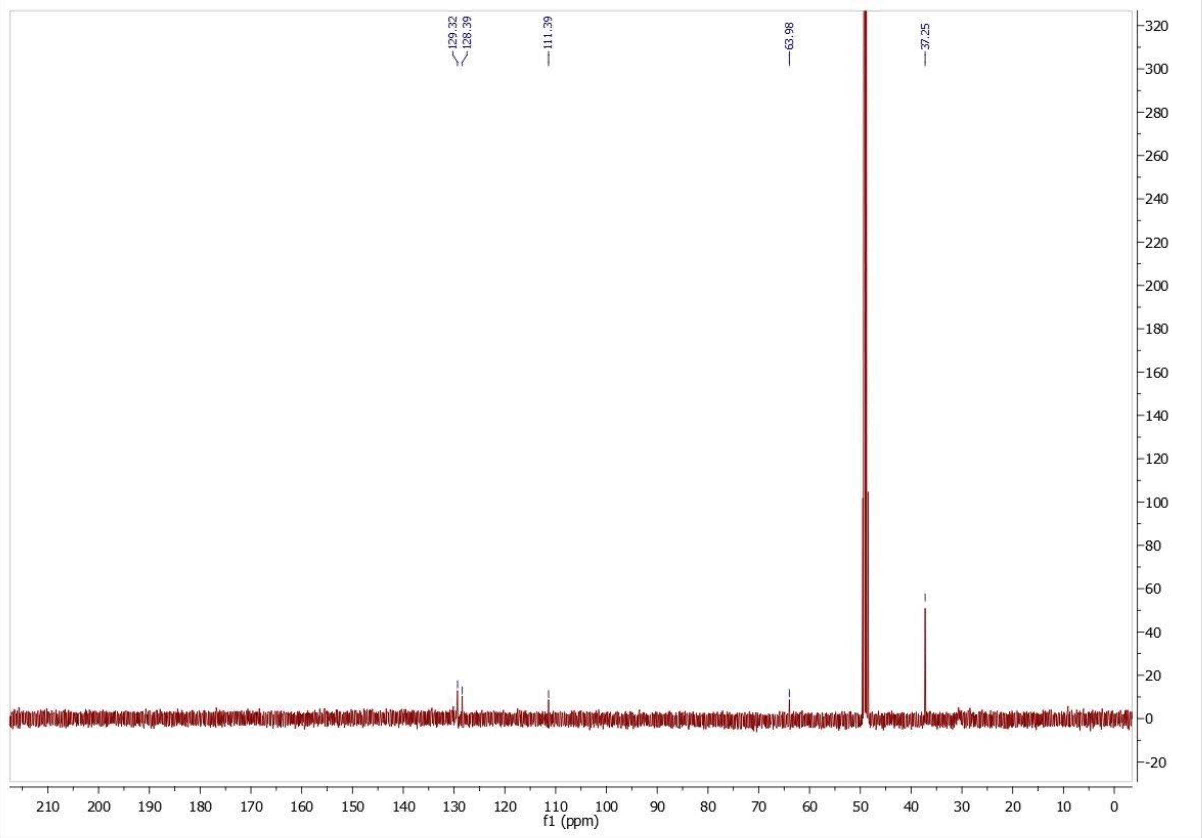
129.32
128.39
111.39
63.98
37.25
f1 (ppm)

2-Fluorobenzamide (21)

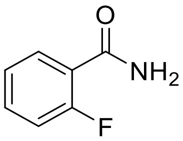

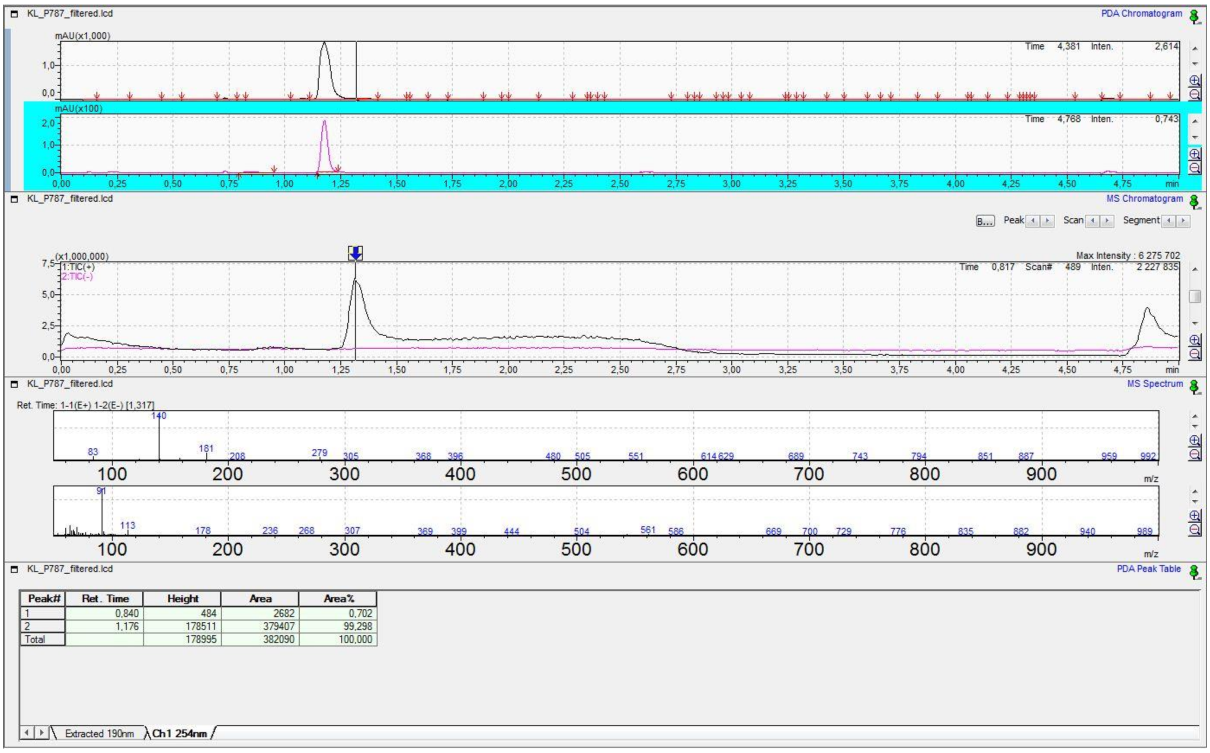

Peak#	Ret. Time	Height	Area	Area%
1	0,840	484	2682	0,702
2	1,176	178511	379407	99,298
Total		178995	382090	100,000

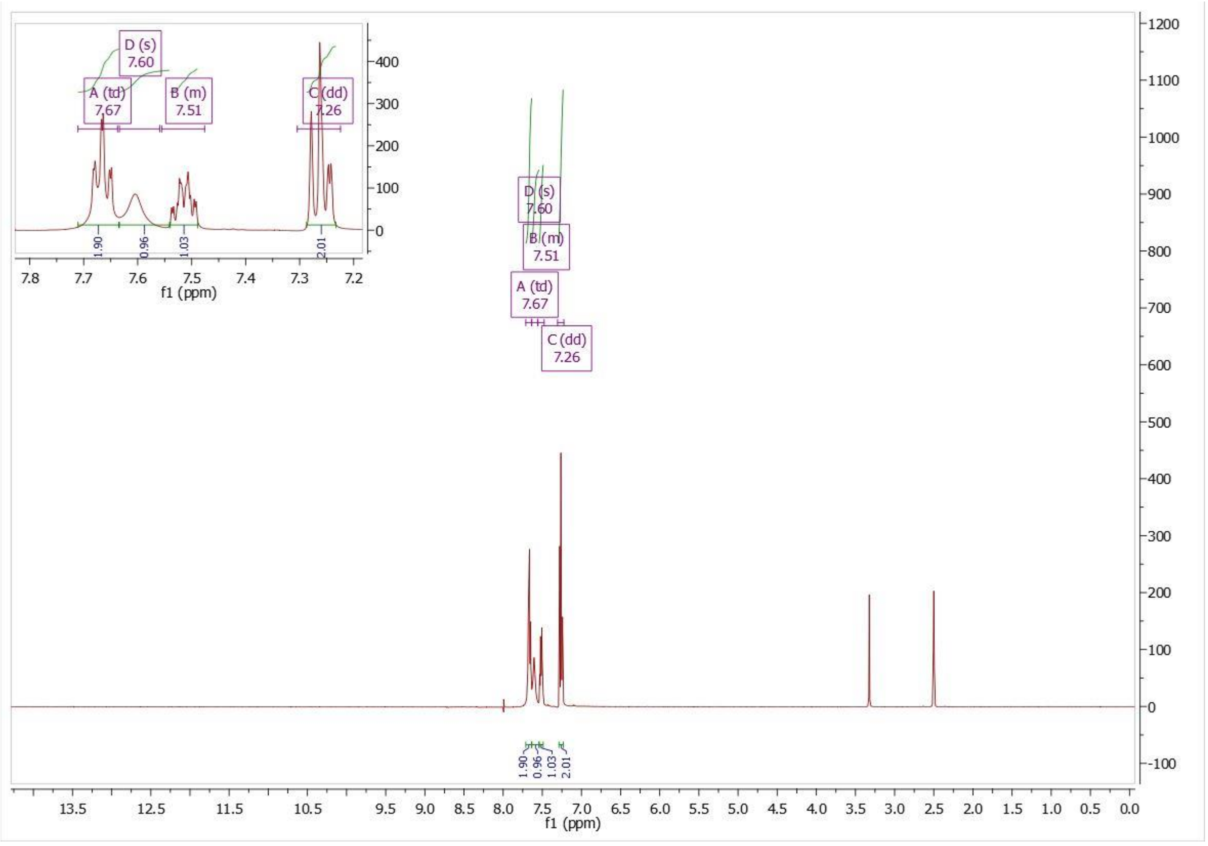
D (s) 7.60
A (td) 7.67
B (m) 7.51
C (dd) 7.26
1.90
0.96
1.03
2.01
f1 (ppm)

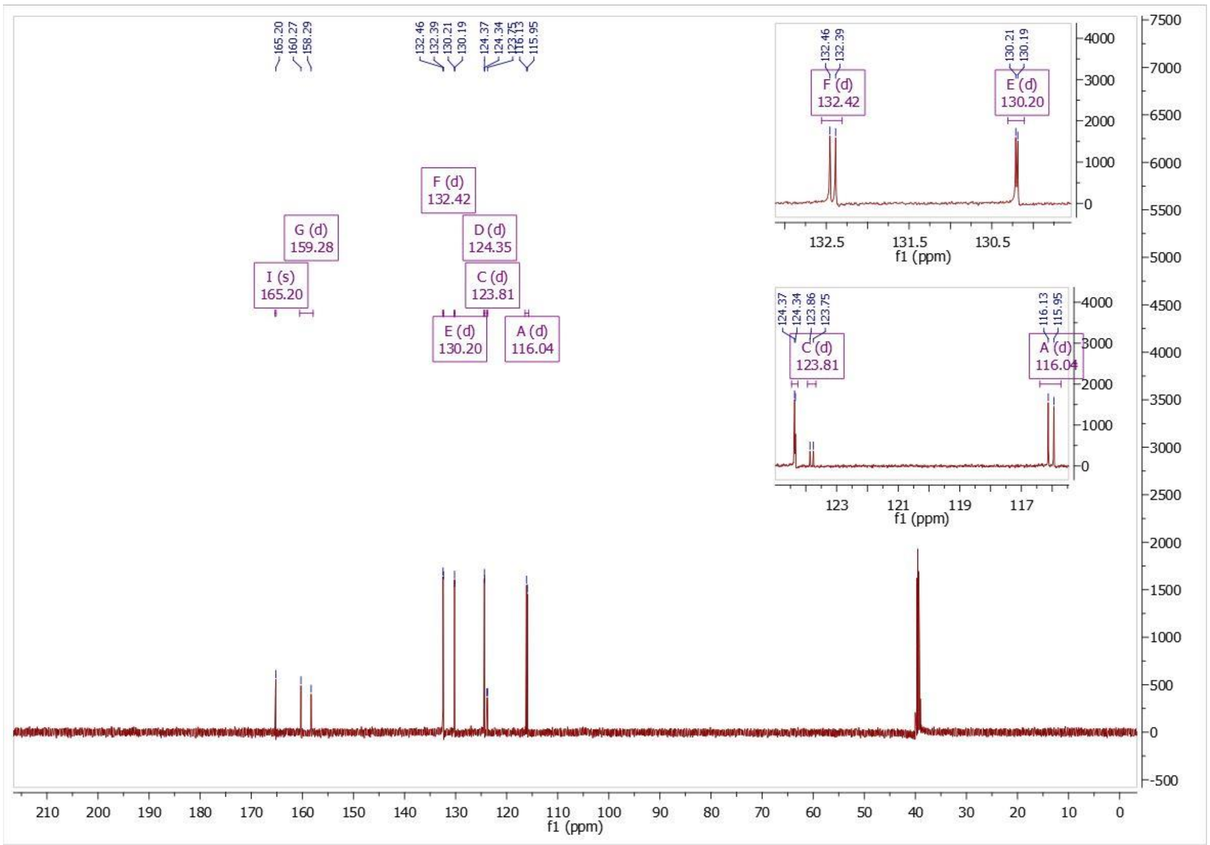
165.20
160.27
158.29
132.46
132.39
130.21
130.19
124.37
124.34
115.95
F (d) 132.42
E (d) 130.20
G (d) 159.28
D (d) 124.35
I (s) 165.20
C (d) 123.81
A (d) 116.04
f1 (ppm)

Ethyl (2*Z*)-3-[(2-fluorophenyl)formamido]prop-2-enoate (22)

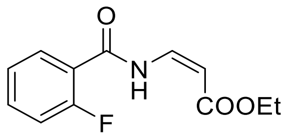

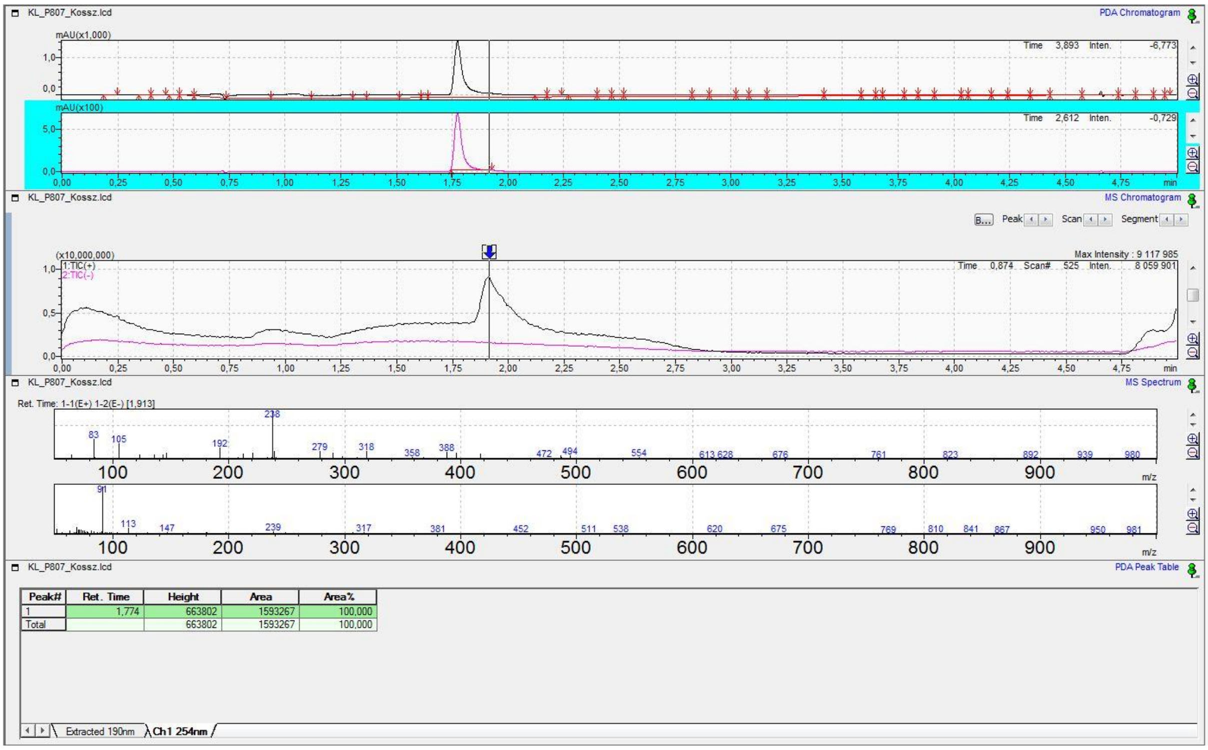

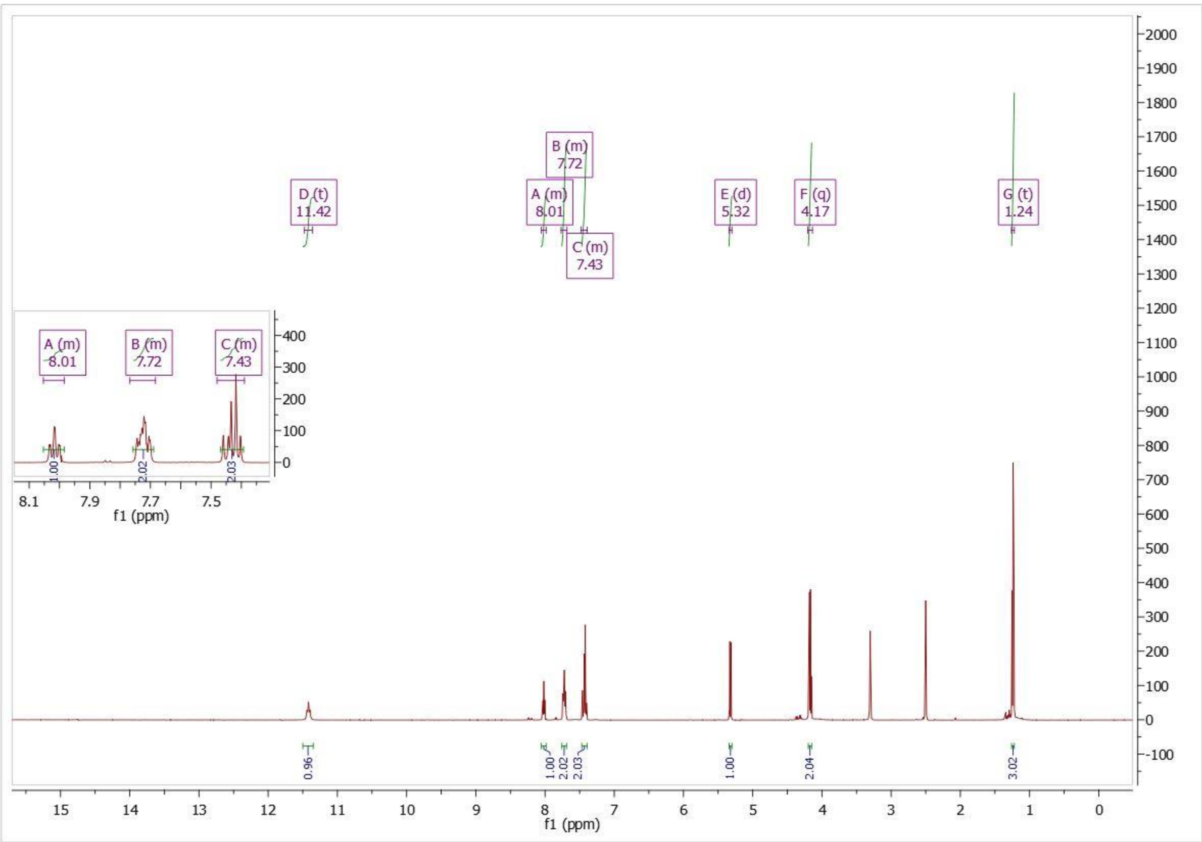

D (t)
11.42
A (m)
8.01
B (m)
7.72
C (m)
7.43
E (d)
5.32
F (q)
4.17
G (t)
1.24
f1 (ppm)

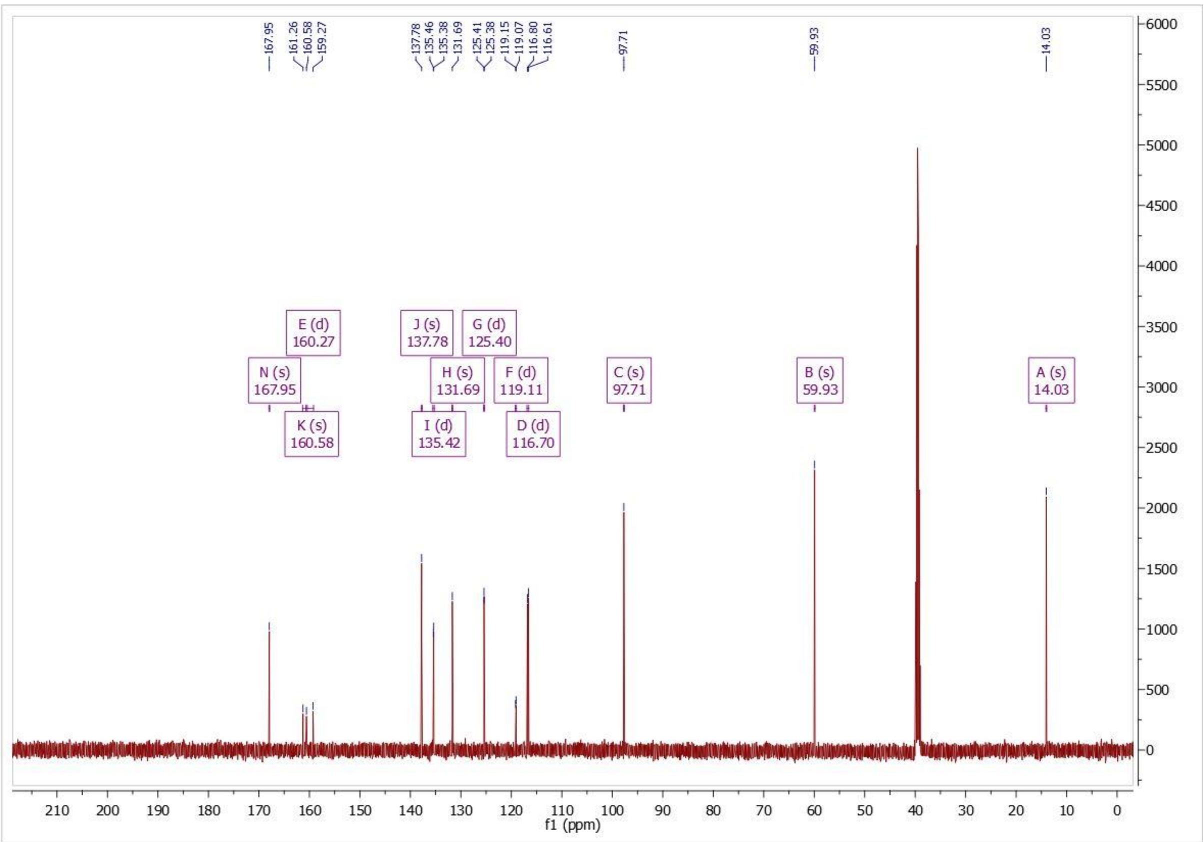

N (s)
167.95
E (d)
160.27
K (s)
160.58
J (s)
137.78
H (s)
131.69
I (d)
135.42
G (d)
125.40
F (d)
119.11
D (d)
116.70
C (s)
97.71
B (s)
59.93
A (s)
14.03
f1 (ppm)

Ethyl 3-[(2-fluorophenyl)formamido]-3-(4,4,5,5-tetramethyl-1,3,2-dioxaborolan-2-yl)propanoate (16)

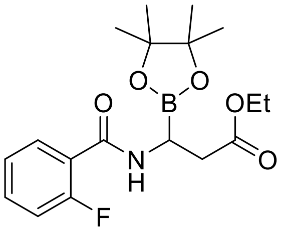

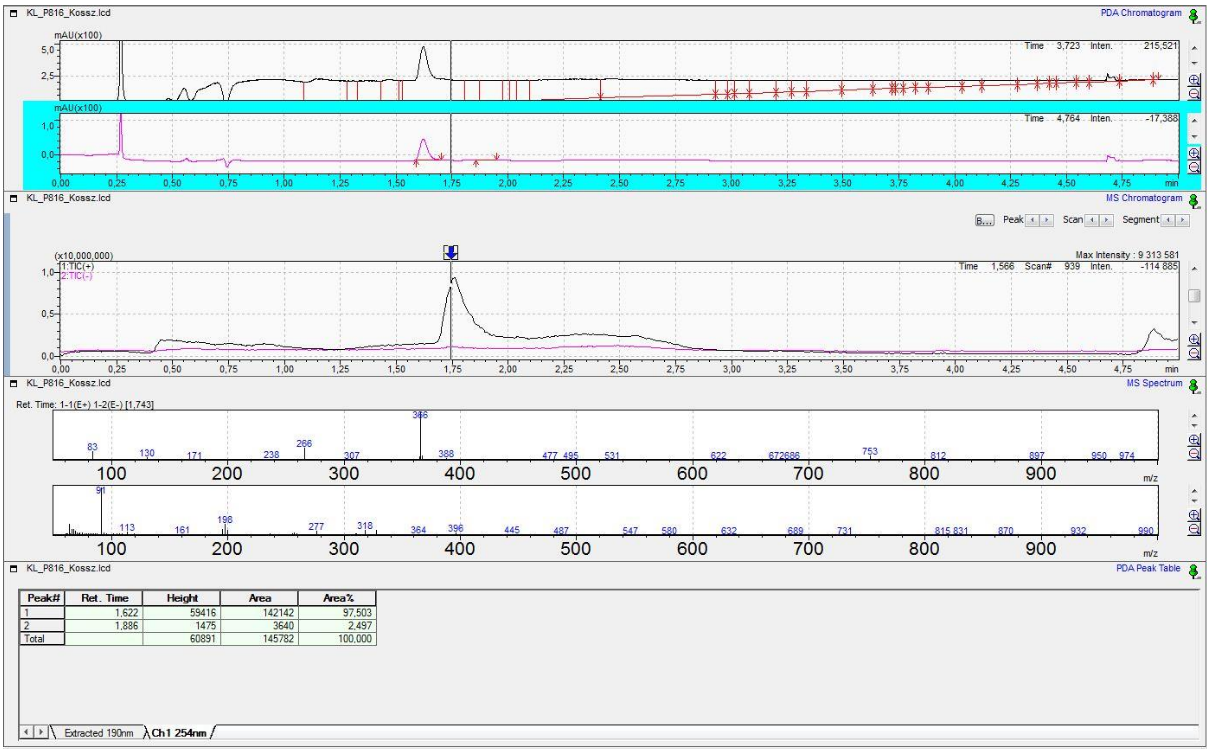

Peak#	Ret. Time	Height	Area	Area%
1	1,622	59416	142142	97,503
2	1,886	1475	3640	2,497
Total		60891	145782	100,000

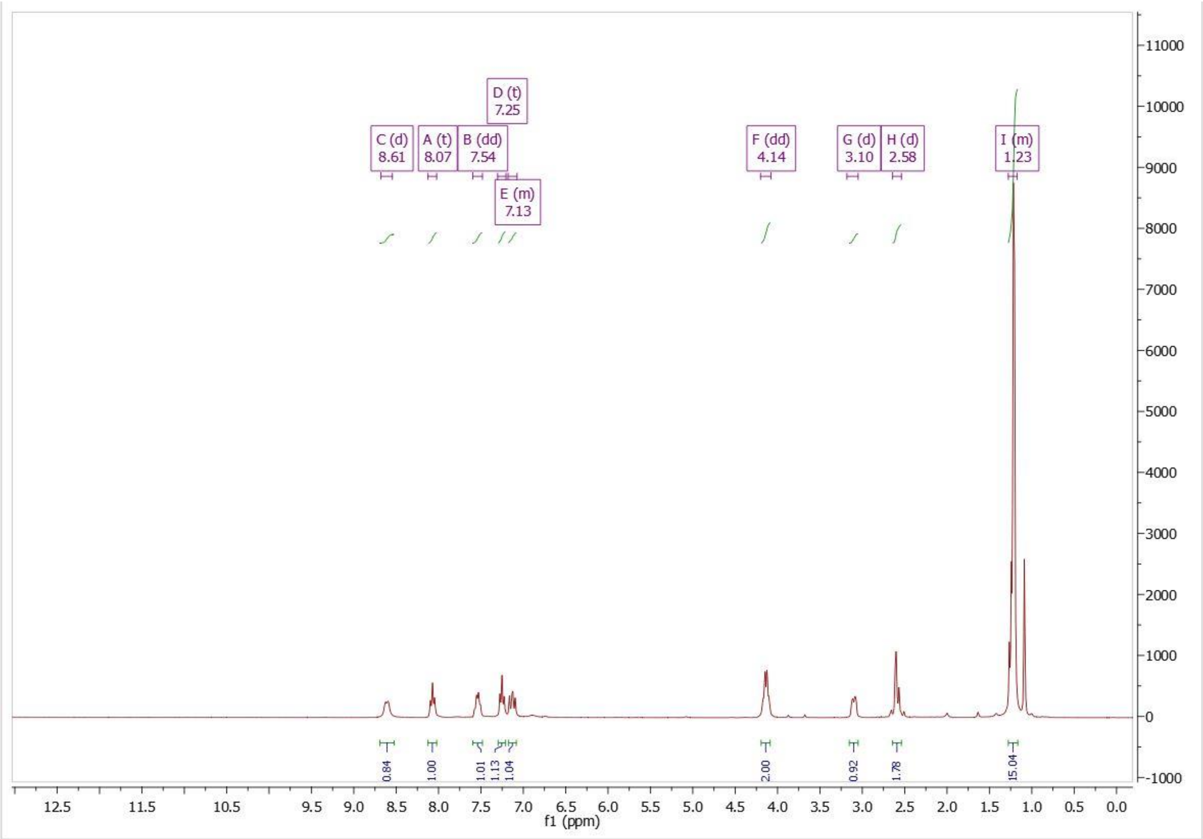
D (t) 7.25
C (d) 8.61
A (t) 8.07
B (dd) 7.54
E (m) 7.13
F (dd) 4.14
G (d) 3.10
H (d) 2.58
I (m) 1.23
0.84
1.00
1.01
1.13
1.04
2.00
0.92
1.78
15.04
f1 (ppm)

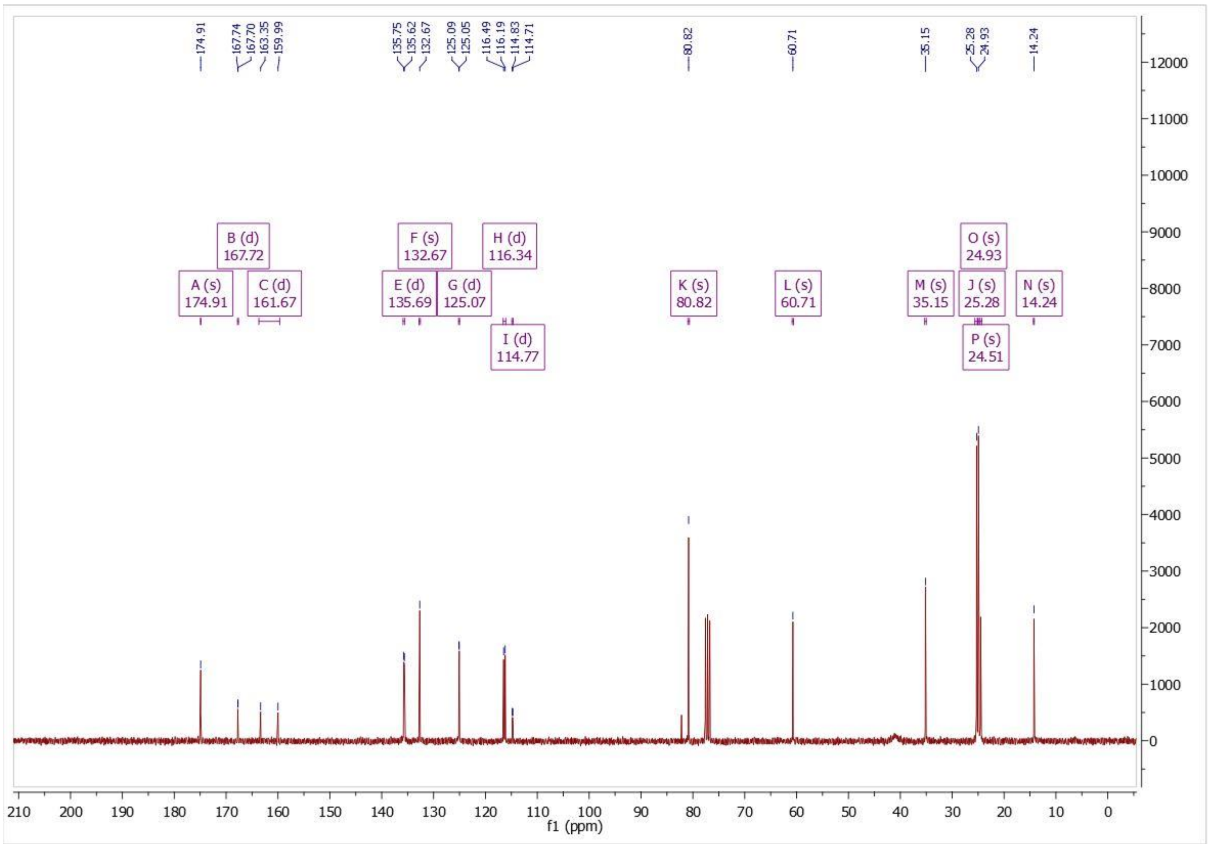
174.91
167.74
167.70
163.35
159.99
135.75
135.62
132.67
125.09
125.05
116.49
116.19
114.83
114.71
80.82
60.71
35.15
25.28
24.93
14.24
B (d) 167.72
A (s) 174.91
C (d) 161.67
F (s) 132.67
E (d) 135.69
G (d) 125.07
H (d) 116.34
I (d) 114.77
K (s) 80.82
L (s) 60.71
O (s) 24.93
M (s) 35.15
J (s) 25.28
N (s) 14.24
P (s) 24.51
f1 (ppm)

2-Fluoro-*N*-(2-hydroxy-5-oxo-1,2-oxaborolan-3-yl)benzamide (15)

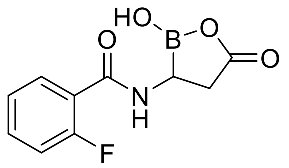

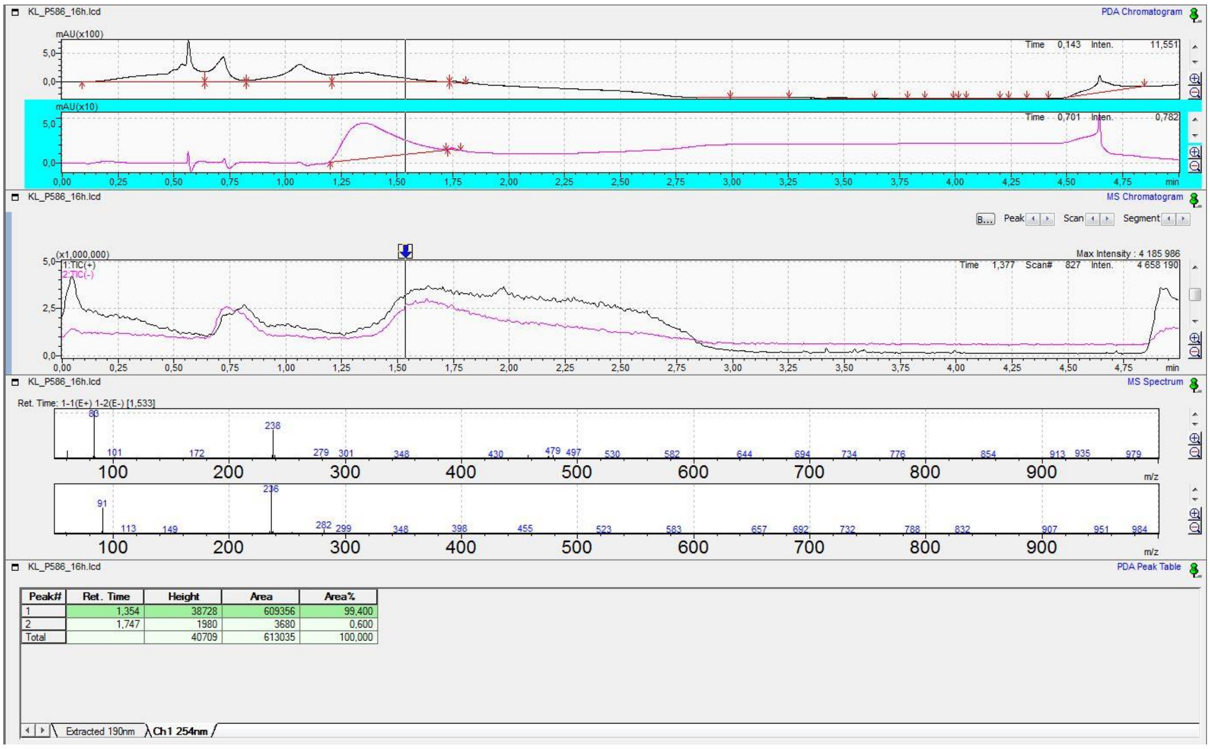

Peak#	Ret. Time	Height	Area	Area%
1	1,354	38728	609356	99,400
2	1,747	1980	3680	0,600
Total		40709	613035	100,000

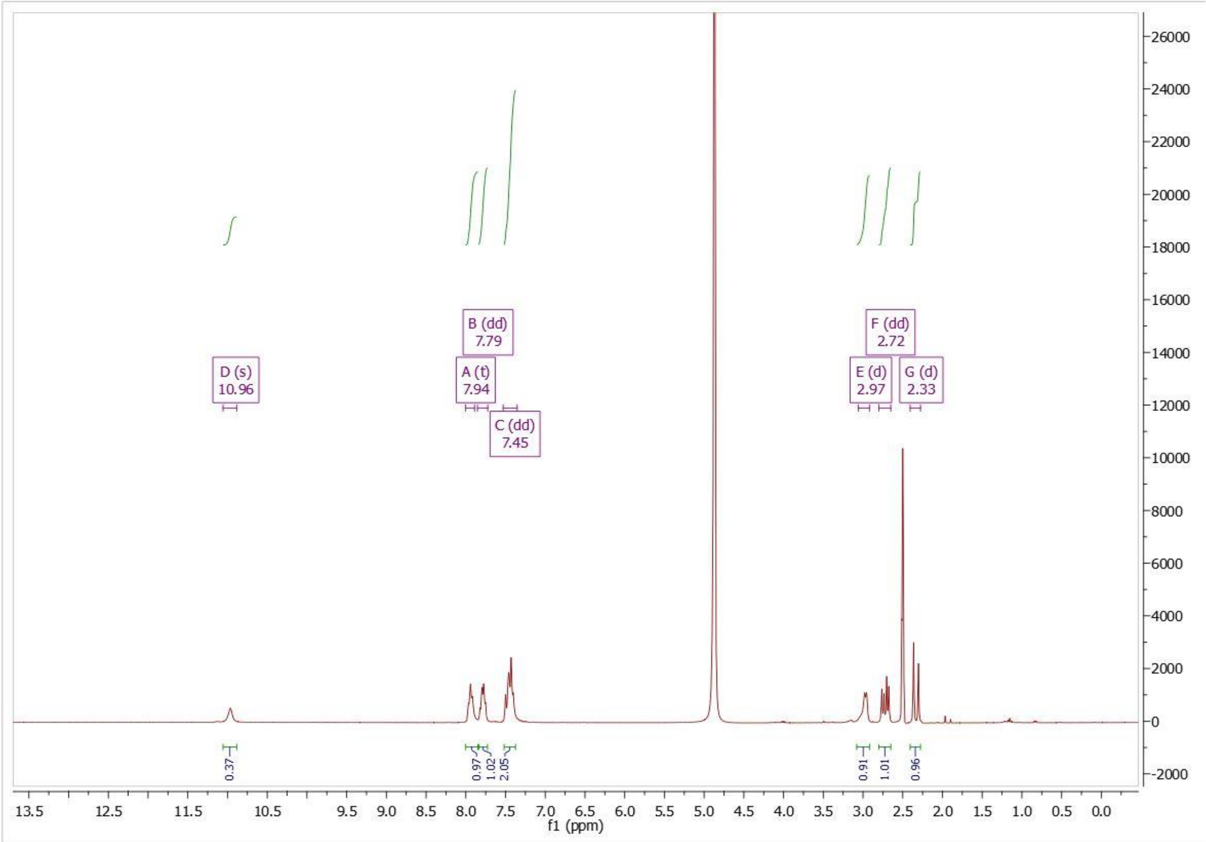

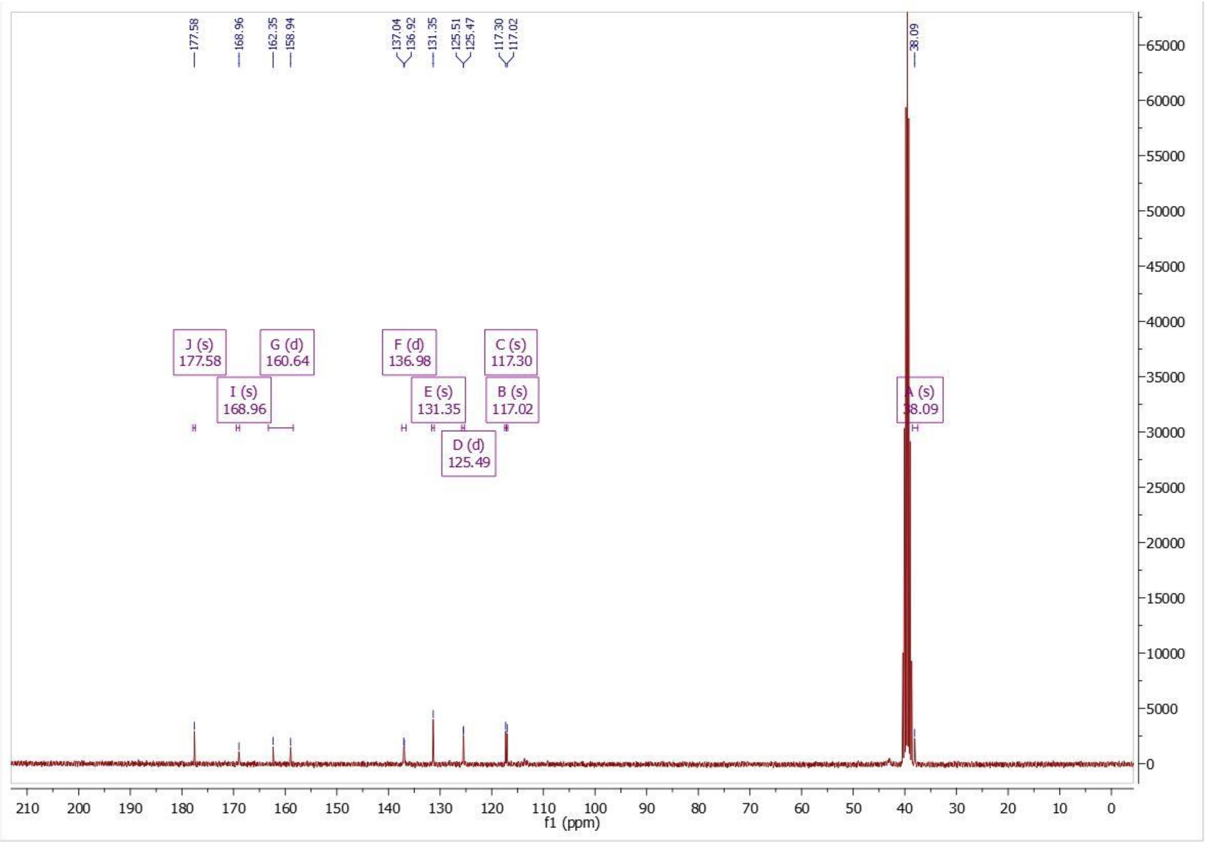

(2*Z*)-3-[(2-fluorophenyl)formamido]prop-2-enoic acid (23)

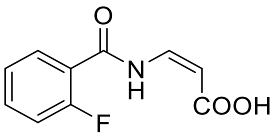

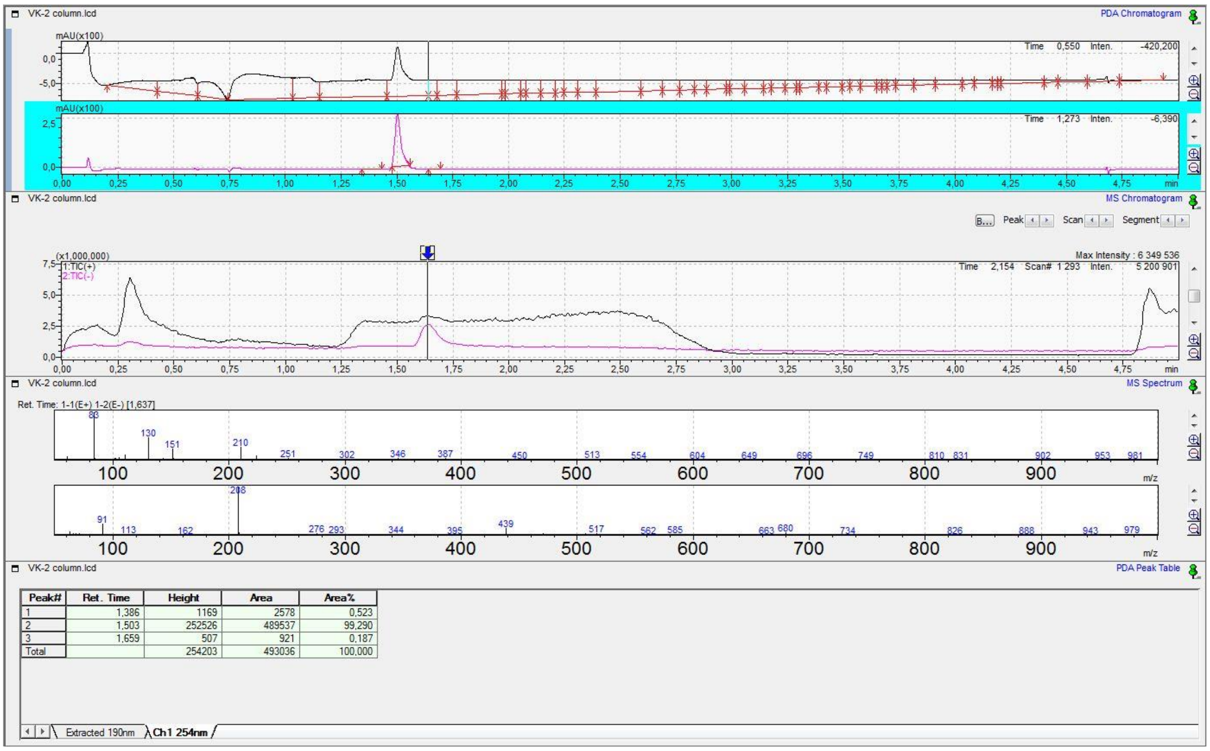
VK-2 column.lcd
PDA Chromatogram
MS Chromatogram
Max Intensity : 6 349 536
1:TIC(+)
2:TIC(-)
MS Spectrum
Ret. Time: 1-1(E+) 1-2(E-) [1,637]
PDA Peak Table
Peak# Ret. Time Height Area Area%
1 1,386 1169 2578 0,523
2 1,503 252526 489537 99,290
3 1,659 507 921 0,187
Total 254203 493036 100,000
Extracted 190nm
Ch1 254nm

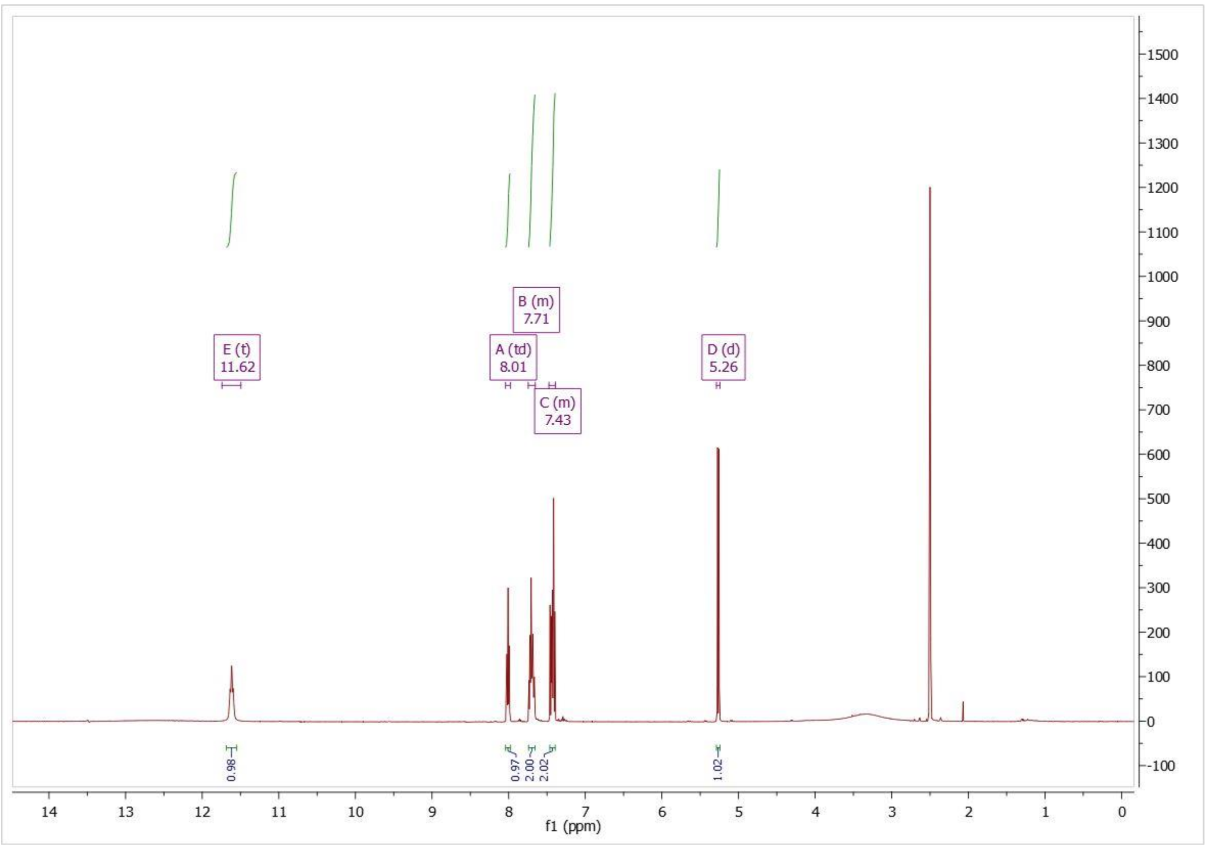
E (t)
11.62
A (td)
8.01
B (m)
7.71
C (m)
7.43
D (d)
5.26
0.98
0.97
2.00
2.02
1.02
f1 (ppm)

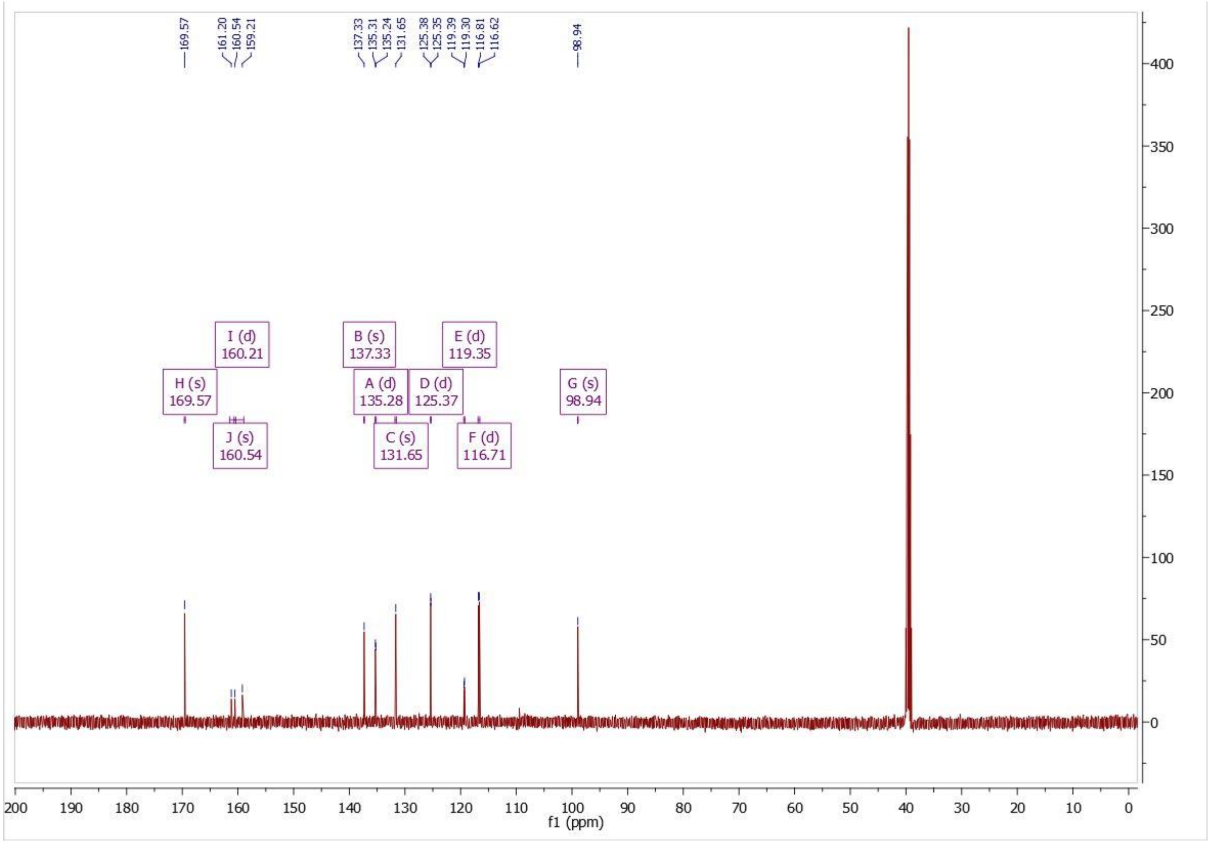

(2*Z*)-3-[(2-fluorophenyl)formamido]-*N*-phenylprop-2-enamide (24)

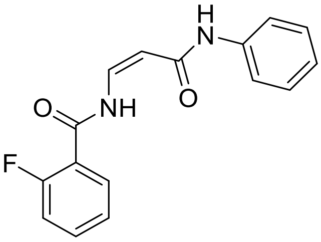

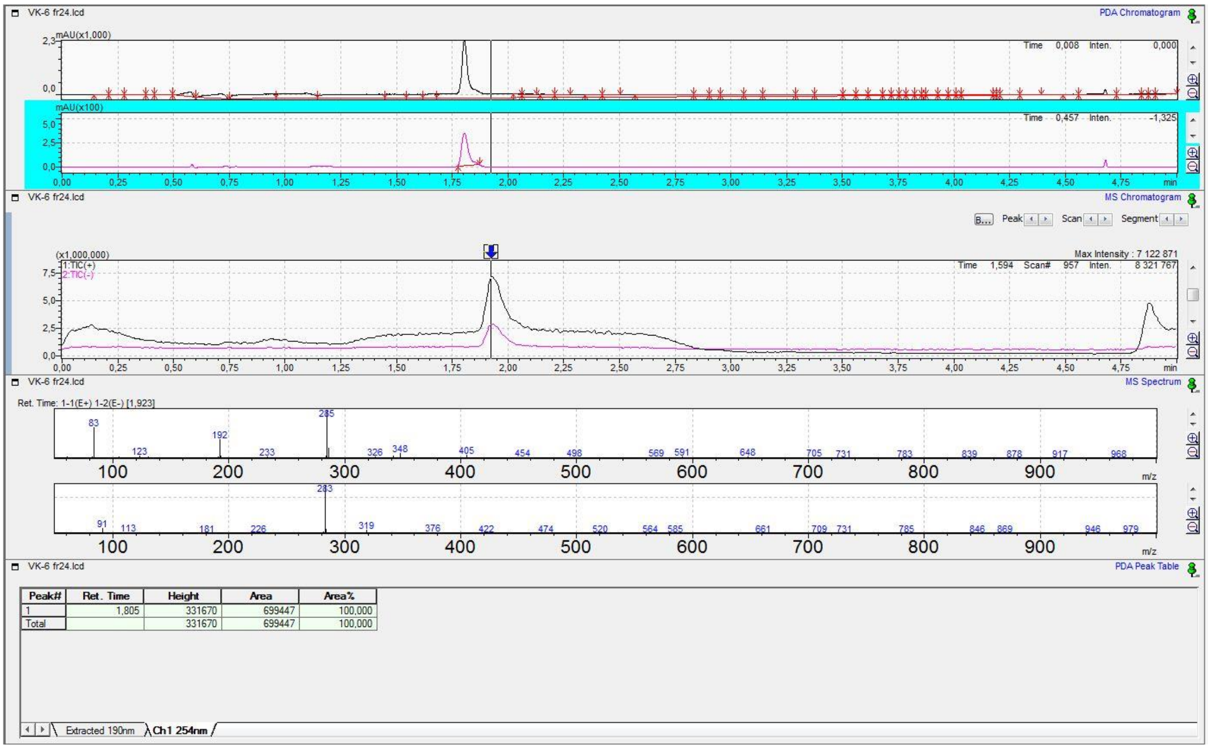
VK-6 fr24.lcd
PDA Chromatogram
mAU(x1,000)
mAU(x100)
MS Chromatogram
(x1,000,000)
1:TIC(+)
2:TIC(-)
Max Intensity : 7 122 871
MS Spectrum
Ret. Time: 1-1(E+) 1-2(E-) [1,923]
285
283
m/z
PDA Peak Table
Peak# Ret. Time Height Area Area%
1 1,805 331670 699447 100,000
Total 331670 699447 100,000
Extracted 190nm
Ch1 254nm

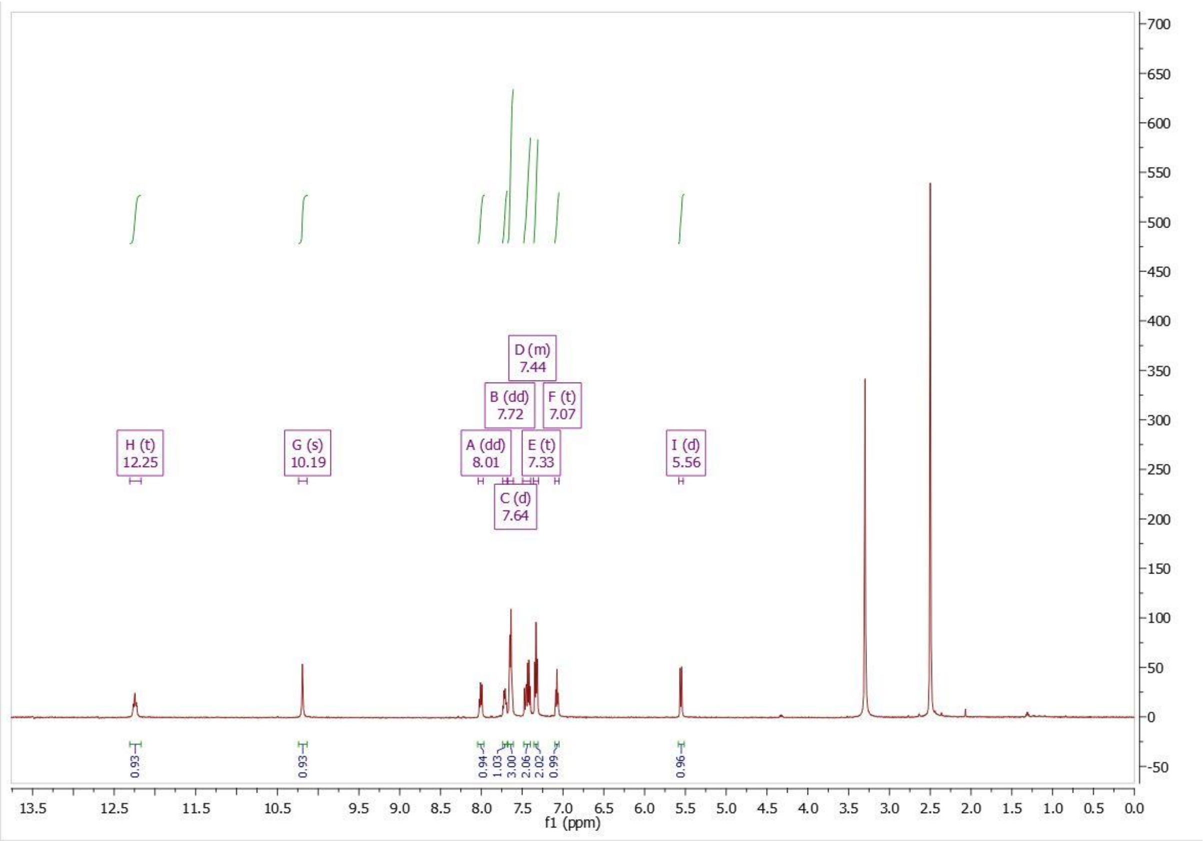
H (t) 12.25
G (s) 10.19
A (dd) 8.01
B (dd) 7.72
C (d) 7.64
D (m) 7.44
E (t) 7.33
F (t) 7.07
I (d) 5.56
0.93
0.93
0.94
1.03
3.00
2.06
2.02
0.99
0.96
f1 (ppm)

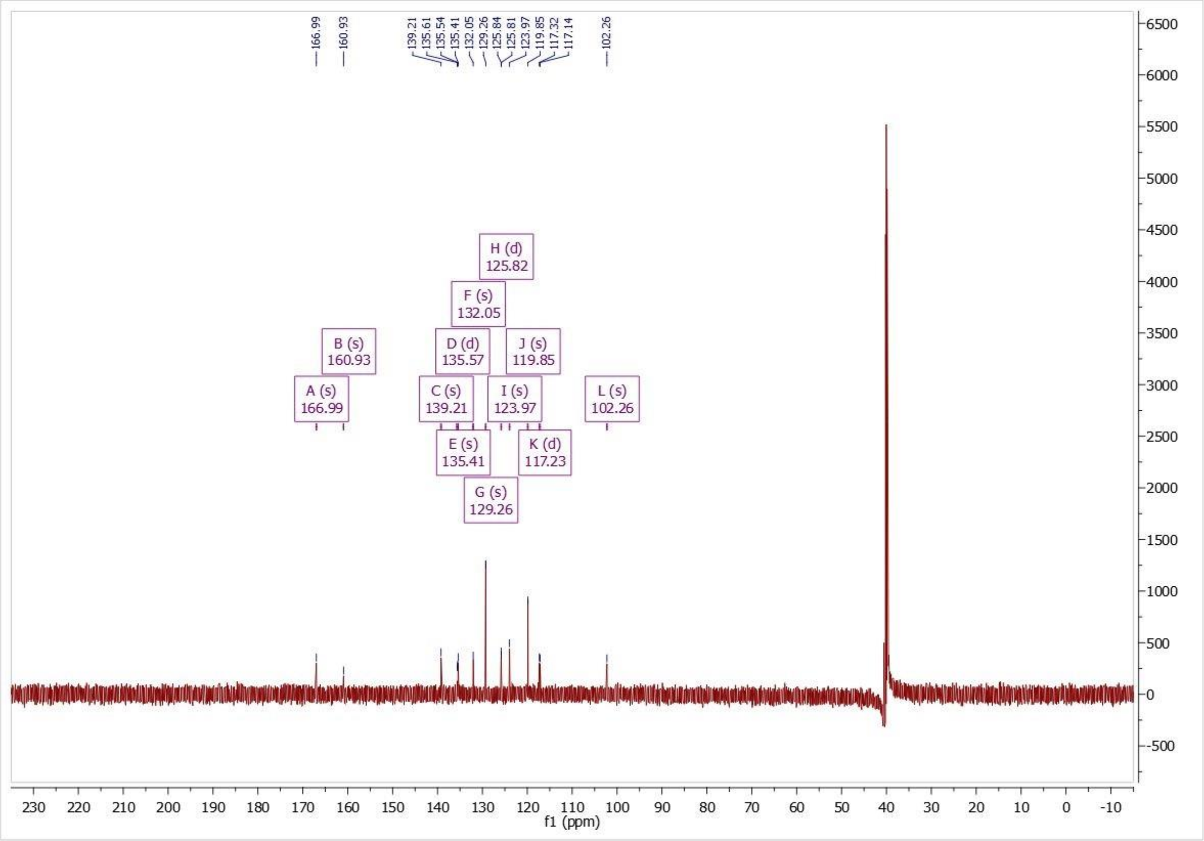

3-[(2-Fluorophenyl)formamido]-*N*-phenyl-3-(4,4,5,5-tetramethyl-1,3,2-dioxaborolan-2-yl)propanamide (17)

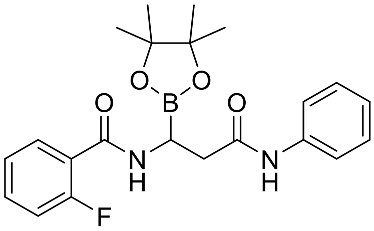

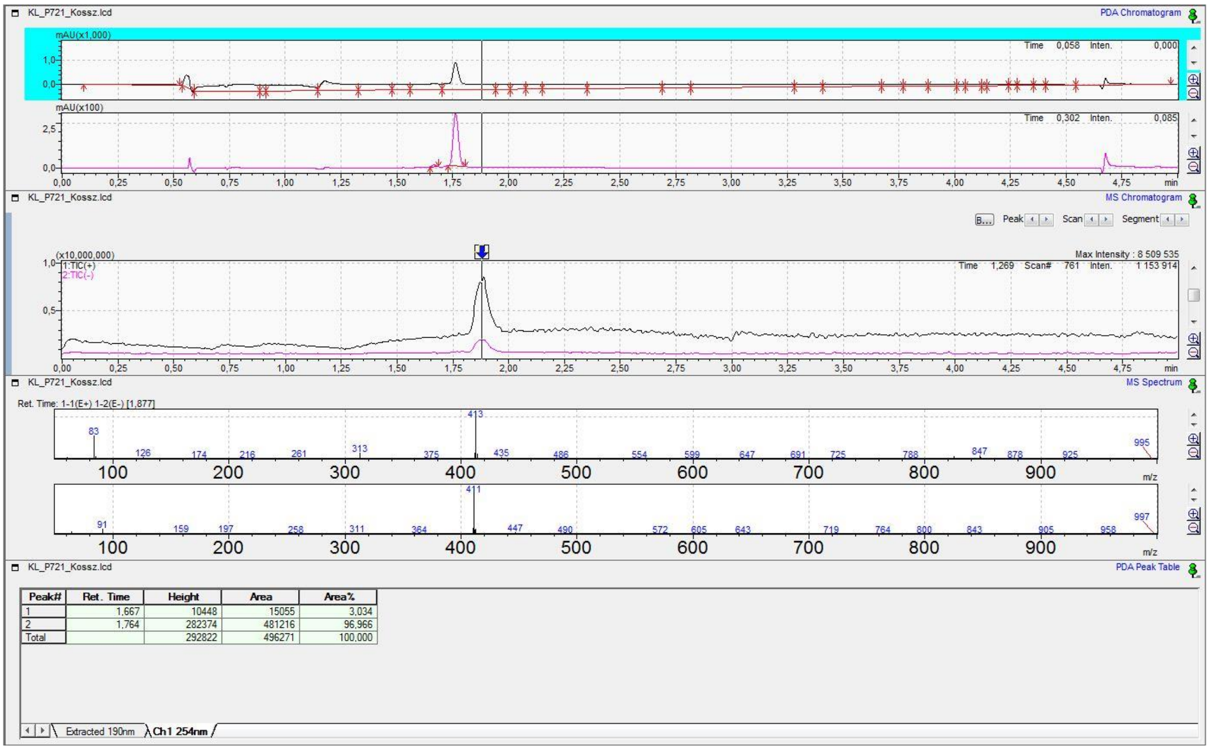
KL_P721_Kossz.lcd
PDA Chromatogram
MS Chromatogram
MS Spectrum
Ret. Time: 1-1(E+) 1-2(E-) [1,877]
1:TIC(+)
2:TIC(-)
Max Intensity : 8 509 535
PDA Peak Table
Peak# Ret. Time Height Area Area%
1 1,667 10448 15055 3,034
2 1,764 282374 481216 96,966
Total 292822 496271 100,000
Extracted 190nm
Ch1 254nm

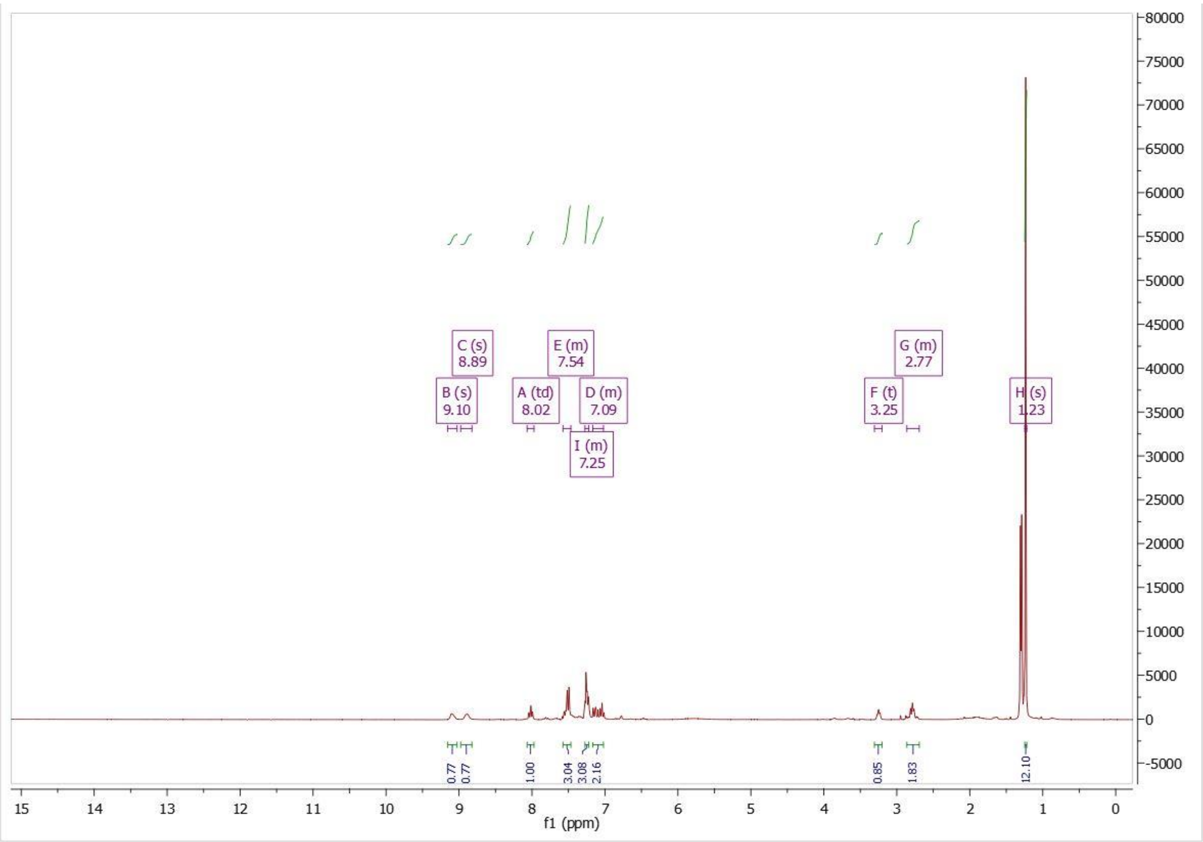
C (s) 8.89
E (m) 7.54
G (m) 2.77
B (s) 9.10
A (td) 8.02
D (m) 7.09
F (t) 3.25
H (s) 1.23
I (m) 7.25
0.77
0.77
1.00
3.04
3.08
2.16
0.85
1.83
12.10
f1 (ppm)

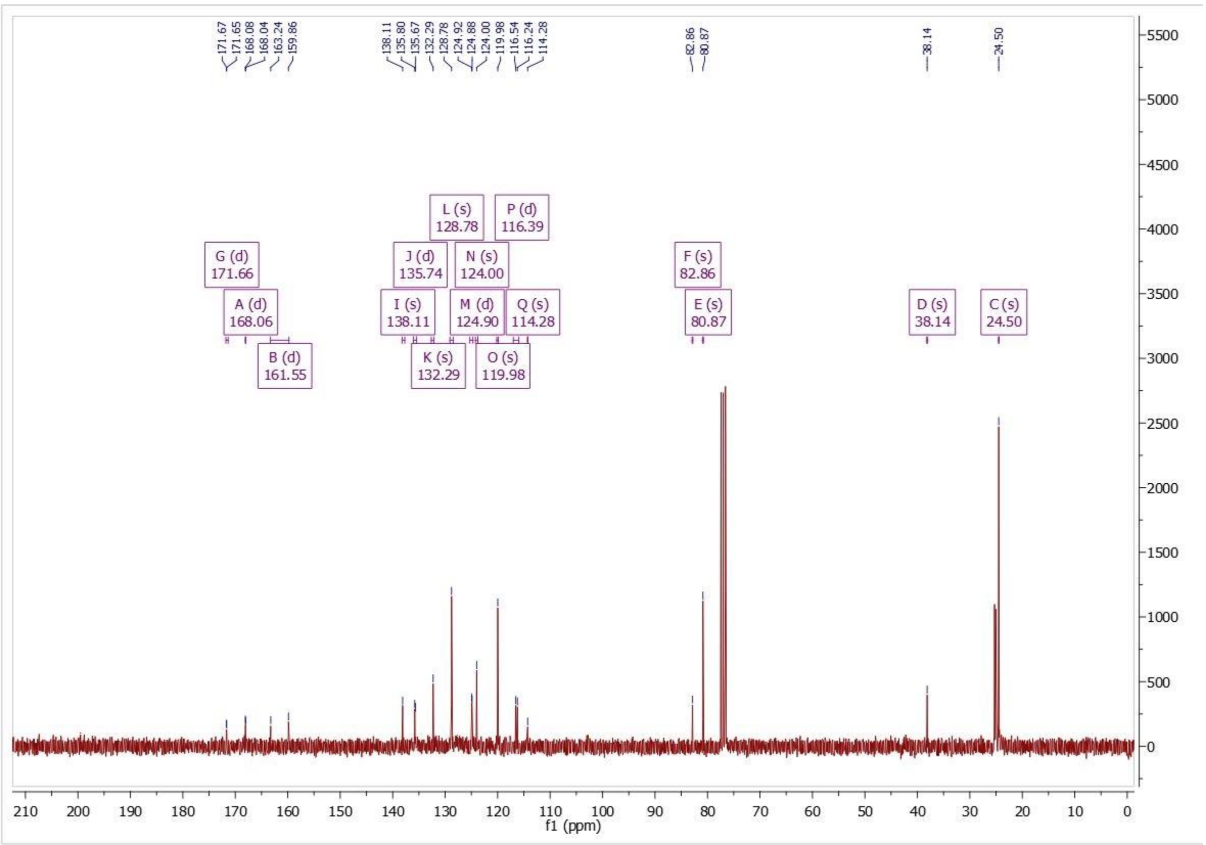

N,*N*-dibenzylprop-2-ynamide (27)

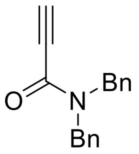

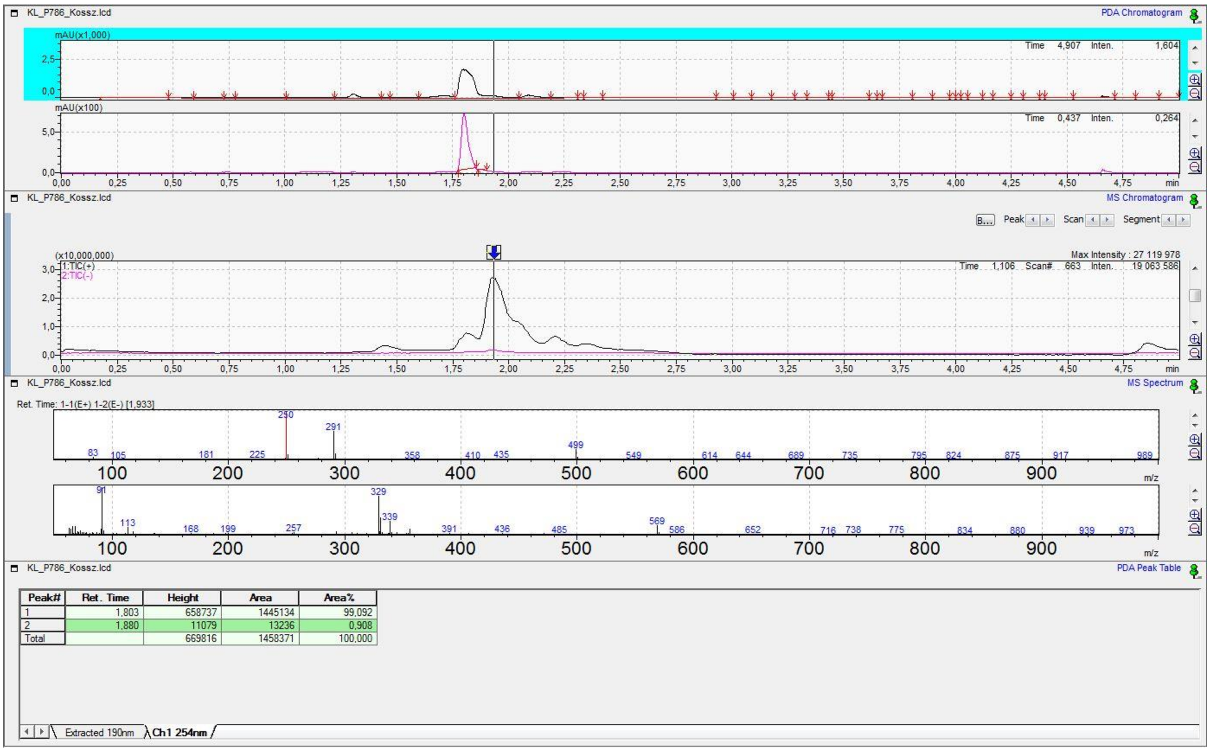
KL_P786_Kossz.lcd
PDA Chromatogram
MS Chromatogram
Max Intensity : 27 119 978
1:TIC(+)
2:TIC(-)
MS Spectrum
Ret. Time: 1-1(E+) 1-2(E-) [1,933]
250
291
499
91
329
339
569
PDA Peak Table
Peak# Ret. Time Height Area Area%
1 1,803 658737 1445134 99,092
2 1,880 11079 13236 0,908
Total 669816 1458371 100,000
Extracted 190nm
Ch1 254nm

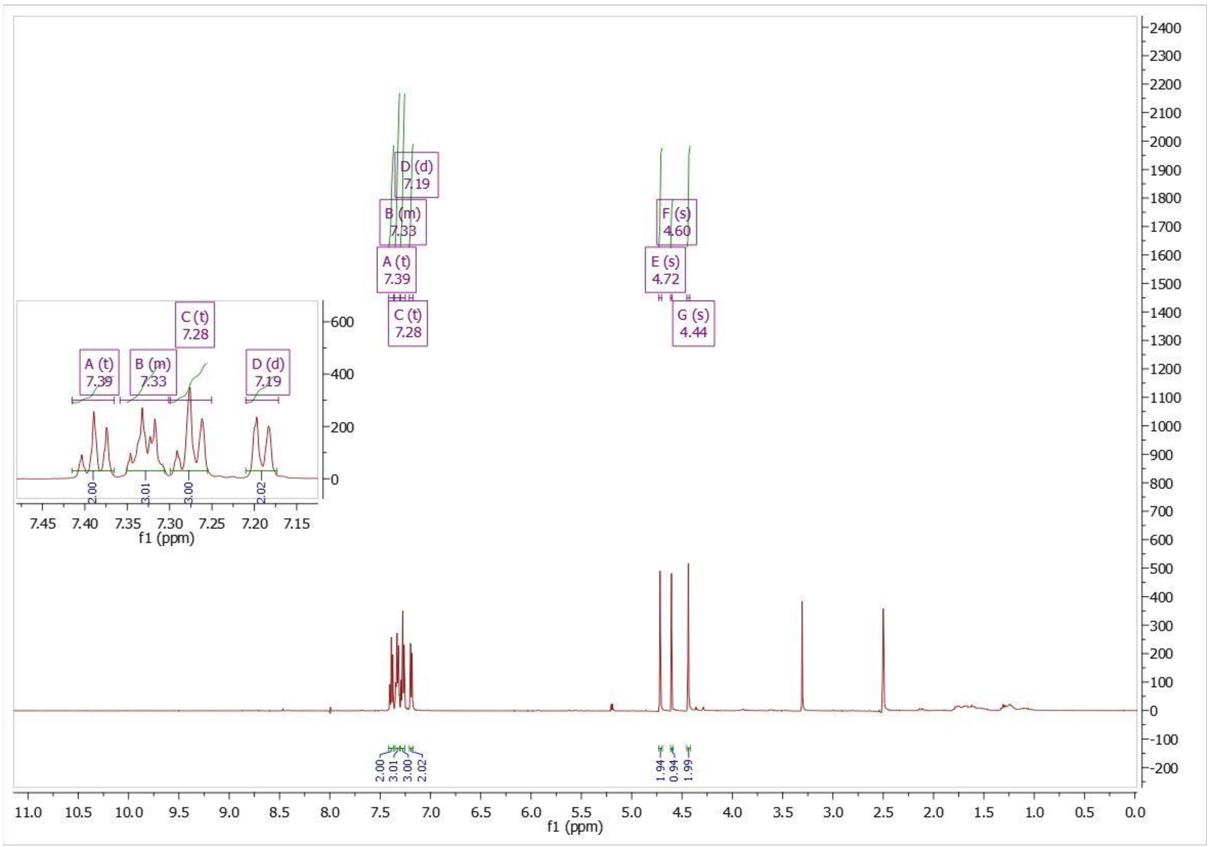
A (t) 7.39
B (m) 7.33
C (t) 7.28
D (d) 7.19
E (s) 4.72
F (s) 4.60
G (s) 4.44
2.00
3.01
3.00
2.02
1.94
0.94
1.99
f1 (ppm)

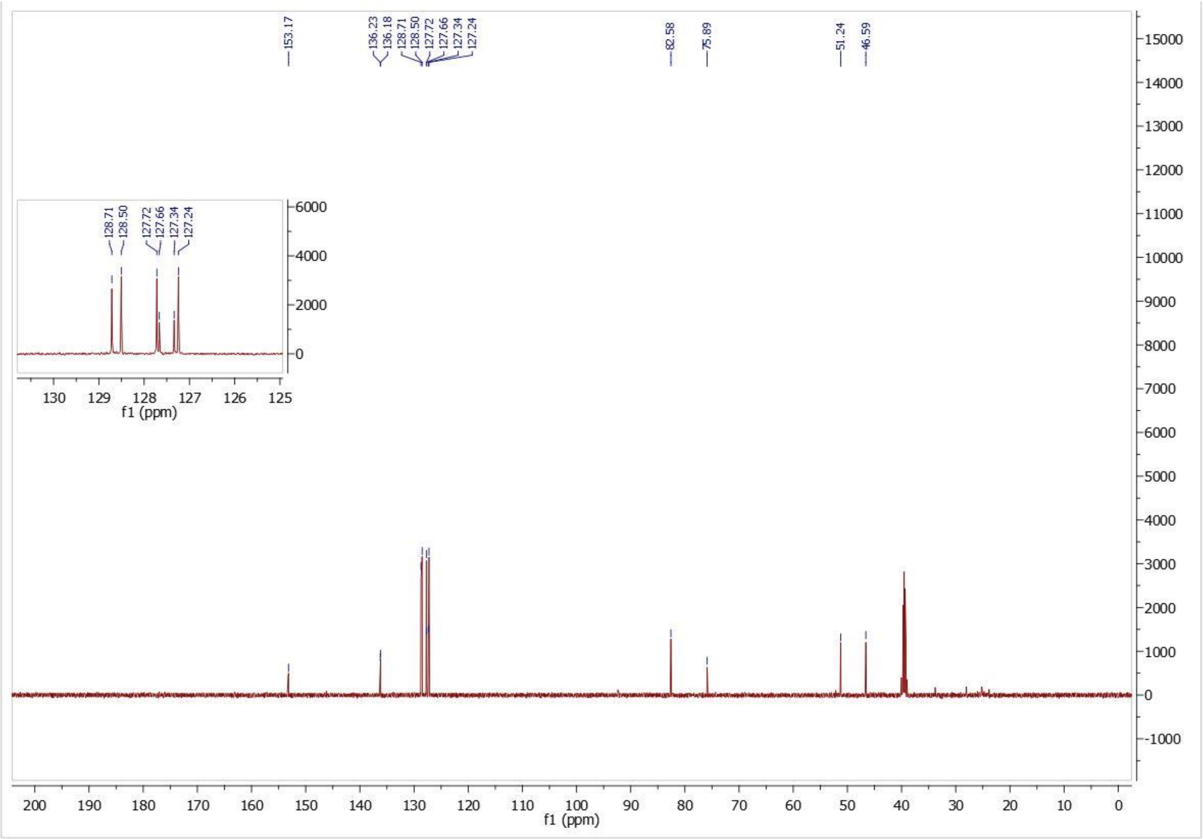

(2*Z*)-*N*,*N*-dibenzyl-3-[(2-fluorophenyl)formamido]prop-2-enamide (28)

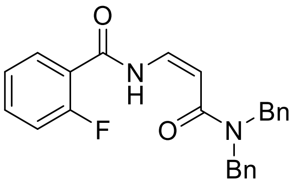

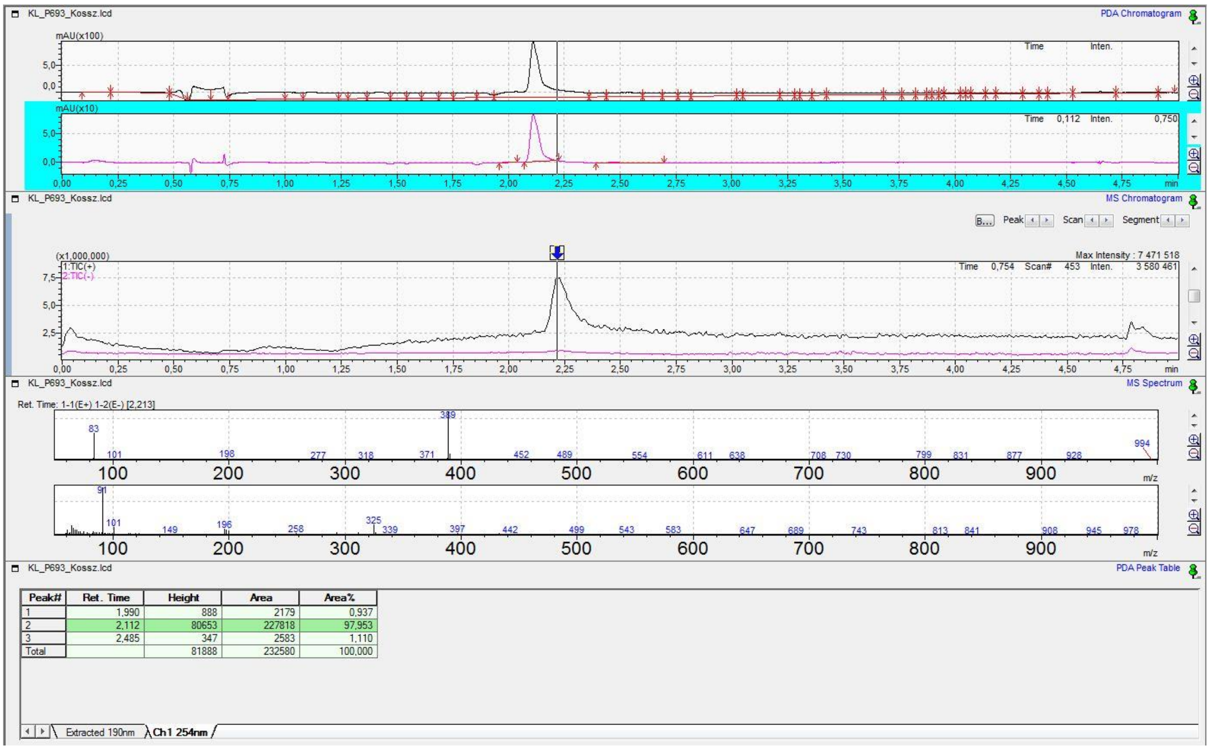

Peak#	Ret. Time	Height	Area	Area%
1	1,990	888	2179	0,937
2	2,112	80653	227818	97,953
3	2,485	347	2583	1,110
Total		81888	232580	100,000

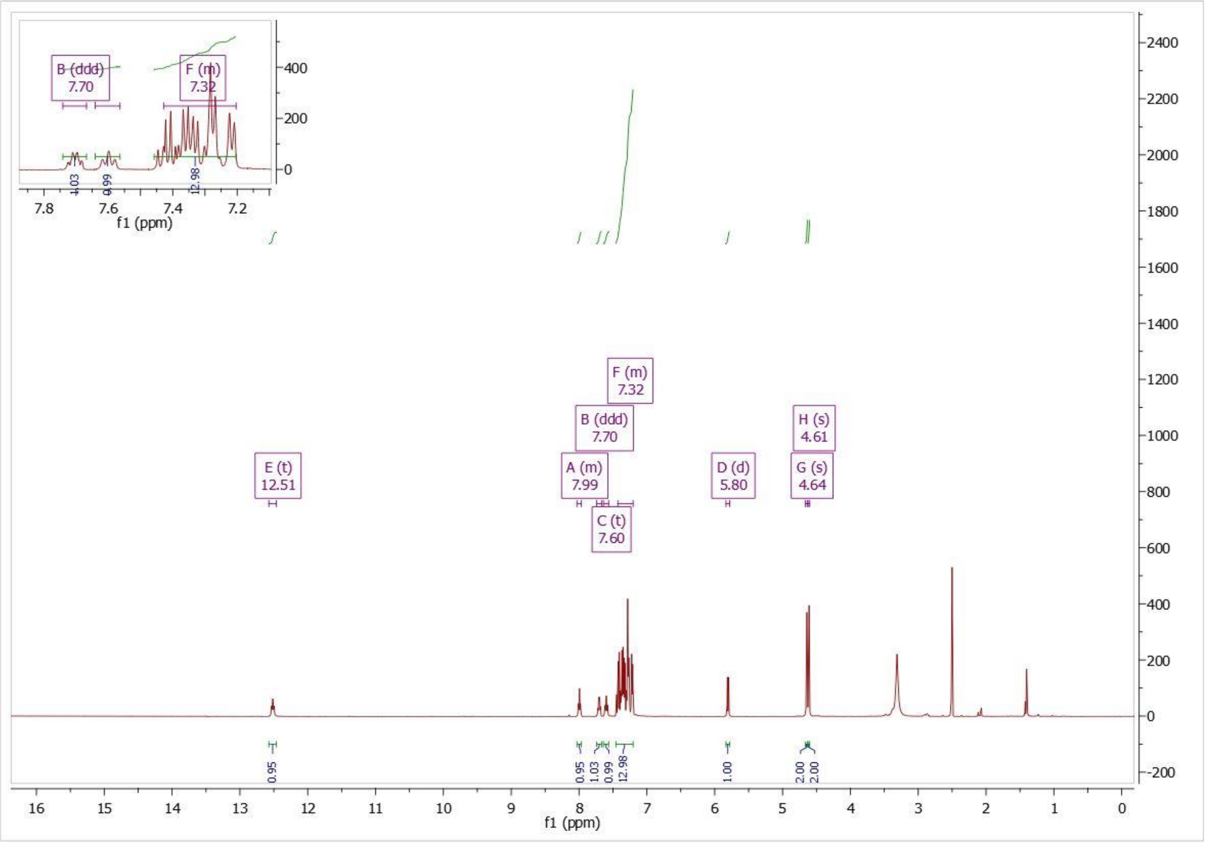

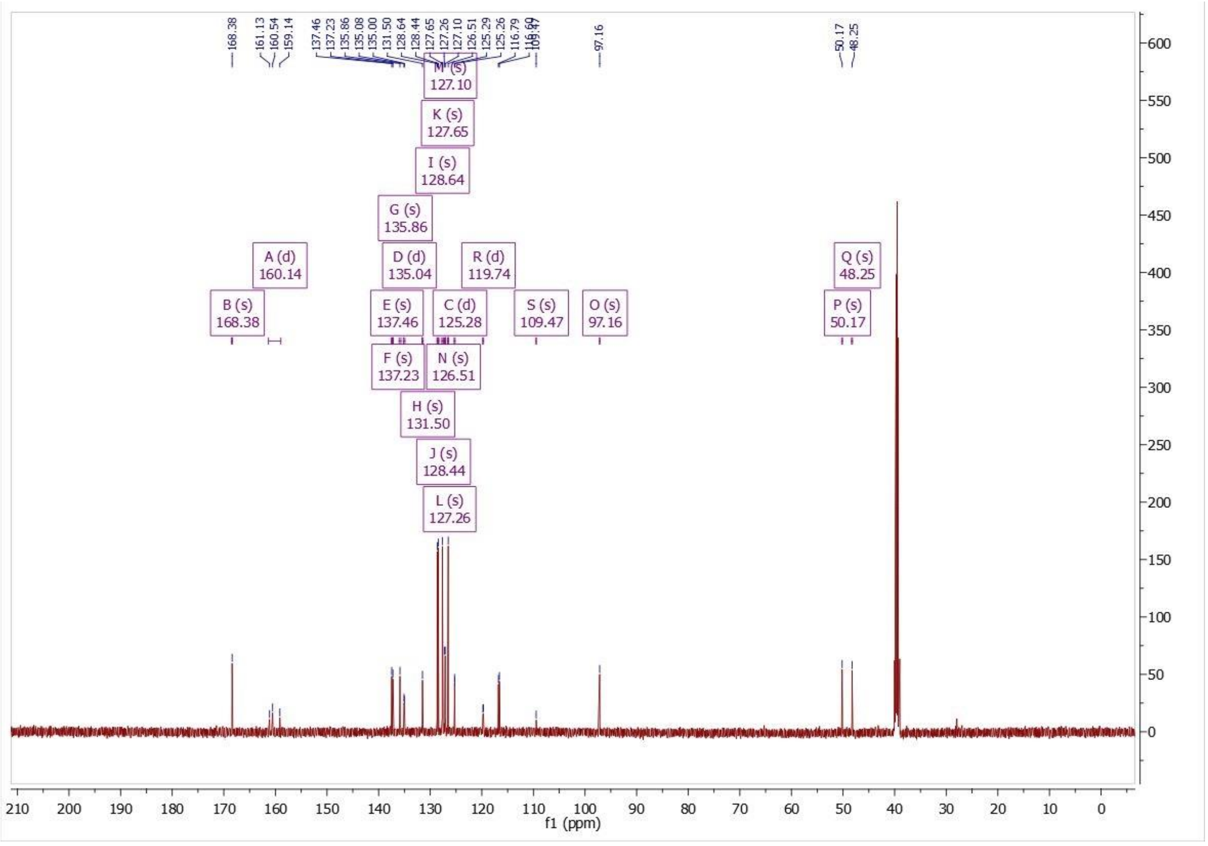

***N*,*N*-dibenzyl-3-[(2-fluorophenyl)formamido]-3-(4,4,5,5-tetramethyl-1,3,2-dioxaborolan-2-yl)propanamide (18)**

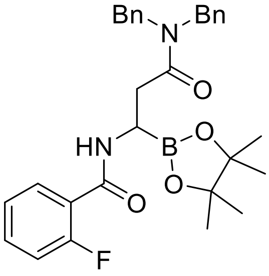

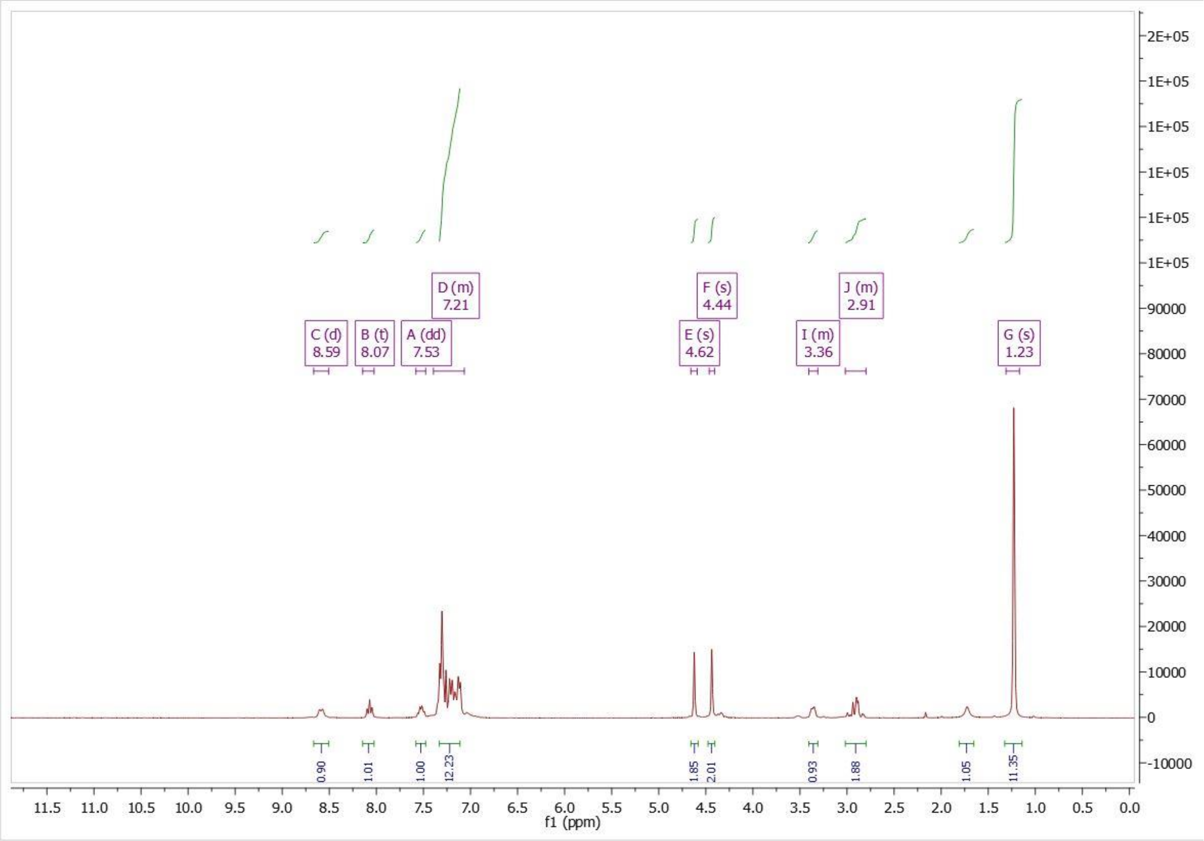
D (m)
7.21
F (s)
4.44
J (m)
2.91
C (d)
8.59
B (t)
8.07
A (dd)
7.53
E (s)
4.62
I (m)
3.36
G (s)
1.23
0.90
1.01
1.00
12.23
1.85
2.01
0.93
1.88
1.05
11.35
f1 (ppm)

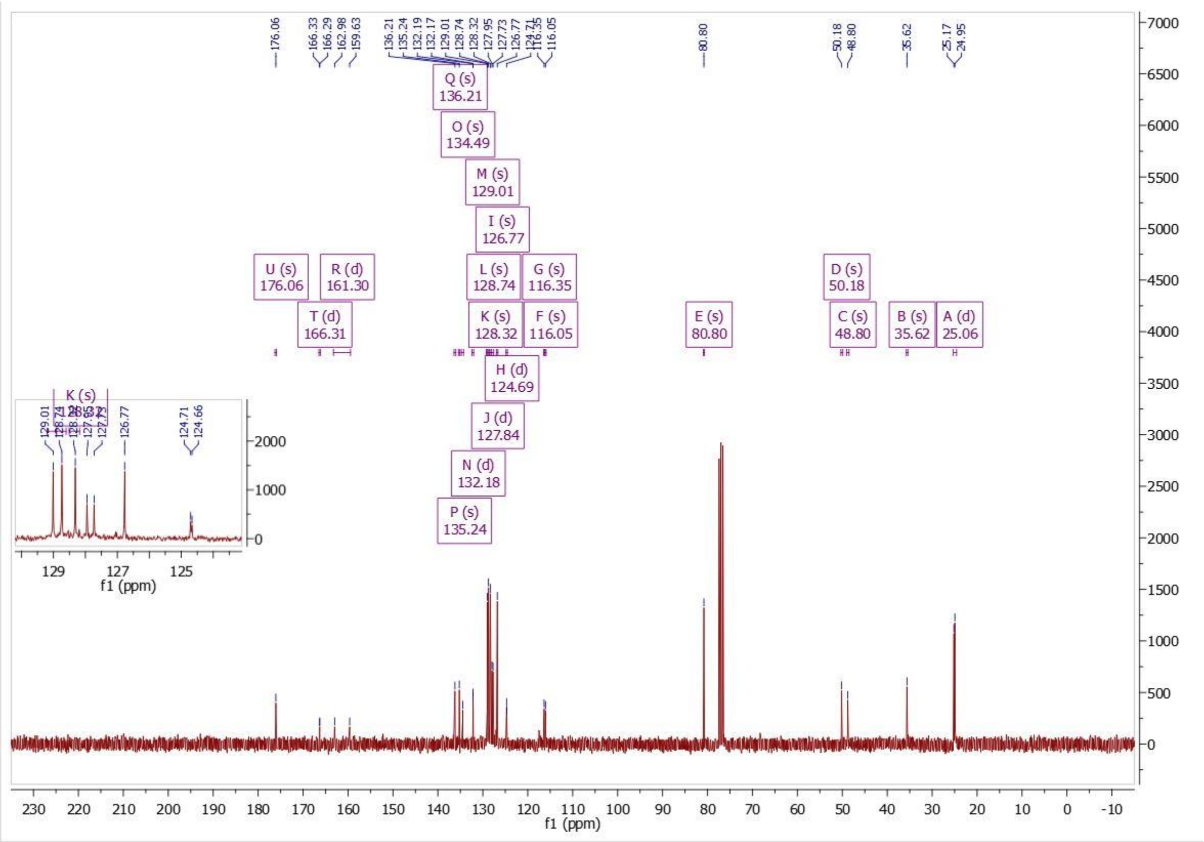
176.06
166.33
166.29
162.98
159.63
136.21
135.24
132.19
132.17
129.01
128.74
128.32
127.95
127.73
126.77
124.71
116.35
116.05
80.80
50.18
48.80
35.62
25.17
24.95
Q (s)
136.21
O (s)
134.49
M (s)
129.01
I (s)
126.77
U (s)
176.06
R (d)
161.30
L (s)
128.74
G (s)
116.35
D (s)
50.18
T (d)
166.31
K (s)
128.32
F (s)
116.05
E (s)
80.80
C (s)
48.80
B (s)
35.62
A (d)
25.06
H (d)
124.69
J (d)
127.84
N (d)
132.18
P (s)
135.24
124.71
124.66
f1 (ppm)

[2-(Bibenzylcarbamoyl)-1-[(2-fluorophenyl)formamido]ethyl]boronic acid (19)

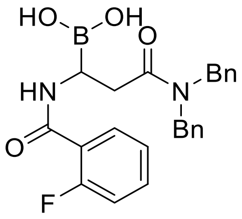

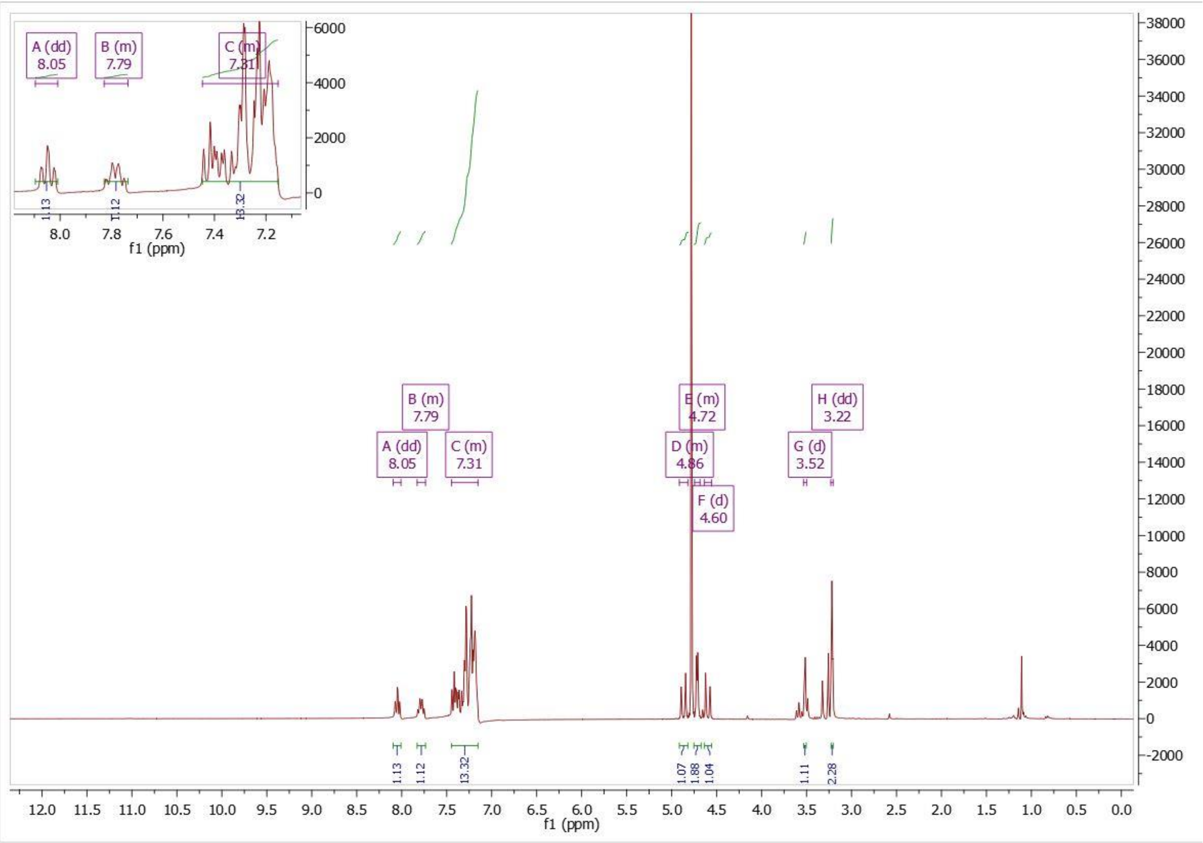

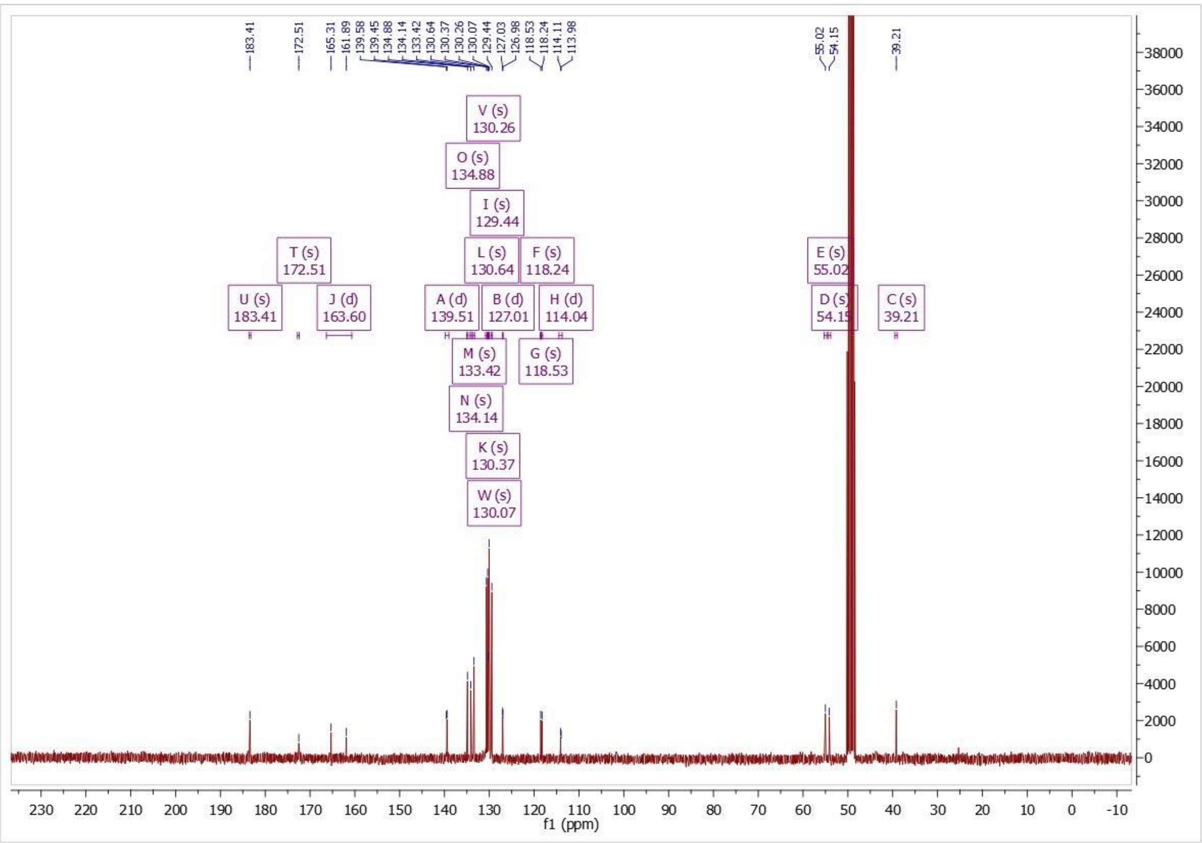

2-[[[2-(4,4,5,5-tetramethyl-1,3,2-dioxaborolan-2-yl)phenyl]methyl]amino]benzaldehyde (32)

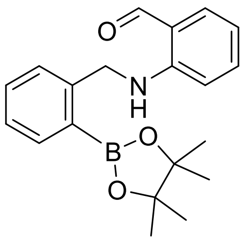

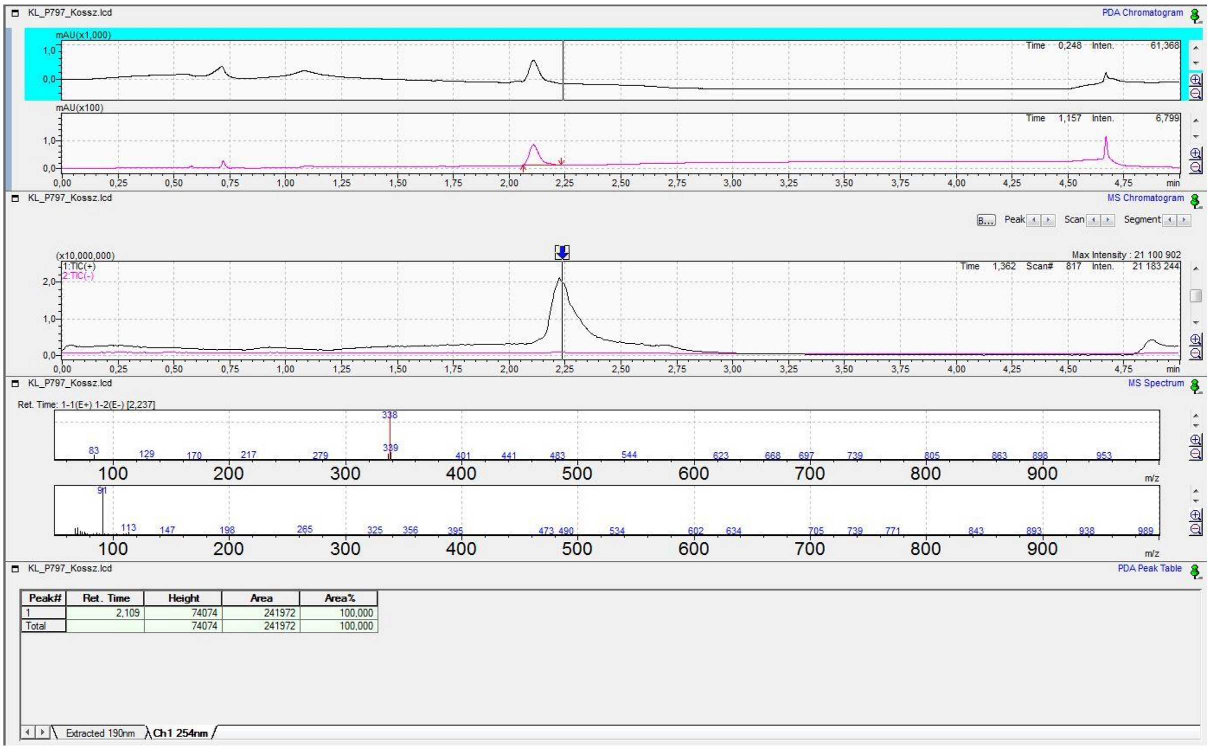
KL_P797_Kossz.lcd
PDA Chromatogram
MS Chromatogram
MS Spectrum
PDA Peak Table
Max Intensity : 21 100 902
Ret. Time: 1-1(E+) 1-2(E-) [2,237]
1:TIC(+)
2:TIC(-)

Peak#	Ret. Time	Height	Area	Area%
1	2,109	74074	241972	100,000
Total		74074	241972	100,000

Extracted 190nm
Ch1 254nm

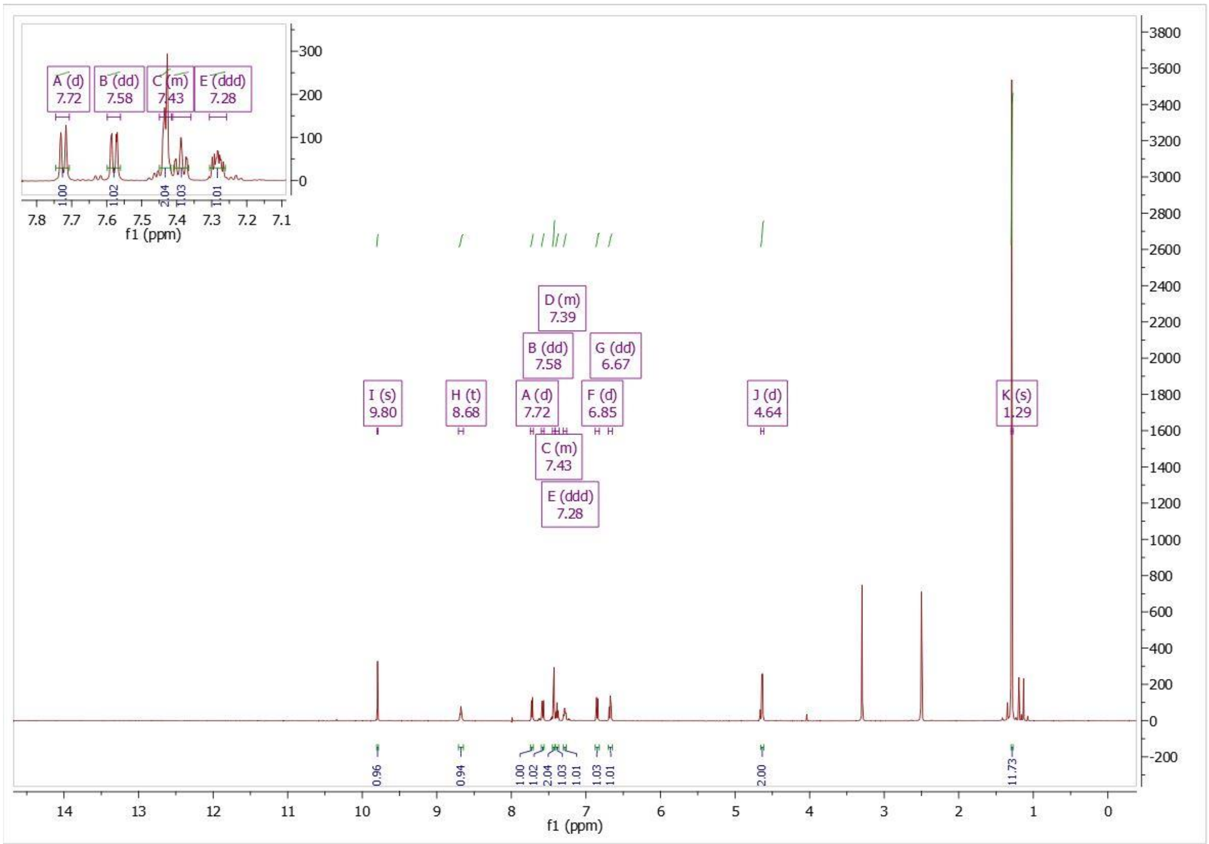
A (d) 7.72
B (dd) 7.58
C (m) 7.43
D (m) 7.39
E (ddd) 7.28
F (d) 6.85
G (dd) 6.67
H (t) 8.68
I (s) 9.80
J (d) 4.64
K (s) 1.29
f1 (ppm)

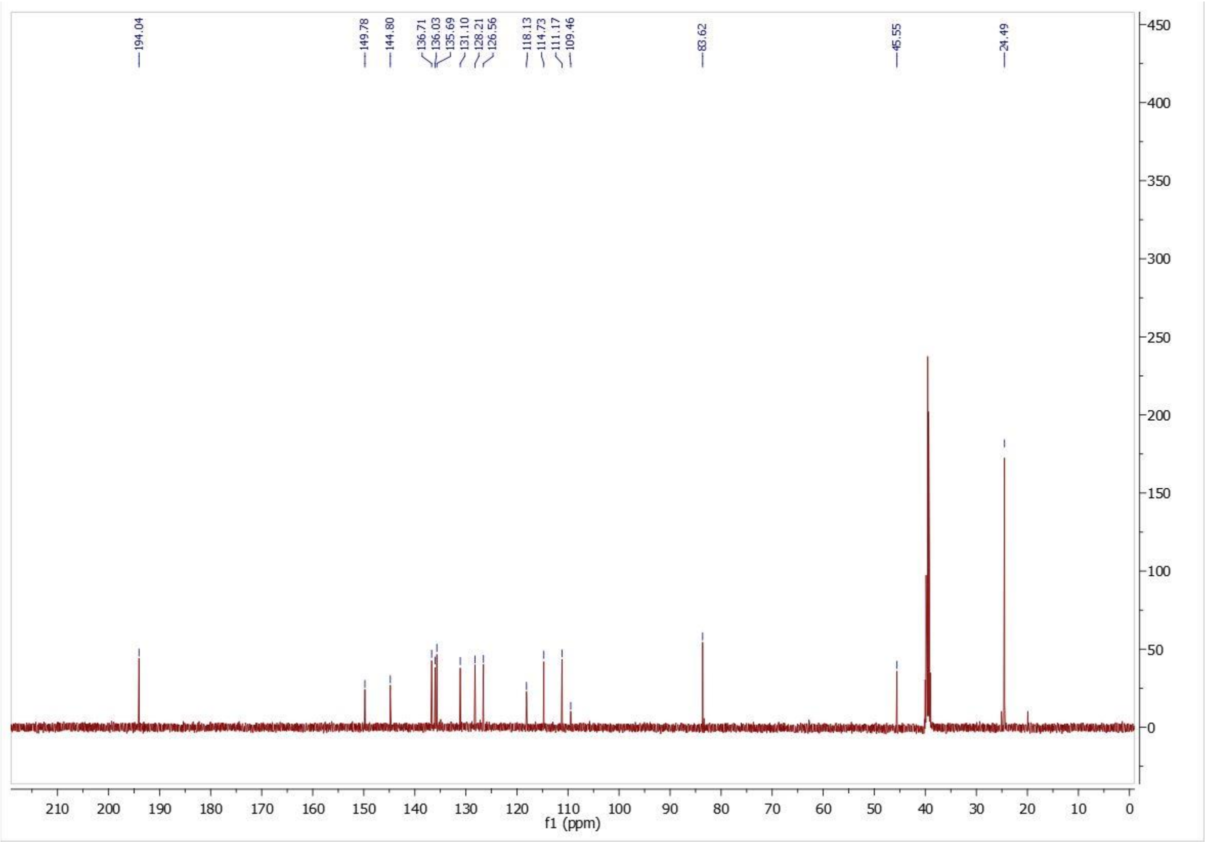

References

[1] A. Zervosen, A. Bouillez, A. Herman, A. Amoroso, B. Joris, E. Sauvage, P. Charlier, A. Luxen, *Bioorganic Med. Chem.* **2012**, *20*, 3915–3924.

[2] S. S. Choudhury, S. Mahapatra, H. S. Biswal, *Green Chem.* **2022**, 4981–4990.

[3] A. López, T. B. Clark, A. Parra, M. Tortosa, *Org. Lett.* **2017**, *19*, 6272–6275.

[4] R. M. Reja, W. Wang, Y. Lyu, F. Haeffner, J. Gao, *J. Am. Chem. Soc.* **2022**, *144*, 1152–1157.

[5] H. Zhang, W. Jiang, P. Chatterjee, Y. Luo, *J. Chem. Inf. Model.* **2019**, *59*, 2093–2102.
